# Hypomyelination, hypodontia and craniofacial abnormalities in a *Polr3b* mouse model of leukodystrophy

**DOI:** 10.1093/brain/awad249

**Published:** 2023-08-28

**Authors:** Mackenzie A Michell-Robinson, Kristin E N Watt, Vladimir Grouza, Julia Macintosh, Maxime Pinard, Marius Tuznik, Xiaoru Chen, Lama Darbelli, Chia-Lun Wu, Stefanie Perrier, Daryan Chitsaz, Nonthué A Uccelli, Hanwen Liu, Timothy C Cox, Christoph W Müller, Timothy E Kennedy, Benoit Coulombe, David A Rudko, Paul A Trainor, Geneviève Bernard

**Affiliations:** Department of Neurology and Neurosurgery, McGill University, Montréal, QC H3A 1A1, Canada; Child Health and Human Development Program, Research Institute of the McGill University Health Centre, Montréal, QC H4A 3J1, Canada; Stowers Institute for Medical Research, Kansas City, MO 64110, USA; Department of Neurology and Neurosurgery, McGill University, Montréal, QC H3A 1A1, Canada; McConnell Brain Imaging Centre, Montreal Neurological Institute and Hospital, Montreal, QC H3A 2B4, Canada; Department of Neurology and Neurosurgery, McGill University, Montréal, QC H3A 1A1, Canada; Child Health and Human Development Program, Research Institute of the McGill University Health Centre, Montréal, QC H4A 3J1, Canada; Translational Proteomics Research Unit, Montreal Clinical Research Institute, Montréal, QC H2W 1R7, Canada; Department of Neurology and Neurosurgery, McGill University, Montréal, QC H3A 1A1, Canada; McConnell Brain Imaging Centre, Montreal Neurological Institute and Hospital, Montreal, QC H3A 2B4, Canada; Department of Neurology and Neurosurgery, McGill University, Montréal, QC H3A 1A1, Canada; Child Health and Human Development Program, Research Institute of the McGill University Health Centre, Montréal, QC H4A 3J1, Canada; Department of Neurology and Neurosurgery, McGill University, Montréal, QC H3A 1A1, Canada; Child Health and Human Development Program, Research Institute of the McGill University Health Centre, Montréal, QC H4A 3J1, Canada; Department of Neurology and Neurosurgery, McGill University, Montréal, QC H3A 1A1, Canada; Child Health and Human Development Program, Research Institute of the McGill University Health Centre, Montréal, QC H4A 3J1, Canada; Department of Neurology and Neurosurgery, McGill University, Montréal, QC H3A 1A1, Canada; Child Health and Human Development Program, Research Institute of the McGill University Health Centre, Montréal, QC H4A 3J1, Canada; Department of Neurology and Neurosurgery, McGill University, Montréal, QC H3A 1A1, Canada; Department of Neurology and Neurosurgery, McGill University, Montréal, QC H3A 1A1, Canada; Department of Neurology and Neurosurgery, McGill University, Montréal, QC H3A 1A1, Canada; McConnell Brain Imaging Centre, Montreal Neurological Institute and Hospital, Montreal, QC H3A 2B4, Canada; Department of Oral and Craniofacial Sciences, School of Dentistry, and Pediatrics, School of Medicine, University of Missouri – Kansas City, Kansas City, MO 64108, USA; Structural and Computational Biology Unit, European Molecular Biology Laboratory (EMBL), 69117 Heidelberg, Germany; Department of Neurology and Neurosurgery, McGill University, Montréal, QC H3A 1A1, Canada; Translational Proteomics Research Unit, Montreal Clinical Research Institute, Montréal, QC H2W 1R7, Canada; Department of Biochemistry and Molecular Medicine, University of Montréal, Montréal, QC H3C 3J7, Canada; Department of Neurology and Neurosurgery, McGill University, Montréal, QC H3A 1A1, Canada; McConnell Brain Imaging Centre, Montreal Neurological Institute and Hospital, Montreal, QC H3A 2B4, Canada; Department of Biomedical Engineering, McGill University, Montréal, QC H3A 2B4, Canada; Stowers Institute for Medical Research, Kansas City, MO 64110, USA; Department of Anatomy and Cell Biology, The University of Kansas School of Medicine, Kansas City, KS 66160, USA; Department of Neurology and Neurosurgery, McGill University, Montréal, QC H3A 1A1, Canada; Child Health and Human Development Program, Research Institute of the McGill University Health Centre, Montréal, QC H4A 3J1, Canada; Department of Pediatrics, McGill University, Montréal, QC H4A 3J1, Canada; Department of Human Genetics, McGill University, Montréal, QC H4A 0C7, Canada; Department of Specialized Medicine, Division of Medical Genetics, Montreal Children’s Hospital and McGill University Health Centre, Montréal, QC H4A 3J1, Canada

**Keywords:** neurodevelopment, myelinogenesis, POLR3B, POLR3-related leukodystrophy, 4H leukodystrophy, mouse model

## Abstract

RNA polymerase III (Pol III)-related hypomyelinating leukodystrophy (POLR3-HLD), also known as 4H leukodystrophy, is a severe neurodegenerative disease characterized by the cardinal features of hypomyelination, hypodontia and hypogonadotropic hypogonadism. POLR3-HLD is caused by biallelic pathogenic variants in genes encoding Pol III subunits. While approximately half of all patients carry mutations in *POLR3B* encoding the RNA polymerase III subunit B, there is no *in vivo* model of leukodystrophy based on mutation of this Pol III subunit.

Here, we determined the impact of *POLR3B*Δ10 (Δ10) on Pol III in human cells and developed and characterized an inducible/conditional mouse model of leukodystrophy using the orthologous Δ10 mutation in mice. The molecular mechanism of Pol III dysfunction was determined in human cells by affinity purification-mass spectrometry and western blot. Postnatal induction with tamoxifen induced expression of the orthologous Δ10 hypomorph in triple transgenic *Pdgfrα-Cre/ERT; R26-Stop^fl^-EYFP; Polr3b^fl^* mice. CNS and non-CNS features were characterized using a variety of techniques including microCT, *ex vivo* MRI, immunofluorescence, immunohistochemistry, spectral confocal reflectance microscopy and western blot. Lineage tracing and time series analysis of oligodendrocyte subpopulation dynamics based on co-labelling with lineage-specific and/or proliferation markers were performed.

Proteomics suggested that Δ10 causes a Pol III assembly defect, while western blots demonstrated reduced POLR3BΔ10 expression in the cytoplasm and nucleus in human cells. In mice, postnatal *Pdgfrα*-dependent expression of the orthologous murine mutant protein resulted in recessive phenotypes including severe hypomyelination leading to ataxia, tremor, seizures and limited survival, as well as hypodontia and craniofacial abnormalities. Hypomyelination was confirmed and characterized using classic methods to quantify myelin components such as myelin basic protein and lipids, results which agreed with those produced using modern methods to quantify myelin based on the physical properties of myelin membranes. Lineage tracing uncovered the underlying mechanism for the hypomyelinating phenotype: defective oligodendrocyte precursor proliferation and differentiation resulted in a failure to produce an adequate number of mature oligodendrocytes during postnatal myelinogenesis.

In summary, we characterized the *Polr3b*Δ10 mutation and developed an animal model that recapitulates features of POLR3-HLD caused by *POLR3B* mutations, shedding light on disease pathogenesis, and opening the door to the development of therapeutic interventions.

## Teaser

Michell-Robinson *et al.* present the first leukodystrophy mouse model based on mutation of *Polr3b*, a gene encoding a subunit of RNA polymerase III (Pol III). The clinical manifestations of disease in the model, including hypomyelination, hypodontia and craniofacial abnormalities, resemble those seen in patients with leukodystrophy caused by *POLR3B* mutations.

## Introduction

POLR3-related hypomyelinating leukodystrophy (POLR3-HLD) is one of the most common hypomyelinating leukodystrophies. It is caused by biallelic pathogenic variants in genes encoding subunits of the RNA polymerase III (Pol III) complex; most commonly RNA polymerase III subunit A (*POLR3A*) and *POLR3B*, but also RNA polymerase I subunit C (*POLR1C*) and *POLR3K*.^1-6^ POLR3-HLD presents in previously healthy children in the first few years of life, leading to progressive disability and early death.^7^ The classic presentation includes hypomyelination, hypodontia and hypogonadotropic hypogonadism.^7-10^ While neurodegeneration is thought to be primarily driven by hypomyelination with secondary axonal loss, the disease pathogenesis is poorly understood.^10-14^ We hypothesize that Pol III hypofunction during a critical developmental period causes defective myelinogenesis and hypomyelination.^15^

Efforts to model POLR3-HLD *in vivo* using constitutive patient-derived mutations in *POLR3A* and *POLR3B* have been unsuccessful, owing either to a lack of phenotype or embryonic lethality.^16,17^ Recently, Merheb *et al.*^18^ developed an Olig2 conditional knock-in mouse model using a constitutively lethal double mutation in *Polr3a*.^19^ The model displays recessive characteristics including mild cerebral hypomyelination and neurobehavioral abnormalities but does not have gross motor defects, cerebellar hypomyelination, or non-neurological features.^18,19^ Because these are all typical features of the classic human disease, there was a clear need to develop and characterize a more representative disease model for developing therapeutic approaches in future preclinical studies. Nearly half of the patients with POLR3-HLD carry mutations in *POLR3B* and there is no working model of leukodystrophy based on that gene, so we elected to use *Polr3b* for our murine model.

Developing a leukodystrophy mouse model based on Pol III subunit mutations is not trivial. Constitutively-expressed mutations have not generated an appropriate level of phenotypic severity due to Pol III being necessary for survival during embryonic development.^16-18^ Fortunately, constitutively lethal mutations can be expressed in a tissue- and/or time-dependent manner using Cre/lox technology to override the survival requirement for critical genes.^18,20,21^ An important question when developing such models is how a particularly severe mutant affects its milieu and the degree to which its effects resemble those of the disease one is attempting to replicate. Since we were interested in developing a leukodystrophy model based on a *Polr3b* mutation, it was important to make sure that the selected mutant impacted Pol III in a similar manner to patient-derived mutations.

Disease-causing mutations in Pol III subunits impact subunit expression/stability, Pol III biogenesis (complex assembly), nuclear import, and transcriptional activity.^1,3,16,17,22,23^ We became interested in a known *Polr3b* exon 10 deletion mutant (Δ10) because of its predicted impact on Pol III biogenesis.^24^ The Δ10 mutant was discovered in a mutagenesis screen and initially described as the *polr3b* splice mutant *SlimJim* (*Slj*), which produces a gut phenotype in zebrafish.^24^ To further understand the mutation, a mouse with a floxed exon 10 allele was generated for studying the gut using the Villin-Cre system, but was not evaluated in other tissues.^25^ Δ10 has not specifically been detected in POLR3-HLD patients but *POLR3B* exon deletions have been reported,^26^ as have missense (M243V, G244V, S268G) and nonsense/frameshift mutations in exon 10.^8,10,27-31^ Furthermore, intronic variants that alter splicing leading to exon skipping in the *POLR3B* transcript have been reported.^8,10,29,32,33^ Taken together, there was sufficient literature supporting the notion that both Δ10 and disease-causing *POLR3B* mutants had pathophysiological similarities at the gene and protein level, which made Δ10 an interesting candidate for further study in the context of POLR3-HLD.

We set out to characterize Δ10 at the molecular level in human cells in order to understand its effects on Pol III. Our proteomics studies suggested that Δ10 expression caused defective Pol III complex assembly, which was accompanied by reduced expression of the mutant protein in the cytosol and nucleus of human cells, like other known POLR3-HLD mutations.^3,17^ We then developed an inducible/conditional expression model *in vivo*, whereby the orthologous Δ10 mutation was induced in a temporal and *Pdgfrα*-dependent manner during early postnatal development in mice. Expressing Δ10 in this model led to recessive phenotypes, which were reminiscent of the cardinal manifestations of POLR3-HLD, including severe hypomyelination, hypodontia and craniofacial abnormalities. In-depth characterization of the CNS phenotype allowed us to answer fundamental questions about the development of severe hypomyelination in our model, such as whether the primary mechanism was reduced numbers of myelinating cells or reduced production of myelin by a normally sized pool of oligodendrocytes. We found that an early proliferation defect prevented the accumulation of adequate numbers of oligodendrocyte precursors, which was followed by a maturation defect in these precursors, suggesting that putative POLR3-HLD therapeutics will need to target oligodendrocyte precursors to correct hypomyelination.

## Materials and methods

Detailed materials and methods for all sections are provided in the Supplementary material.

### Proteomics

DNA plasmids were constructed and cloned in p3XFLAG-CMV14 expression plasmids (Sigma). All plasmid sequences were verified by sequencing at each construction step. HEK293T cells (1.6 × 10^7^) were transiently transfected for 24 h with 8.51 µg of plasmid containing human wild-type or mutant *POLR3B* in 15 cm plates using Jet Prime (PolyPlus) according to the manufacturer's recommended protocol. Cells were harvested, washed with 1× PBS, and snap frozen in liquid nitrogen. The FLAG affinity purification protocol was modified from Kean *et al.*^34^ to produce samples for LC-MS/MS. High performance liquid chromatography (HPLC) was performed in a 75 μm i.d. × 150 mm Self-Pack C18 column installed in the Easy-nLC II system (Proxeon Biosystems). HPLC was coupled to an Orbitrap Fusion mass spectrometer (Thermo Scientific) through a Nanospray Flex Ion Source (ThermoFisher, ES071). Label-free quantification (LFQ) intensity for each protein was obtained using MaxQuant (version 1.6.0.16) against the characterized human UniProtKB database (released on 3 June 2018).^35,36^ Log2 transformation, imputation and further statistical analysis were performed with Perseus (version 1.6.14.0).^37^ All purifications were done in triplicate and proteins detected in all experiments were kept for further analysis. Missing values were replaced by randomly generated intensities normally distributed with a width of 0.3 times and a downshift of 1.8 times the standard deviation (SD) of non-zero intensities. Significant differences between Log2 protein intensities from bait purifications and the control groups were then determined using a two-tailed *t*-test subsequently adjusted for multiple hypothesis testing with a permutation-based false discovery rate (FDR) of 0.05 and an s0 factor of 0.1 with 10 000 iterations. For all analyses, q-value ≤ 0.05 was considered statistically enriched.

### Transgenic mice

Animal husbandry and all studies described in the publication including animal euthanasia adhered to our animal care and use protocol, as approved by the McGill University Health Centre Animal Resource Division (AUP8055). Parental strains used in the study were *Pdgfrα-Cre/ERT* (‘*Pdgfrα-CreERT*’),^38^*Polr3b^fl^* (floxed exon 10)^25^ and *R26-stop^fl^-EYFP* (‘*YFP*’).^39^ Unless otherwise indicated, pups used in the study were generated from crosses between a *Pdgfrα-CreERT; YFP; Polr3b^+/fl^* male and a *Polr3b^fl/fl^* female. Tamoxifen-treatment induced conversion of unrecombined *Polr3b^fl^* wildtype-equivalent allele into an exon 10 deletion allele, i.e. ‘Δ10’. Therefore, *Pdgfrα-CreERT; YFP; Polr3b^fl/fl^*, *Pdgfrα-CreERT; YFP; Polr3b^+/fl^*, and *YFP; Polr3b^fl/fl^* or *YFP; Polr3b^+/fl^* (Cre-negative control) mice were referred to as Δ10/Δ10, +/Δ10 and CTRLs, respectively, when treated with tamoxifen. By convention, we have referred to mouse proteins in this article using title case (first letter capitalized) to differentiate them from human proteins (all capitals) without referring to species explicitly (e.g. Mbp versus MBP).

### Tissue histology

Mice underwent transcardiac perfusion under isofluorane anaesthesia with PBS followed by a fixative for fresh frozen (FF) or formalin-fixed paraffin embedded (FFPE) block preparation. Tissues were harvested and sections prepared according to standard protocols for each method. Images of haematoxylin and eosin stained FFPE sections were acquired on a Leica Aperio AT Turbo digital whole slide scanning system at 20× resolution. Fluorescence images were acquired using a Zeiss Axioscan Z1 slide scanner employing a plan-apochromat 10× objective and AxioCam MR R3 camera.

### Electrophoresis and immunoblotting

Brain tissue was prepared by dissection with hindbrain (cerebellum, pons, medulla) removed. Tissue lysates were prepared according to standard protocols in RIPA buffer with fresh protease inhibitors, normalized for protein concentration, and run on tris-glycine gels freshly prepared according to the Laemmli protocol with 0.5% trichloroethanol modification.^40^ Activated proteins were transferred to PVDF support using the Bio-Rad Transblot Turbo, developed in Clarity Max enhanced chemiluminescence substrate, and imaged according to Bio-Rad manufacturer's protocols.

### MRI

Paraformaldehyde (PFA)-fixed brains of 16 juvenile mice (P18) were used in this study. All imaging was performed using the Bruker Pharmascan 7 T pre-clinical MRI system. Multi-echo gradient-recalled-echo (mGRE) T_2_*-weighted image volumes were acquired using a 3D mGRE sequence with bipolar readout. Reconstructed 4D volumes were corrected for bipolar gradient induced mis-registration and Gibbs ringing prior to calculation of myelin water fraction (MWF) images.^41,42^ Whole-brain MWF maps were calculated by fitting the three-pool relaxation model of white matter using a recently developed self-labelled encoder decoder (SLED) network implemented in PyTorch.^43,44^ Region of interest (ROI) parcellation was achieved by computing affine and symmetric diffeomorphic transformations between the mGRE volumes and an anatomically labelled reference atlas.^45^

### Cell counting

Histological images were exported from Zeiss proprietary format to full depth 16-bit tiff images in FIJI (ImageJ). Binary image masks were manually delineated in FIJI and saved as tiff images. Image processing pipelines were developed in CellProfiler 4.2.1 using representative images.^46-50^ Images and CellProfiler pipelines were exported to Amazon Web Services and imaging pipelines were run using Distributed CellProfiler 2.0.0_4.1.3. The results were collated in SqLite databases and subsequently analysed in CellProfiler Analyst 3.0.4.

### MicroCT

P18 mice were euthanized and stored in 95% ethanol until imaging. Mice were imaged at 15-μm resolution using a Skyscan 1275 micro-computed tomography (microCT) scanner (Bruker). Raw scan data were reconstructed using NRecon and 3D rendered for visual assessment and measurement in Drishti v3.0 as previously described.^51^ All scans (55 kV, 0.5 mm AI filter, 45 ms exposure, 0.3° rotation step, four frame averaging), reconstruction and rendering settings were kept consistent between specimens.

### Statistics

Statistical analyses were performed with GraphPad Prism 9.4.1 or SPSS v28 0.1.1 (IBM). Biological replicates are presented as individual points in all graphs unless otherwise indicated. For brevity, statistical analyses (tests), *n*, α, and other mathematical details pertaining to analysis and/or comparisons are detailed in the accompanying figure legends.

## Results

### Human Δ10 impairs Pol III complex assembly or stability

**Figure 1:**
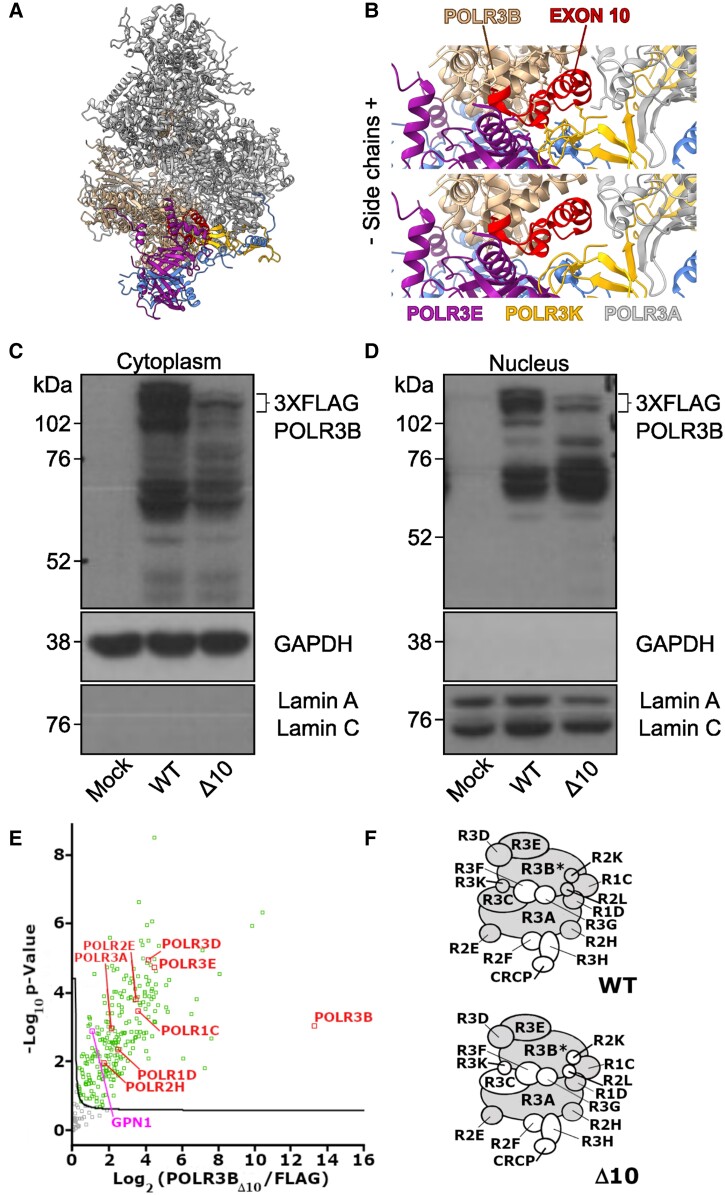
***POLR3B* exon 10 deletion impairs Pol III assembly or stability in human cells.** (**A**) Human Apo Pol III structural model. (**B**) Human Apo Pol III rotated and zoomed with POLR3B exon 10 highlighted in red and nearby subunits labelled including heterodimer subunit POLR3E, POLR3K and POLR3A (jaw). (**C**) Representative anti-FLAG western blot of cytoplasmic extract from HEK293T cells transiently transfected with p3XFLAG (mock), wild-type POLR3B-FLAG (WT) or POLR3BΔ10-FLAG (Δ10) for 24 h. Loading controls for the cytoplasmic (GAPDH) and nuclear (Lamin A, C) fractions are shown. (**D**) Representative anti-FLAG western blot of nuclear extract from HEK293T cells transiently transfected with p3XFLAG (mock), wild-type POLR3B-FLAG (WT) or POLR3BΔ10-FLAG (Δ10) for 24 h. Loading controls for the cytoplasmic (GAPDH) and nuclear (Lamin A, C) fractions are shown. (**E**) Volcano plot illustrating the log2-transformed average label-free quantification (LFQ)-intensity difference between the Δ10 mutant against the FLAG expressing control (*x*-axis). The significantly different Pol III subunits are marked in red and the significantly different GPN cofactors in pink. Other proteins marked in green are considered significantly different. (**F**) Schematic representation showing significantly different subunits evaluated by AP-MS. Wild-type data from Djordjevic *et al*.^53^ were used to show expected Pol III subunits. Subunits that are significantly increased compared to their FLAG control are marked in grey. Subunits that are not detected or not significantly different from the FLAG control are marked in white. *The subunit used as bait.

The mechanism by which Δ10 impacts Pol III function is unknown. We therefore endeavoured to characterize the Δ10 mutation in human cells to understand its impact on POLR3B protein expression as well as assembly and nuclear import of the mutant Pol III complex. First, we modelled the Δ10 mutation on the human Pol III cryo-EM structure and predicted that this deletion affects a central part of POLR3B (p.A242-Q282del).^52^ POLR3B exon 10 binds POLR3K and the jaw of POLR3A, suggesting that Δ10 would impair Pol III complex assembly or stability (Fig. 1A and B). To demonstrate this, POLR3B wild-type (WT) and Δ10 were cloned into separate 3XFLAG plasmids and transiently transfected into HEK293T cells. Anti-FLAG western blot analysis indicated that under identical conditions, 3XFLAG-POLR3BΔ10 (FLAG-Δ10) protein was not as highly expressed as 3XFLAG-POLR3B (FLAG-WT) protein (Fig. 1C), suggesting that FLAG-Δ10 (transcript or protein) is less stable than FLAG-WT. We next sought to determine if the FLAG-Δ10 protein could be found in nuclear extracts from plasmid-treated cells, as Pol III is assembled in the cytosol and imported to the nucleus for transcription. We detected FLAG in nuclear extracts at the expected molecular weight of FLAG-Δ10, demonstrating that FLAG-Δ10 was able to form complexes competent for import (Fig. 1D). Using affinity purification coupled to mass spectrometry (AP-MS), we were able to determine, by comparing the POLR3BΔ10 mutant to the negative control FLAG alone, that some Pol III subunits were absent from the 3XFLAG-POLR3BΔ10 complex (Fig. 1E), as summarized by the model shown in Fig. 1F. Our findings verified that Δ10 expression impaired Pol III complex assembly or stability and reduced POLR3B protein expression in human cells.

### Developing a Pdgfrα*-*dependent Δ10 expression model *in vivo*

*POLR3B* mutations resulting in Pol III complex assembly defects are known to be causal in POLR3-HLD.^17^ Therefore, we elected to study the Δ10 mutation *in vivo* in the context of oligodendrocyte precursor cell (OPC) development using an inducible/conditional *Pdgfrα-CreERT* system. To target Δ10 expression to the largest group of OPCs possible without toxicity, we validated a postnatal tamoxifen injection protocol in control mice. Dosing trials on wild-type animals (Supplementary Fig. 1A) allowed us to select daily injections of tamoxifen at 40 mg/kg from P2–P5 as our dosing regimen.

By crossing the *Pdgfrα-CreERT* and *YFP* founder strains, we generated double heterozygous control mice and assessed tamoxifen recombination efficiency. Administration of tamoxifen according to the protocol defined above demonstrated that 55–98% of Olig2+ cells were also YFP+ at P9, depending on the brain region. All regions analysed except the cortex maintained a recombination efficiency of over 55% until P21 (Supplementary Fig. 1B). This experiment also independently validated that the *Pdgfrα-CreERT* allele produced high-fidelity recombination in the oligodendrocyte lineage, as >90% of YFP+ cells were also Olig2+ in histological sections at P9 and P14 with expected recombination also observed in capillary endothelium and choroid plexus (not shown).^38^

We developed a *Pdgfrα-CreERT; YFP;Polr3b* triple transgenic model to express Δ10 and *YFP* in *Pdgfrα+* cells. Genotyping PCRs and validation sequencing of the founder strains yielded the expected results (Supplementary Fig. 1C–E).^25,38,39^ In <5% of total live births, we detected germ line transmission of a Δ10 allele by PCR. Pups with germ-line recombination were screened out of subsequent studies. Brain recombination was validated by genotyping PCR using tissue from *Pdgfrα-CreERT;Polr3b^+/fl^* and *Pdgfrα-CreERT;Polr3b^fl/fl^* double transgenic mice (Supplementary Fig. 1F). Expression of the Δ10 mRNA was subsequently validated by RT-PCR in tamoxifen-treated +/Δ10 mice (P30), and the products were sequenced confirming the integrity of the exon 9–11 boundary in spliced mRNA. All subsequent studies were carried out with triple transgenic mice.

### Pdgfrα*-*dependent Δ10 expression produces recessive phenotypes consistent with POLR3-HLD

**Figure 2:**
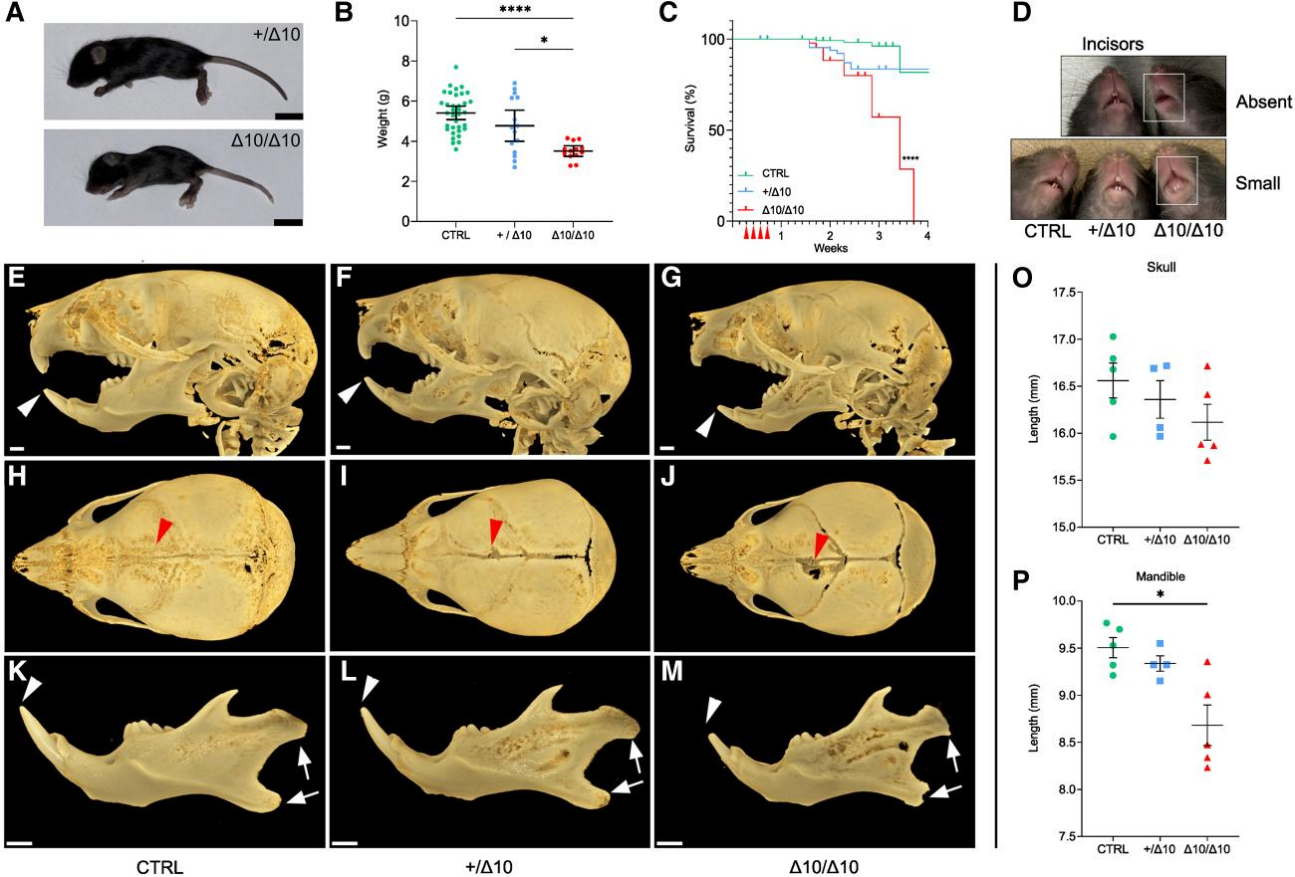
**Δ10 homozygous mice have reduced size and survival, as well as abnormal craniofacial development.** (**A**) Representative size difference between +/Δ10 (*top*) and Δ10/Δ10 (*bottom*) littermates (Scale bar = 1 cm). (**B**) Weight of transgenic mice at the P21 end point where each point represents an individual mouse weight, including *n* = 13 Δ10/Δ10, *n* = 15 +/Δ10 and *n* = 35 CTRL mice. (**C**) Kaplan-Meier plot of survival of Δ10/Δ10 (*n* = 43) versus +/Δ10 (*n* = 72) and CTRL (*n* = 116). Median survival in Δ10/Δ10 was 24 days. Comparison of the survival curves and statistical significance were evaluated by Log-rank (Mantel-Cox) test (*P* < 0.0001). (**D**) *Top:* representative image of littermates where the Δ10/Δ10 (*right*) has apparent absence of incisors at P21. *Bottom:* representative image of three littermates where the Δ10/Δ10 (*right*) has small incisors at P21. (**E**–**M**) MicroCT analysis of Δ10/Δ10 mice at P18 demonstrated dental and craniofacial anomalies. (**E**–**G**) Lateral views demonstrate hypoplastic incisors (white arrowheads) and mandibles in Δ10/Δ10. (**H**–**J**) Dorsal views show deficiencies in the frontal bone (red arrowheads) in Δ10/Δ10 mice relative to +/Δ10 and CTRL. The skull length tends to be shorter in homozygous mice relative to controls (see **O**, below), but the differences were not statistically significant. (**K**–**M**) Isolated mandibles demonstrate general hypoplasia and an abnormal condylar head and mandibular angle shape (white arrows). (**O** and **P**) The incisors (white arrowheads) are small, and the length of the mandible is significantly shorter in homozygous mutants relative to controls. (**O**) Quantification of skull length, with biological replicates plotted as individual points. (**P**) Quantification of mandible length, with biological replicates plotted as individual points. The *P*-values in the plots in **O** and **P** were determined using Brown-Forsythe and Welch one-way ANOVA followed by multiple comparisons testing using the Dunnett T3 method with α = 0.05. Scale bar = 1 mm. *P < 0.05, **P < 0.01, ***P < 0.001, ****P < 0.0001. POLR3B-HLD = RNA polymerase III subunit B-related hypomyelinating leukodystrophy; Δ10/Δ10 = tamoxifen-treated transgenic mice carrying a *Pdgfrα-CreERT* allele and homozygous *Polr3b^fl^* alleles; +/Δ10 = tamoxifen-treated transgenic mice carrying a *Pdgfrα-CreERT* allele and heterozygous *Polr3b^fl^* alleles; CTRL = tamoxifen-treated transgenic mice carrying no *Pdgfrα-CreERT* allele(s).

In Δ10/Δ10 mice, phenotypic differences from +/Δ10 and CTRL mice became apparent starting at P11/12. Behaviourally, Δ10/Δ10 mice displayed a neurological phenotype including ataxia, tremor and spontaneous seizures (Supplementary Videos 1–4). Δ10/Δ10 size differences also became apparent in the second week of life as they remained smaller than littermates. Δ10/Δ10 mice had significantly reduced body size and weight at the P21 end point, which underscored their abnormal developmental trajectory in the second and third weeks of life (Fig. 2A and B). The median survival of Δ10/Δ10 mice was 24 days, and all perished by 26 days (*n* = 43), whereas +/Δ10 (*n* = 72) and CTRL (*n* = 116) mice had adequate and comparable survival (Fig. 2C). Approximately 15% of +/Δ10 and CTRL mice were lost to the gestalt effects of tamoxifen and/or maternal neglect from significant perinatal handling (Fig. 2C). More detailed behavioural analysis and quantification were not done due to the small size, poor health and early lethality in Δ10/Δ10 mice.

Δ10/Δ10 mice also had craniofacial and dental anomalies, consistent with POLR3-HLD but unique among leukodystrophy mouse models (Fig. 2D). All Δ10/Δ10 mice had absent incisors or delayed eruption of underdeveloped incisors, whereas +/Δ10 and CTRL had normal tooth eruption/development (Table 1). High resolution microCT of the craniofacial bones and teeth (Fig. 2E–M) confirmed that Δ10/Δ10 mice had smaller/hypoplastic incisors with/without eruption (Fig. 2E–G) whereas molars appeared normal. Some +/Δ10 and all Δ10/Δ10 exhibited posterior frontal (metopic) suture anomalies, suggestive of increased pressure from constrained growth of the midface (Fig. 2H–J). Δ10/Δ10 had rounder appearing heads and their mandibular condyle and angle were overtly dysmorphic (Fig. 2H–M). Δ10/Δ10 mice tended to have smaller skulls (Fig. 2H–J) and mandibles (Fig. 2K–M) compared to CTRL, but differences between Δ10/Δ10 and +/Δ10 were not statistically significant (Fig. 2O and P). These apparent size differences were proportional to body weight/size.

**Table 1:** Hypodontia frequency in the *Polr3b*-HLD mouse model

	Incisor size at end point (*n*)			
*Polr3b* status	Normal	Small	Absent	Total
Δ10/Δ10	**0** *	**6** *	**9** *	**15**
+/Δ10	22	0	0	22
CTRL	41	0	0	41

The appearance of the incisors was categorized and counted at terminal end points (P14–P34) in 14 litters of Δ10/Δ10, +/Δ10 and CTRL mice. Frequencies in each incisor size group are categorized by genotype. Normal incisors were defined as incisors that were the same length as CTRL littermates. Small incisors were defined as being less than half the length of the incisors of a CTRL littermate. Absent incisors were categorized when incisors had not visibly erupted. Male and female Δ10/Δ10 mice had similar frequencies of absent incisors (5/8 females, 4/7 males) and small incisors (3/8 females, 3/7 males). In a sex-pooled statistical analysis, Δ10/Δ10 mice had a significantly smaller proportion of mice with normal incisors and greater proportion of mice with either small or absent incisors, as compared to +/Δ10s and CTRLs. Statistical analysis of incisor size and sex did not indicate dependence of these variables (not shown). Statistical significance was computed by cross-tabulation of genotype and incisor size using a Fisher-Freeman-Halton Exact test in SPSS v28.0.0.1 (IBM). The Fisher-Freeman-Halton Exact Test indicated a statistically significant association between genotype and incisor size (64.239, exact two-sided significance <0.001). The proportion of mice in an incisor size group were categorized by genotype using the Z-test of independent proportions with significance tested *post hoc* adjusting for multiple comparisons by the Bonferroni method (α = 0.05). Using this method, differences in the proportion of Δ10/Δ10s with normal, small, or absent incisors were evaluated as statistically different from the corresponding proportion in +/Δ10 and CTRL mice. POLR3B-HLD = RNA polymerase III subunit B-related hypomyelinating leukodystrophy; Δ10/Δ10 = tamoxifen-treated transgenic mice carrying a *Pdgfrα-CreERT* allele and homozygous *Polr3b*^fl^ alleles; +/Δ10 = tamoxifen-treated transgenic mice carrying a *Pdgfrα-CreERT* allele and heterozygous *Polr3b*^fl^ alleles; CTRL = tamoxifen-treated transgenic mice carrying no *Pdgfrα*-*CreERT* allele(s).

^*^ *P* < 0.05.

### Myelin basic protein immunofluorescence revealed hypomyelination in Δ10 homozygotes

**Figure 3:**
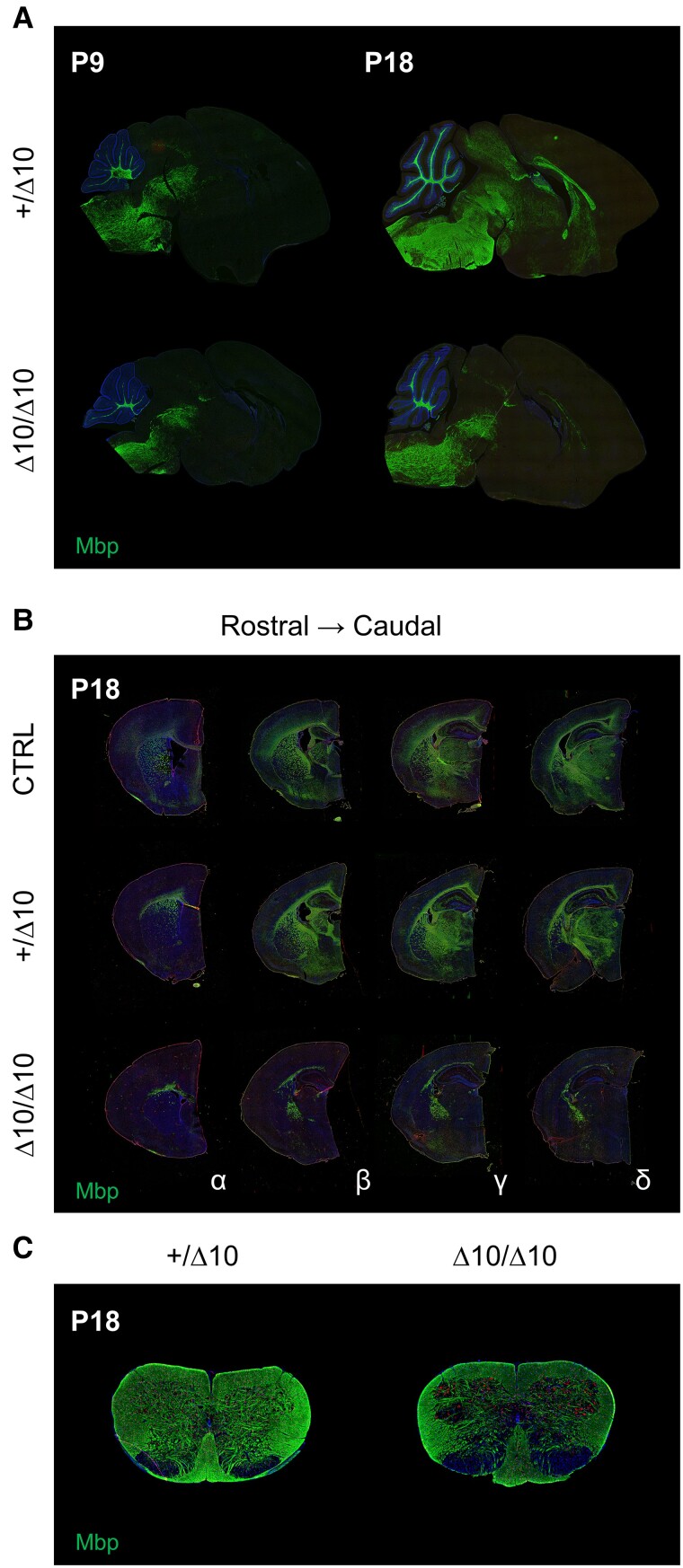
**Midbrain and forebrain myelinogenesis is interrupted between P9 and P18 in Δ10 homozygous mice.** (**A**) Mbp expression (green) in parasagittal sections from +/Δ10 (*top*) and Δ10/Δ10 (*bottom*) demonstrate interruption of myelinogenesis in the midbrain and forebrain between P9 and P18 in Δ10/Δ10. (**B**) Mbp expression (green) in hemicoronal sections from CTRL, +/Δ10 (*left*) and Δ10/Δ10 at P18. Four levels of sections (alpha, beta, gamma, delta) were cut from hemi-coronal blocks in order to assess Mbp expression via immunofluorescence. These four levels of sections correspond to the quantification found in Supplementary Fig. 3B. (**C**) Mbp expression in the cervical spinal cord at P18 demonstrates a relative reduction in ventral cord myelination between +/Δ10 (*left*) and Δ10/Δ10 (*right*). Reduced grey matter Mbp expression is also apparent in Δ10/Δ10. POLR3B-HLD = RNA polymerase III subunit B-related hypomyelinating leukodystrophy; Δ10/Δ10 = tamoxifen-treated transgenic mice carrying a *Pdgfrα-CreERT* allele and homozygous *Polr3b^fl^* alleles; +/Δ10 = tamoxifen-treated transgenic mice carrying a *Pdgfrα-CreERT* allele and heterozygous *Polr3b^fl^* alleles; CTRL = tamoxifen-treated transgenic mice carrying no *Pdgfrα-CreERT* allele(s); Mbp = myelin basic protein; Olig2 = oligodendrocyte transcription factor 2.

To understand whether Δ10 mice have a deficit in myelin development, we examined brain tissue for myelin basic protein (Mbp). On macroscopic inspection, Δ10/Δ10 brains had less visible ventral myelin, but overall tissue morphology was normal based on haematoxylin and eosin staining (Supplementary Fig. 2). However, immunofluorescence staining of fixed-frozen tissue collected at P9, P14 and P18 in CTRL, +/Δ10, and Δ10/Δ10 mice indicated significant hypomyelination. In P9 sagittal sections, Mbp expression was slightly reduced in Δ10/Δ10s, but the overall state of myelination appeared similar between +/Δ10s and Δ10/Δ10s (Fig. 3A). By P14, Mbp expression had developed in the midbrain and forebrain of +/Δ10s and CTRLs whereas in Δ10/Δ10s, development had not progressed. By P18, midbrain and forebrain Mbp expression were further increased in +/Δ10s/CTRLs, whereas expression had not advanced significantly in Δ10/Δ10s (Fig. 3A). We noted development of sparse Mbp expression in the corpus callosum and striatum in Δ10/Δ10s (Fig. 3B). In the cervical spinal cord, Mbp expression was also impacted, primarily in the ventral horn (Fig. 3C). Taken together, we observed an abnormal developmental progression of myelin antigen expression in the second and third weeks of life in Δ10/Δ10s (Fig. 3 and Supplementary Fig. 3).

### Quantifying hypomyelination severity in Δ10 homozygotes

**Figure 4:**
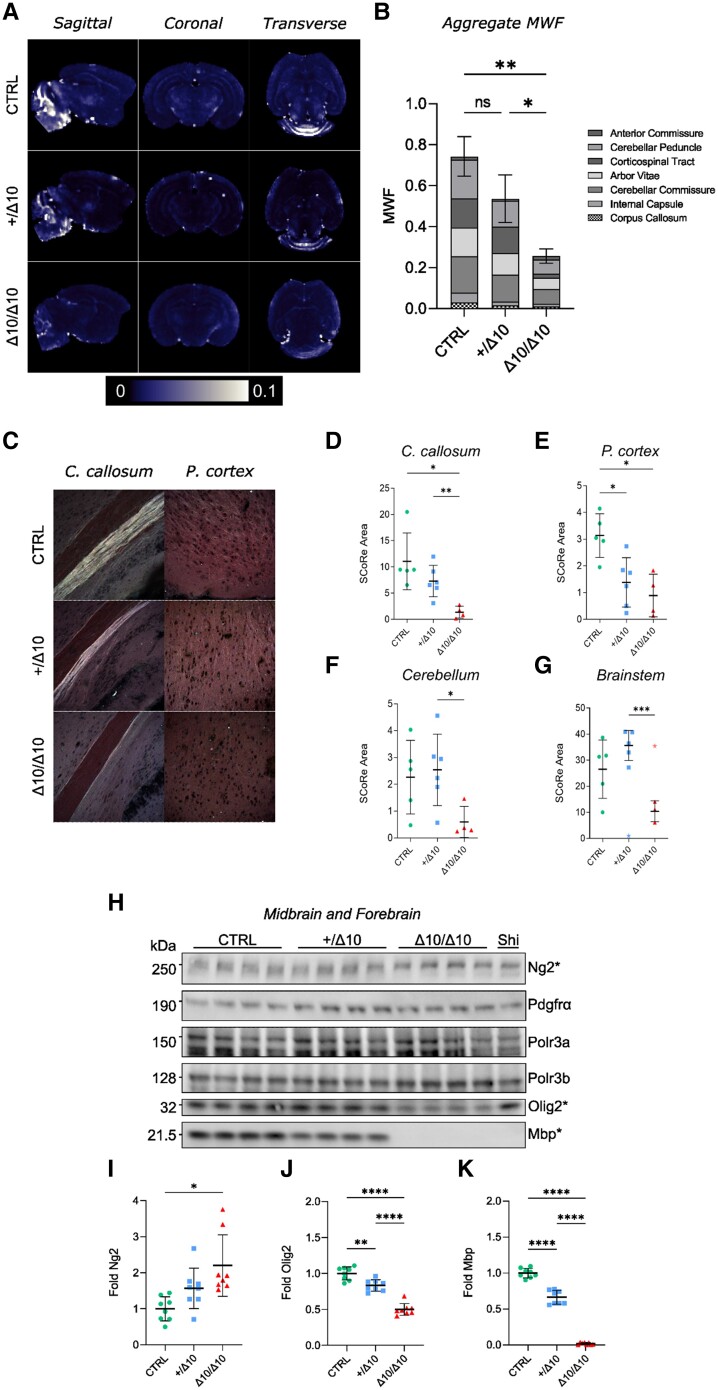
**Quantifying hypomyelination in Δ10 homozygous mice.** Hypomyelination was quantified using *ex vivo* MRI (**A** and **B**), spectral confocal reflectance (SCoRe) microscopy (**C**–**G**), and western blot at peak myelination in mice (**H**–**K**). (**A**) Myelin water fraction (MWF) maps from Δ10/Δ10, +/Δ10 and CTRL mice in sagittal, coronal and transverse views. (**B**) Summed aggregate MWF values from each region of interest (ROI) in Δ10/Δ10, +/Δ10 and CTRL mice (anterior commissure, cerebellar peduncle, corticospinal tract, arbor vitae, cerebellar commissure, internal capsule, corpus callosum). Error bars reflect standard deviation (SD). Statistical comparison of the Δ10/Δ10 and +/Δ10 was evaluated in the context of a Brown-Forsythe and Welch one-way ANOVA including Δ10/Δ10, +/Δ10, CTRL and tamoxifen-naïve mice. Details can be found in the Supplementary material. (**C**) Spectral confocal reflectance (SCoRe) microscopy was used to assess label-free sections from Δ10/Δ10 (*n* = 4), +/Δ10 (*n* = 6) and CTRL (*n* = 5) littermates for myelin content at P18. Representative images of the corpus callosum and superficial prefrontal cortex are shown. We quantified myelination as SCoRe area and compared Δ10/Δ10 to +/Δ10 in (**D**) corpus callosum [−81.2%; Dunnett's T3: μ_/Δ10_ = 1.374; μ_htz_ = 7.304; *t* = 4.388; df = 6.843; μ_Δ_ = 5.930; 95% confidence interval (CI_95_): 1.801–10.06; *P* = 0.0089], (**E**) cortex (−35.6%; Dunnett's T3: μ_/Δ10_ = 0.8910; μ_htz_ = 1.383; *t* = 0.8958; df = 7.269; μ_Δ_ = 0.4920; CI_95_: −1.186–2.170; *P* = 0.7562), (**F**) cerebellum (−76.5%; Dunnett's T3: μ_/Δ10_ = 0.5988; μ_htz_ = 1.945; *t* = 3.157; df = 7.267; μ_Δ_ = 1.945; CI_95_: 0.06251–3.827; *P* = 0.0435) and (**G**) brainstem (−70.8%; Dunnett's T3: μ_/Δ10_ = 10.41; μ_htz_ = 35.65; *t* = 7.288; df = 5.659; μ_Δ_ = 25.24; CI_95_: 14.19–36.30; *P* = 0.0009). Mean and standard error are shown. (**H**) Representative western blots from 15 µg brain lysate samples prepared at P21 where the medulla, pons and cerebellum were removed during dissection. There were no significant differences in Pdgfrα, Polr3a or Polr3b protein expression between genotypes. (**I**) Ng2 expression tended to be higher in Δ10/Δ10s than CTRLs but was not differentiated from +/Δ10s. When comparing Δ10/Δ10s to +/Δ10s, (**J**) Olig2 was reduced by 40.2% (Dunnett's T3: μ_/Δ10_ = 0.4990; μ_htz_ = 0.8338; *t* = 8.217; df = 14.00; μ_Δ_ = 0.3348; CI_95_: 0.2252–0.4445; *P* < 0.0001) and (**K**) Mbp was reduced by 97.3% (Dunnett's T3: μ_/Δ10_ = 0.01785; μ_htz_ = 0.6634; *t* = 18.83; df = 7.313; μ_Δ_ = 0.6455; CI_95_: 0.5408–0.7502; *P* < 0.0001). Four male and four female mice (*n* = 8) of each group listed were used in all western blot studies. Mean and standard deviation are indicated. Except for the MRI quantification detailed above, all statistics were computed using Brown-Forsythe and Welch's one-way ANOVA with multiple comparisons tested using Dunnett's T3 method and α = 0.05. *P < 0.05, **P < 0.01, ***P < 0.001, ****P < 0.0001. Δ10/Δ10 = tamoxifen-treated transgenic mice carrying a *Pdgfrα-CreERT* allele and homozygous *Polr3b^fl^* alleles; +/Δ10 = tamoxifen-treated transgenic mice carrying a *Pdgfrα-CreERT* allele and heterozygous *Polr3b^fl^* alleles; CTRL = tamoxifen-treated transgenic mice carrying no *Pdgfrα-CreERT* allele(s); Shi = Shiverer; C. callosum = corpus callosum; P. cortex = prefrontal cortex; Ng2 = Neuron-glial antigen 2 (encoded by *Cspg4*); Pdgfrα = Platelet-derived growth factor receptor alpha.

We performed detailed studies to quantify the putative hypomyelination in our transgenic model at P18, including MRI. Assessment of the MWF using *ex vivo* MRI techniques on PFA-fixed brains from P18 mice revealed visible differences in MWF maps indicative of significant hypomyelination in Δ10/Δ10s, in addition to a substantial effect of tamoxifen on myelination overall (Supplementary Fig. 4A–G). Overall myelination status at the group level was described in aggregate by taking the summed average MWF values from seven ROIs (Supplementary Fig. 4F and G). Representative MWF maps are shown in Fig. 4A. Quantitative comparison of +/Δ10 and Δ10/Δ10 aggregate MWF revealed a statistically significant decrease in myelination in Δ10/Δ10s relative to +/Δ10s (Fig. 4B).

To better understand the degree of hypomyelination, we pursued both spectral confocal reflectance (SCoRe) microscopy (Fig. 4C), a label-free technique for imaging myelin^54^ and traditional myelin staining with Luxol Fast Blue (LFB) (Supplementary Fig. 5). At P18, both methods demonstrated reduced myelination in Δ10/Δ10s compared to littermates. More specifically, statistically significant reductions in mean SCoRe area were observed in the corpus callosum (Fig. 4D), cerebellum (Fig. 4F) and brainstem (Fig. 4G) of Δ10/Δ10s relative to +/Δ10s. Both MRI and histological techniques agreed with our Mbp expression studies that hypomyelination was well established by P18.

Finally, we confirmed the amount of protein expression in the midbrain/forebrain by performing western blots using midbrain/forebrain lysates from male and female mice at P21 (Fig. 4H).^55^ Our assays included Polr3a, Polr3b, Pdgfrα, Ng2, Olig2 and Mbp. Among these, Ng2 was on trend for increased expression in Δ10/Δ10s, while Olig2 and Mbp were negatively correlated with Δ10 allele dose (Fig. 4I–K). We observed statistically significant reductions in both Olig2 (Fig. 4J) and Mbp (Fig. 4K) expression in Δ10/Δ10s relative to +/Δ10s. We ran age-matched homozygous shiverer tissue lysates as a negative control next to samples from Δ10/Δ10 mice and found the Mbp western blot quantification results to be indistinguishable. Our tissue biochemistry results quantified significant hypomyelination in the midbrain/forebrain of Δ10/Δ10 mice.

### Δ10 homozygotes produce fewer oligodendrocyte precursors, reducing the number of myelinating cells

**Figure 5:**
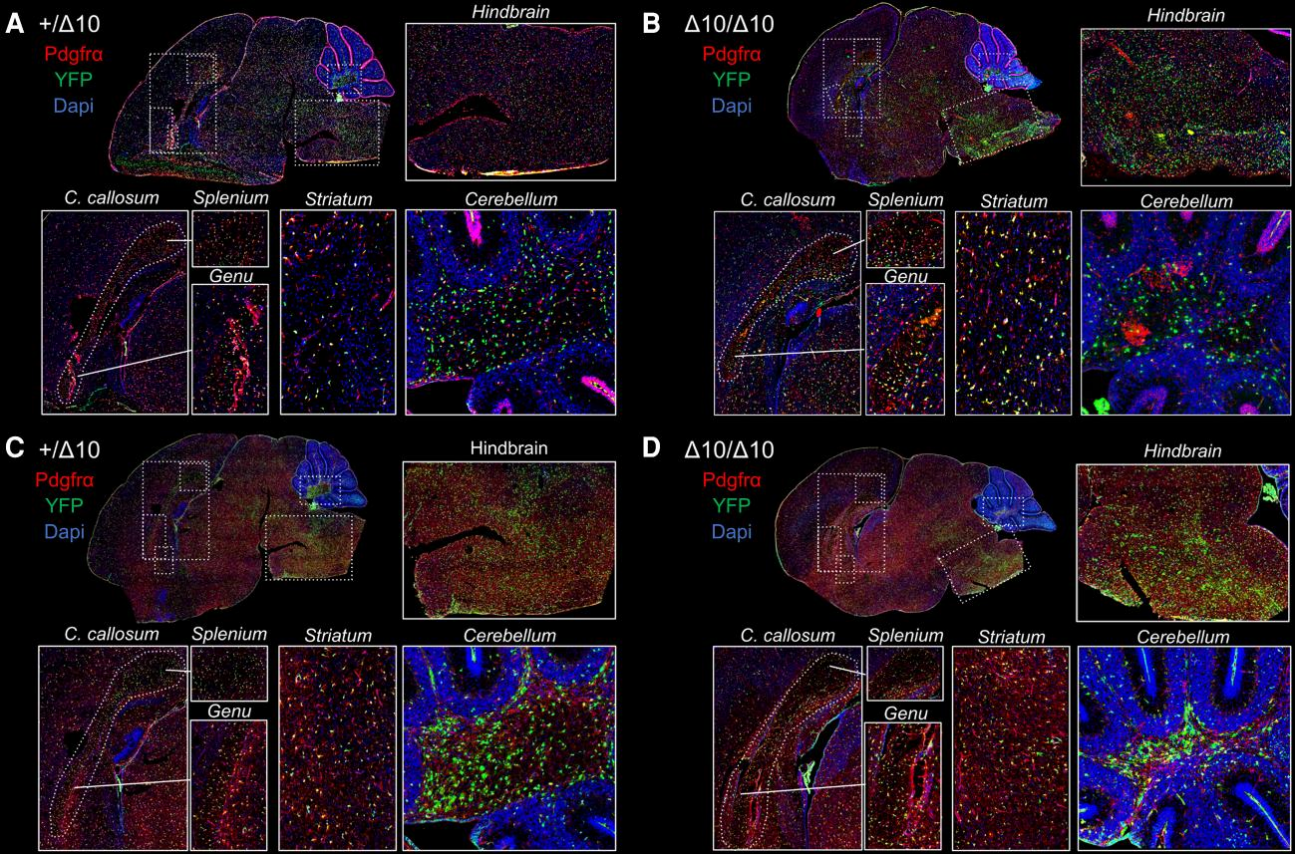
**Oligodendrocyte subpopulations appear stable at P9 in Δ10 homozygous mice.** We probed for Pdgfrα (**A** and **B**) and Ng2 (**C** and **D**) protein expression in YFP+ cells at P9 in +/Δ10s (**A** and **C**) and Δ10/Δ10s (**B** and **D**). Cells co-expressing YFP and Pdgfrα or Ng2 appeared to be slightly more numerous in Δ10 heterozygotes relative to homozygotes. *Inset* pictures display hindbrain (pontomedullary), corpus callosum (splenium and genu), striatum and cerebellar arbor vitae. Δ10/Δ10 = tamoxifen-treated transgenic mice carrying a *Pdgfrα-CreERT* allele and homozygous *Polr3b^fl^* alleles; +/Δ10 = tamoxifen-treated transgenic mice carrying a *Pdgfrα-CreERT* allele and heterozygous *Polr3b^fl^* alleles; CTRL = tamoxifen-treated transgenic mice carrying no *Pdgfrα-CreERT* allele(s); C. callosum = corpus callosum; YFP = yellow fluorescent protein; Pdgfrα = platelet-derived growth factor receptor alpha.

To ascertain whether there were differences in subpopulations of oligodendrocyte lineage cells (OLCs) that might explain the observed hypomyelination, we conducted YFP+ lineage tracing on histological sections using antibodies against lineage marker antigens. We quantified cells based on the expression of markers of OPCs (Pdgfrα+ and/or Ng2+) or mature oligodendrocytes (OL; Apc clone CC1+). We investigated hindbrain, midbrain and forebrain structures at P9, P14 and P18 (Supplementary Figs 6 and 7). At P9, YFP+Pdgfrα+ OPCs were present throughout parasagittal brain sections (Fig. 5A and B), as were YFP+Ng2+ OPCs (Fig. 5C and D), and the total numbers of CC1+ OLs were equivalent between groups, making it an ideal time point to begin to study OLC population dynamics leading up to the period of peak myelination in mice.

**Figure 6:**
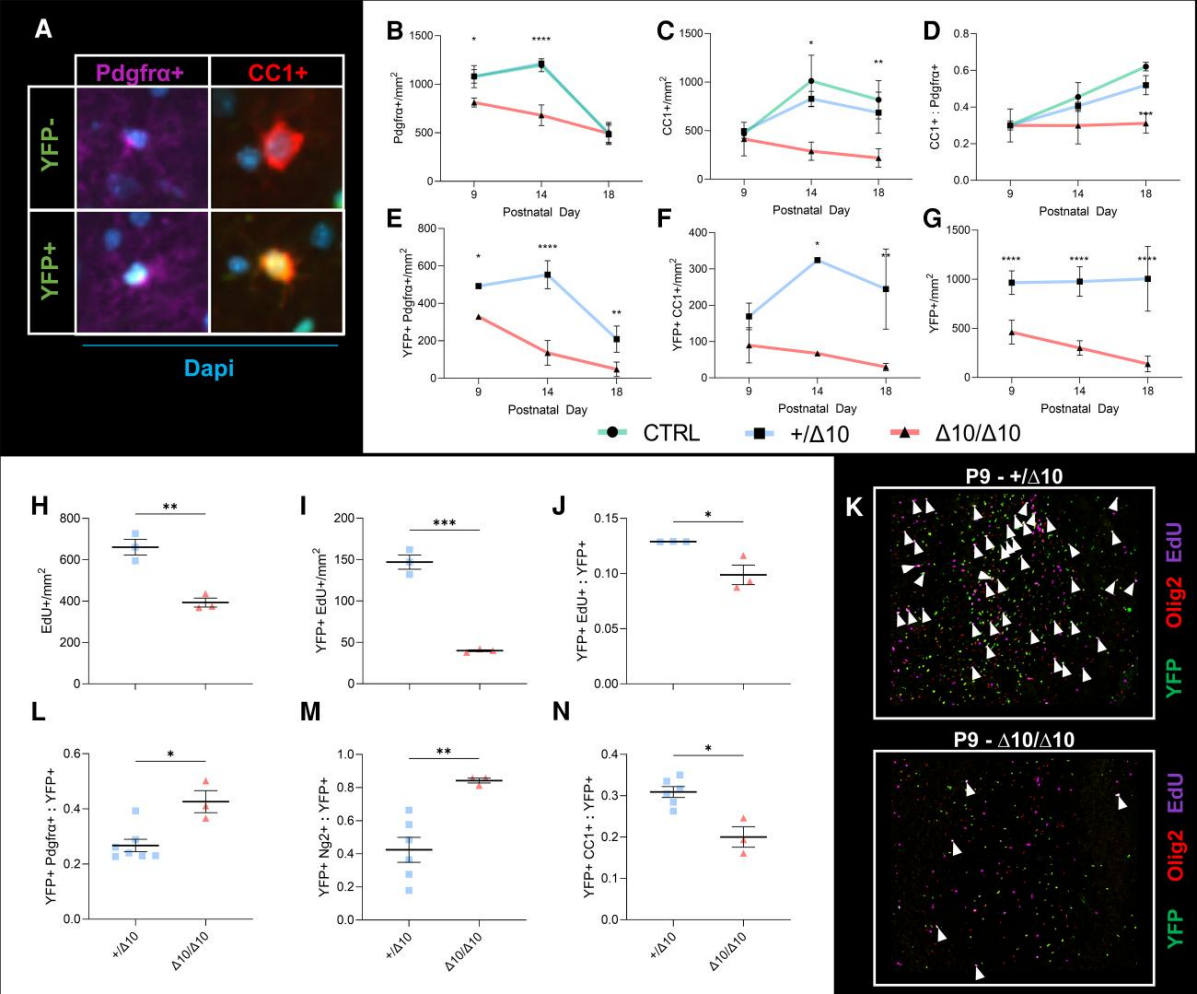
**In Δ10 homozygous mice, hypomyelination is caused by decreased numbers of myelinating oligodendrocytes due to proliferation and maturation defects**. (**A**) Counting yellow fluorescent protein (YFP) positive or negative Pdgfrα+ OPCs and CC1+ mature oligodendrocytes (OLs) in sagittal sections from Δ10/Δ10s, +/Δ10s, and CTRLs allowed us to track the maturation of the oligodendrocyte lineage in transgenic mice. At P9, P14 and P18, counting single-positive (**B**) Pdgfrα precursor or (**C**) CC1 mature oligodendrocyte lineage cells (OLCs) reveals that while CTRLs (green lines) and +/Δ10s (blue lines) have similar population dynamics, Δ10/Δ10s (red lines) fail to increase numbers of precursor and mature OLCs throughout the brain. (**D**) The maturation of the overall population is demonstrated as an increasing ratio of CC1+ mature OLCs to Pdgfrα+ precursors in CTRL and +/Δ10 mice, but this does not occur in Δ10/Δ10s. (**E**) YFP+Pdgfrα+ OPC dynamics largely mirror the scale and direction of changes in the single positive Pdgfrα population. (**F**) YFP+CC1+ OLC dynamics largely mirror the scale and direction of changes in the single positive CC1 population. (**G**) YFP+ cell numbers decline throughout the observation window, suggesting that while adequate numbers of OLCs are not produced in Δ10/Δ10s, existing cells are also depleted from the parenchyma over time. Mean of biological replicates and standard deviations are shown for the plots in **B**–**G**, animal numbers and biological replicates for each time series are detailed in Supplementary Table 3. Statistics for graphs in **B**–**G** were computed using two-way ANOVA with multiple comparisons (between genotypes at each time point) tested using the Tukey method and α = 0.05. (**H**–**J**) Proliferation at P9 as assessed by EdU incorporation after a 6 h labelling protocol is reduced in Δ10/Δ10s compared to +/Δ10s in terms of (**H**) number of proliferating cells, (**I**) number of YFP+ proliferating cells, and (**J**) the relative proportion of proliferating cells among YFP+ cells. (**L**–**N**) At P18, a higher proportion of OLCs in Δ10/Δ10s are immature compared to +/Δ10s. (**L**) A higher proportion of YFP+ cells co-express Pdgfrα. (**M**) A higher proportion of YFP+ cells co-express Ng2. (**N**) At P18, Δ10/Δ10s have a lower proportion of mature OLCs compared to +/Δ10s. A lower proportion of YFP+ cells co-express CC1. (**K** ) Cells triple labelled for YFP, Olig2 and EdU (white arrowheads) are significantly more numerous in +/Δ10s (upper panel) than in Δ10/Δ10s (lower panel). Statistics for graphs in **H**–**N** were computed using unpaired, two-tailed *t*-tests with Welch's correction and α = 0.05. *P < 0.05, **P < 0.01, ***P < 0.001, ****P < 0.0001. Δ10/Δ10 = tamoxifen-treated transgenic mice carrying a *Pdgfrα-CreERT* allele and homozygous *Polr3b^fl^* alleles; +/Δ10 = tamoxifen-treated transgenic mice carrying a *Pdgfrα-CreERT* allele and heterozygous *Polr3b^fl^* alleles; CTRL = tamoxifen-treated transgenic mice carrying no *Pdgfrα-CreERT* allele(s); SEM= standard error of the mean; Pdgfrα = platelet-derived growth factor receptor alpha; CC1 = clone CC1 of anti-adenomatous polyposis coli (also: Anti-Quaking 7); Ng2 = Neuron-glial antigen 2 (encoded by *Cspg4*); EdU = 5-ethynyl-2′-deoxyuridine.

Cell counting indicated steadily reduced numbers of OPCs and OLs over the P9–P18 window in Δ10/Δ10s (Fig. 6). We analysed both single positive cells and cells co-expressing markers in addition to YFP. Single positive Pdgfrα+ or CC1 cells and cells co-expressing these markers in addition to YFP, showed similar population dynamics in +/Δ10s and CTRLs suggesting that tissue-level OLC population dynamics were relatively normal in +/Δ10s. By comparison, in Δ10/Δ10s, OLC populations decreased in number over time (Fig. 6B and C). +/Δ10s and CTRLs demonstrated an increase in the ratio of OLs (CC1+) to OPCs (Pdgfrα+) indicating maturation in the overall OLC population leading up to peak of myelination. While both populations were decreasing in number over time, the ratio of OLs to OPCs remained stable in Δ10/Δ10s suggesting that the remaining population did not undergo aggregate maturation like the other genotypes (Fig. 6D). Finally, the scale and direction of changes in the number of YFP+ co-expressors were similar to those of their respective single-positive populations (Fig. 6E–G). Taken together, Δ10/Δ10s demonstrated a significant difference in their ability to produce a large number of OPCs and OLs in the second postnatal week and failed to accumulate adequate numbers of myelinating OLCs.

### Defective proliferation and maturation drive oligodendrocyte population dynamics in Δ10 homozygotes

Given that the overall YFP+ population in Δ10/Δ10s was already ∼50% smaller than that of +/Δ10s at P9, we hypothesized that the reduced OLC numbers were the result of decreased OPC proliferation, which is typically maximal during the first postnatal week.^56–58^ EdU injections at P9 using a 6-hour exposure protocol to assess the percentage of dividing cells demonstrated decreased EdU+ labelling in Δ10/Δ10s compared to +/Δ10s, as well as a decrease in the ratio of YFP+EdU+ to total YFP+ cell population. These studies indicated that the loss of proliferative potential occurred both in terms of the absolute number of dividing cells and the relative proportion of dividing cells among the YFP+ population (Fig. 6H–J). These studies explained the altered OLC population dynamics in Δ10/Δ10s and confirmed that defective proliferation among OPCs immediately precedes hypomyelination in the model.

**Figure 7:**
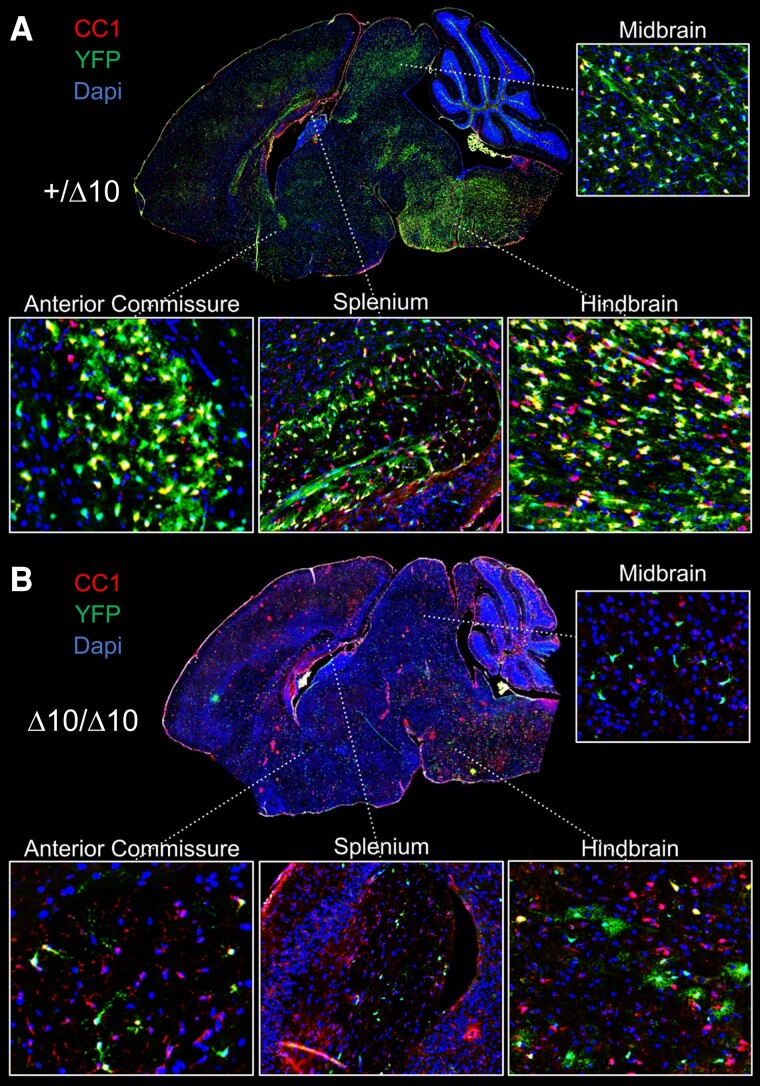
**Depletion of YFP+ cells from the parenchyma in Δ10 homozygous mice**. Comparison of +/Δ10 (**A**) and Δ10/Δ10 (**B**) demonstrated widespread depletion of yellow fluorescent protein positive (YFP+) cells in the parenchyma of Δ10/Δ10. Representative images shown. Among remaining YFP+ cells, Δ10/Δ10 had cells with shorter processes and more immature morphology. *Inset* pictures show the hindbrain, midbrain, splenium of the corpus callosum, and anterior commissure. Δ10/Δ10 = tamoxifen-treated transgenic mice carrying a *Pdgfrα-CreERT* allele and homozygous *Polr3b^fl^* alleles; +/Δ10 = tamoxifen-treated transgenic mice carrying a *Pdgfrα-CreERT* allele and heterozygous *Polr3b^fl^* alleles; CTRL = tamoxifen-treated transgenic mice carrying no *Pdgfrα-CreERT* allele(s); CC1 = clone CC1 of anti-adenomatous polyposis coli (also: Anti-Quaking 7).

Since our lineage tracing studies indicated early proliferation deficits in the OPC population, and because the YFP+ population failed to undergo aggregate maturation, we were also interested in the fate of OLCs in Δ10/Δ10s. Indeed, at P18 we did observe relative increases in the proportion of YFP+Pdgfrα+ and YFP+Ng2+ precursors to the total YFP+ population, as well as a relative decrease in the proportion of YFP+CC1+ mature cells to the total YFP+ population in Δ10/Δ10s (Fig. 6K–M). Therefore, we considered whether stalled proliferation and maturation in OPCs led to apoptosis in a specific subpopulation of YFP+ cells. We stained P9, P14 and P18 sections for Cleaved Caspase 3, a marker of apoptosis.^25,59^ These studies demonstrated little Caspase 3 activation in all brain sections examined, suggesting that apoptosis is not the main cause of the reduced number of OLCs (Supplementary Fig. 8A and B). Representative images of a +/Δ10 and Δ10/Δ10 are illustrative of the difference in YFP+ cell numbers at P18 (Fig. 7A and B), suggesting another mechanism of cell loss is at play.

Taken together, we observed specific OLC population dynamics in Δ10/Δ10s that allowed us to pinpoint both the stage and timing of the defect underlying hypomyelination in the model. Early OPC proliferation was limited, reducing the number of cells with myelinating potential. Fewer OPCs matured and became myelinating cells, resulting in an increased proportion of immature OLCs in Δ10/Δ10s. Finally, OLCs were progressively lost from the parenchyma, due to an unidentified mechanism. The increased proportion of Pdgfrα+ and/or Ng2+ OLCs among remaining YFP+ cells at P18 suggest that these OLCs did not regularly progress past the precursor stage, or that the YFP+ cells that did progress past this stage were less fit. In summary, an early defect in proliferation and subsequent maturation among OPCs leads to reduced numbers of myelinating cells and causes hypomyelination in the Δ10 model of POLR3B-HLD.

## Discussion

Pol III assembly and nuclear import is a complex process, which is known to be perturbed in POLR3-HLD, including in association with mutations in *POLR3B.*^17^ Yee *et al.*^24^ assayed the transcriptional function of the mutant complex in zebrafish expressing the *Slj* mutation constitutively, demonstrating that transcription of pre-tRNA and *7sl* Pol III targets were reduced by more than 50% in mutant larvae, while *5Ss* levels remained relatively stable. They indirectly demonstrated that *Slj* impacted Rpc2–Rpc11 (Polr3b-Polr3k) interactions, proposing that the mutation affected Pol III facilitated recycling activity by reducing re-initiation, but did not publish experimental confirmation of the mechanism. Our *in silico* modelling of Δ10 on the human Pol III structure also indicated the likely interaction of POLR3B exon 10 with POLR3K in the jaw region of the human polymerase. Our proteomics data suggested that POLR3BΔ10 caused a severe Pol III complex assembly defect, as well as reduced expression of the mutant protein in the cytoplasm and nucleus of human cells.

Based on our proteomics data, we concluded that the main mechanism of Pol III hypofunction resulting from Δ10 expression is a likely Pol III complex assembly defect, although we could not categorically rule out complex instability. Indeed, we directly demonstrated that Δ10 has reduced association with Pol III subunits as compared to wild-type protein by AP-MS. However, we also observed that mutant protein was less highly expressed than wild-type at the cytoplasmic and nuclear level when expressed transiently under identical conditions in HEK293T cells. Because Pol III is assembled in the cytoplasm and then imported into the nucleus,^60^ testing for nuclear expression indirectly supported that Δ10 does not cause total failure of Pol III complex assembly, consistent with its prior characterization as a hypomorph.^24,25^ Taken together, we confirmed that Δ10 perturbs the structural integrity of Pol III.

Based on several of our findings, we hypothesize that reduced expression of Δ10 is related to a process downstream of Pol III complex assembly. Although transcript instability is possible, because we detected similar expression of the orthologous Δ10 and wild-type mRNA up to 30 days after induction with tamoxifen when validating brain recombination in mouse studies, it is unlikely to be a significant factor *in vivo*. Moreover, we did not detect multiple splice products in transgenic mice, suggesting that the mutant exon 9–11 junction is a stable product, and that nonsense-mediated decay is unlikely. Finally, *in silico* analysis does not suggest that free (unassembled) human Δ10 has reduced half-life compared to its wild-type protein counterpart.^61^ In summary, evidence of transcript instability was not observed *in vivo* using a highly similar orthologous mutant, while the free mutant protein is not expected to have reduced half-life. We therefore propose that reduced expression of the mutant detected in our human *in vitro* assays is due to turnover of subunits that fail to be assembled into functional enzymes. In summary, we have elucidated that Δ10 impairs Pol III complex assembly or stability, thereby disrupting its physiological functions upstream of transcription. On the basis of these findings, we elected to study Δ10 in the context of oligodendrocyte development and myelinogenesis.

The mutation employed in our study explores an alternative molecular mechanism in line with prior characterization of disease-causing variants including a *POLR3B* variant that causes POLR3-HLD and impairs Pol III assembly or stability.^3,17^ Indeed, recent attempts to recapitulate POLR3-HLD *in vivo* have largely been unsuccessful with the exception of a recent study by Merheb *et al*.,^18^ where a constitutively lethal W671R/G672E double mutation in *Polr3a* was used in the context of a Cre/Lox strategy to elicit a myelin phenotype. The orthologous mutation used in Merheb *et al.*^18^ has ∼30% of the transcriptional activity of WT Pol III in *Saccharomyces cerevisiae*, but complex assembly was not assayed directly. Complex assembly is not known to be significantly affected in the single G672E mutant,^16^ and the orthologous Y685K/G686E yeast mutant described in Moir *et al.*^19^ is not expected to affect subunit interactions. Taken together, there are now two mechanistic models of POLR3-HLD: a milder neurological phenotype based on a *Polr3a* double mutant that causes reduced Pol III transcriptional output,^18^ and our more severe model based on a *Polr3b* mutation that perturbs Pol III structural integrity.

We chose to use an inducible/conditional strategy to direct expression of Δ10 to OPCs using a *Pdgfrα-CreERT*-driven approach because it allowed us to study postnatal development, a critical period of myelinogenesis in the brain. Using an inducible/conditional approach to study myelination has several advantages, the most significant being that lethality is avoidable and the severity of the phenotype can be titrated. Because we elected to study the early oligodendrocyte lineage and OPCs are generated throughout development, we were faced with the reality that we would not be able to induce complete tissue recombination.^62,63^ Because of this, we elected to use a triple transgenic strategy in order to better understand the contributions of recombined and non-recombined cells to the overall CNS pathology. Using this method, we observed significant hypomyelination associated with YFP+ cell dysfunction, namely proliferation and maturation defects, despite the presence of YFP-, non-recombined cells. The reporter used in the study is known to be highly specific to recombined cells but is less sensitive and may underestimate the true recombination rate.^64^ This is consistent with our finding that CC1+ cell density decreased in Δ10/Δ10 mice to a slightly greater degree than would be explained by the decrease in YFP+CC1+ cells alone (compare Fig. 6C with Fig. 6F). Therefore, it is likely that the observed pathology was less affected by non-recombined cells than would be anticipated based on our YFP-based estimate of recombination rate. Altogether, we did not observe evidence that non-recombined cells had a material impact on our experimental outcomes, which showed remarkable correlation to key aspects of the human disease.

Recombination rates were not amenable to augmentation by administering tamoxifen earlier, or for longer periods. In early pilot studies we found that prenatal tamoxifen administration caused difficulties during parturition in line with prior studies.^65^ Furthermore, the doses of tamoxifen used in our study were at the upper limit of tolerability, precluding extended treatment in the context of a comparable dosing regimen. Finally, we observed reduced myelination in CTRL mice at tolerable doses as compared to tamoxifen-naïve mice, in contrast to *in vitro* and *in vivo* studies suggesting that tamoxifen increases remyelination in mice (Supplementary Fig. 4).^66,67^ This conflict in experimental observations may be due to either pharmacokinetic differences between the studies (dosing, neonatal versus adult pharmacokinetic differences), or differences in tamoxifen pharmacodynamics in the context of myelinogenesis versus remyelination. Our pilot studies that identified an effect of tamoxifen on myelination led us to design our experiments with equal tamoxifen exposure in all comparator groups.

Emergence of non-CNS anomalies such as hypodontia and craniofacial abnormalities are expected to be caused by *Pdgfrα* expression in non-CNS tissues. Indeed, although *Pdgfrα* is a high-fidelity marker of the early oligodendrocyte lineage in the mouse CNS, it is widely expressed along the developmental continuum in various neural crest derivatives throughout the body including the developing craniofacial bones and teeth.^68-70^ We observed an absence of incisors in approximately half of Δ10/Δ10s and small incisors among the rest. Given that many Δ10/Δ10s did not have eruption prior to death, we assume that eruption is not merely delayed but rather that incisors are significantly underdeveloped, most likely due to an underlying hypoplasia. The manifestation of hypomyelination, hypodontia and craniofacial abnormalities is interesting and suggests that further study of the functional similarities between neural crest-derived mesenchymal derivatives and oligodendrocytes may yield greater understanding of the mechanistic link between the disparate phenotypes observed in POLR3-HLD.

Altogether, we conducted an array of studies to confirm and determine the extent of hypomyelination in our transgenic mice. Several histological and biochemical measurements we performed suggested that +/Δ10s were not physiologically equivalent to CTRLs. POLR3-HLD is recessive, and heterozygotes/carriers are not known to have hypomyelination or associated clinical deficits. We attributed differences in results between CTRLs and +/Δ10s to the increased sensitivity afforded by studying the Δ10 mutation at different allelic doses on a genetically identical background. Moreover, the histological and biochemical studies performed here are also incredibly sensitive to changes in myelination relative to clinical MRI used in patient studies. Importantly, we did not observe behavioural or other obvious phenotypic differences between +/Δ10s and CTRLs. Therefore, we determined that characterizing the phenotype in Δ10/Δ10s by comparing to +/Δ10s was most apt, given that the only genetic difference between the two groups is the additional Δ10 allele. Unfortunately, we could not compare our findings in +/Δ10 mice to the prior published *Polr3a* model, because Merheb *et al.*^18^ limited their study to comparisons with wild-type mice. We found that +/Δ10 and CTRL mice have minor differences in oligodendrocyte physiology based on tissue-level measurements. Their similarity in terms of the observed myelination outcomes emphasizes that not all cell-level changes will cause defective myelination outcomes *in vivo* due to reserve capacity in the myelinating population. Moreover, this highlights the importance of *in vivo* studies for evaluating whether a particular mutation will produce hypomyelination.

Studies of myelination confirmed severe hypomyelination in Δ10/Δ10s. Notably, marked decreases in Mbp immunofluorescence were identified in sagittal and hemi-coronal sections (Supplementary Fig. 3). Hemi-coronal sections demonstrated a certain degree of spatial variability in the Mbp signal, which may reflect the contribution of cells that did not undergo cre-mediated recombination during tamoxifen exposure. However, we noted that the pattern of preserved myelination corresponded to spinal cord, subcortical areas corresponding to the posterior internal capsule and pallidum, and corpus callosum. Patients with POLR3-HLD are known to have preserved myelination in specific structures, including in the pallidum and corticospinal tracts at the level of the posterior limb of the internal capsule. Therefore, this variability may also be reflective of intrinsic differences in the response of these brain areas to Pol III hypofunction in mice, similar to the human disease. *Ex vivo* MRI and other histological studies (e.g., SCoRe imaging) focusing on quantification of myelin lipids agreed with the immunofluorescence results. Depending on the modality of inquiry and brain region, we detected anywhere from ∼50% to >80% loss of myelin at P18.

Histological results were validated by western blot using midbrain/forebrain tissue lysates at the peak of myelination (P21). Midbrain and forebrain Mbp expression was reduced by >97%, but the exact percentage cannot be ascertained due to a floor effect. Our panel of immunoblotting antibodies also revealed reduced Olig2 expression, which likely reflects reduced numbers of OLCs. Among the other targets tested, none suggested significant changes at the tissue level. As expected, we did not detect significantly reduced Polr3b expression in our *in vivo* western blots due to using samples in which mutant cells make up a small percentage of the dissected tissue. *Ex vivo* MRI and histological studies agreed with our studies of OLC protein expression, however our western blot data suggested a more severe decrease in Mbp as compared to the imaging studies focusing on regions of interest that are heavily myelinated. While it is expected to see more severe decreases on western blot since aggregate Mbp across both grey and white matter regions is measured, it is possible that myelin components may be lost to a more severe degree than the cells themselves due to dysfunction among remaining OLCs. In a recent study published by our lab, we observed that *Polr3b* knockdown was associated with reduced expression of markers of myelination in differentiating oligodendrocytes, including substantially reduced *Mbp* transcription.^71^ Because we also observed evidence of differentiation defects, it is likely that remaining CC1+ cells are dysfunctional, explaining the discrepancy in the scale of decreases in Mbp expression and loss of CC1+ cells. Taken together, the model recapitulated severe hypomyelination and produced effect sizes that will be useful for testing therapeutic interventions in the future.

Using lineage tracing methods, we tracked the fate of subpopulations of oligodendrocytes to ascertain the mechanism of hypomyelination. From the outset, we were specifically interested in the question of whether hypomyelination in POLR3-HLD is caused by a failure to obtain adequate numbers of myelinating cells or defective cell function leading to improper or reduced production of myelin and/or myelin components such as Mbp. Our finding that YFP+ cell numbers were already reduced by 50% relative to heterozygotes at P9 suggested a proliferation defect that preceded our observation window, consistent with maximal CNS Pdgfrα+ cell proliferation from late gestation through the first postnatal week.^56-58^ Indeed, we observed that the Δ10/Δ10 OPC population did not undergo a rapid increase in size from P9 to P14, and mature OLCs did not accumulate in the brain from P9 onward. Direct testing of proliferation with EdU labelling confirmed this hypothesis and demonstrated that a significant proliferation defect was the most upstream factor leading to hypomyelination in our model. Because we did not detect activated Caspase 3 at P9 (or afterward), we concluded that the abrogated proliferation and maturation of the OLC lineage in the early stages produced inadequate numbers of myelinating cells and was directly responsible for the observed hypomyelination.

Our model differs from that described by Merheb *et al*.^18^ in that we developed it based on mutation of *Polr3b*, and moreover using a mutation that causes defective Pol III biogenesis. Indeed, our model is more severe and uniquely produces CNS-dependent phenotypes common among hypo/dysmyelination mouse models, including ataxia, tremor, seizures and early death.^72-76^ We expect this to be the case because Pol III complex assembly is upstream from transcription, and disruption of upstream processes typically lead to more wide-ranging effects on cell physiology. Moreover, we detected and quantified a specific defect in proliferation, which occurred at a critical developmental time point, immediately prior to the peak of myelination. This is likely to play a major role in the development of hypomyelination in our model and is also expected to be an underlying factor in the difference in severity between our observations and those of Merheb *et al*.^18^

We detected steadily decreasing numbers of YFP+ cells in homozygous mice across all endpoints, which decreased in number throughout the observation window. The dwindling numbers of OLCs demonstrated in our model were also suggested by the findings of Merheb *et al*.^18^ Therefore, two animal models making use of mutations in distinct genes, causing Pol III dysfunction by different mechanisms, suggest that reduced myelinogenesis is related to reduced numbers of myelinating cells. Although we cannot yet say with certainty that this is true in POLR3-HLD, in the only existing study quantifying oligodendrocyte-specific immunohistochemistry in a patient, OLIG2+ cells were reduced in number by 70–99% when compared to a control, depending on the brain region.^11^ Therefore, our current understanding of POLR3-HLD pathophysiology indicates that putative interventions should be targeted to early stages of the oligodendrocyte lineage in order to produce therapeutic benefit, in line with recently published hypotheses by Perrier and colleagues.^77^

## Conclusions

In this study, we characterized the *POLR3B*Δ10 mutation in human cells and developed an animal model of POLR3-HLD using the orthologous mutation in mice. Our proteomics studies demonstrated that Δ10 impacts Pol III structural integrity, likely by impairing assembly of the complex. Postnatal expression of the Δ10 mutant early in the oligodendrocyte lineage using an inducible/conditional strategy in mice recapitulated recessive disease features such as hypomyelination, hypodontia and craniofacial abnormalities. In the CNS, homozygous Δ10 expression was associated with severe hypomyelination, which was caused by significant proliferation and maturation defects in OPCs. Taken together, we produced a unique and novel model of POLR3-HLD: the first of its kind to be based on *Polr3b* mutation and to recapitulate patients’ neurological and craniofacial/dental abnormalities, opening the door to further study disease mechanisms and test potential therapeutics.

## Supplementary Material

awad249_Supplementary_Data

## Data Availability

Original data underlying this manuscript that were generated at MyeliNeuroGene lab can be obtained from the corresponding author on reasonable request. Of note, a substantial portion of the original data are available as Supplementary material. Original craniofacial data generated at the University of Missouri, Kansas City, and the Stowers Institute for Medical Research, can be accessed from the Stowers Institute Original Data Repository at http://www.stowers.org/research/publications/lipbp-2400.
