# Supplementary material for: Hypomyelination, hypodontia and craniofacial abnormalities in a Polr3b mouse model of leukodystrophy
Source: Brain. 2023 Aug 28;146(12):5070–85. doi: 10.1093/brain/awad249 (PMC10690025; doi:10.1093/brain/awad249)

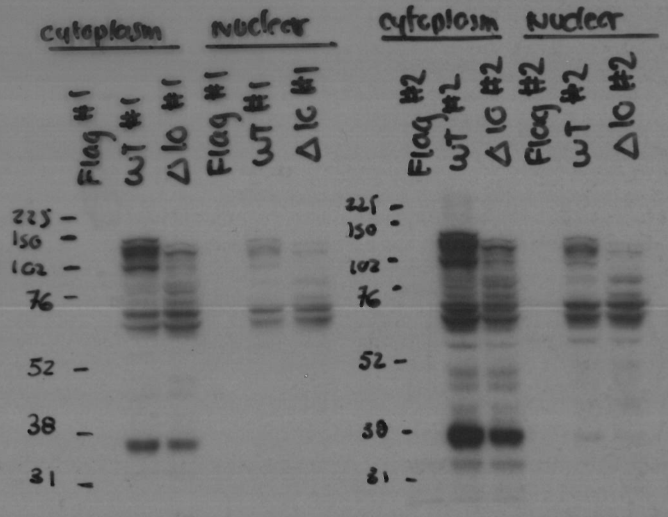

#1 and #2

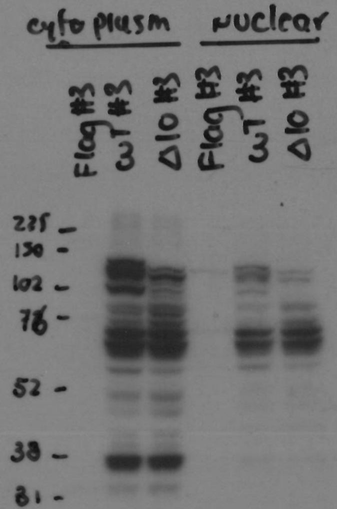

#3

maximo 2022/04/07 Exposition time 5sec. 10% SDS-PAGE
40µg was loaded for cyto and 26 of nuclear extract per lane
α·Mouse anti Flag (M2) (1:2000) and α·sheep anti mouse HRP (1:5000)

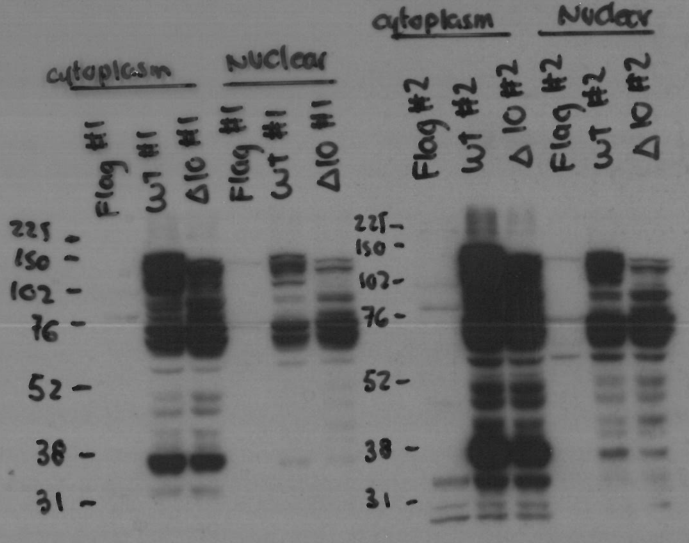

#1 et #2

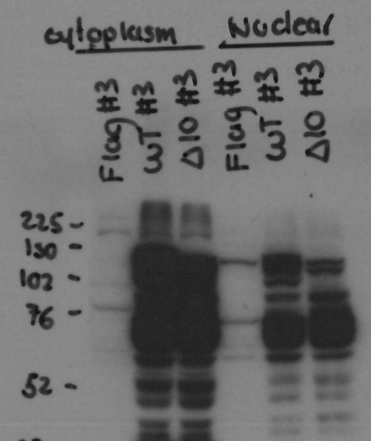

#3

maxime 2022/04/07 10% SDS PAGE Exposition time : 30 sec.
40μg of cytoplasm extract and 26μg of Nuclear extract in each appropriate lane
α. mouse anti Flag(M2) (1:2000) and α. sheep anti mouse HRP (1:5000)

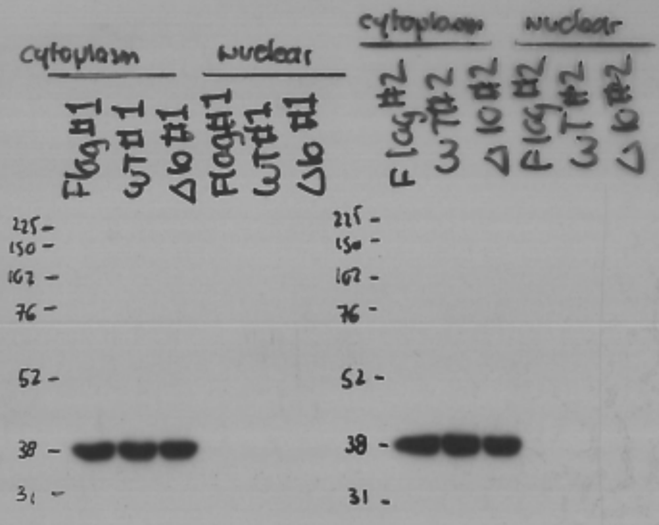

#1 and #2

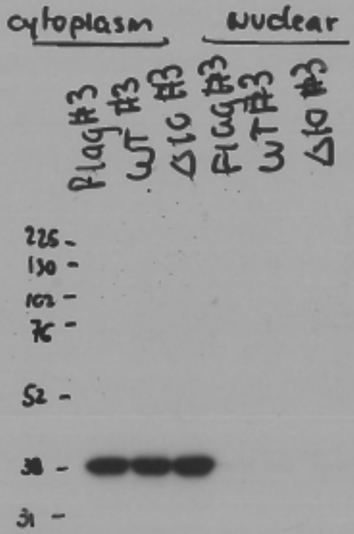

#3

Maxima 2022/04/19 10% SDS-PAGE Exposition time: 5seconds
40μg of cytoplasm extract and 26μg of nucleus extract was loade
in each appropriate lanes
α-mouse anti GAPDH (1: 1000) and α-sheep anti mouse HRP (1:500

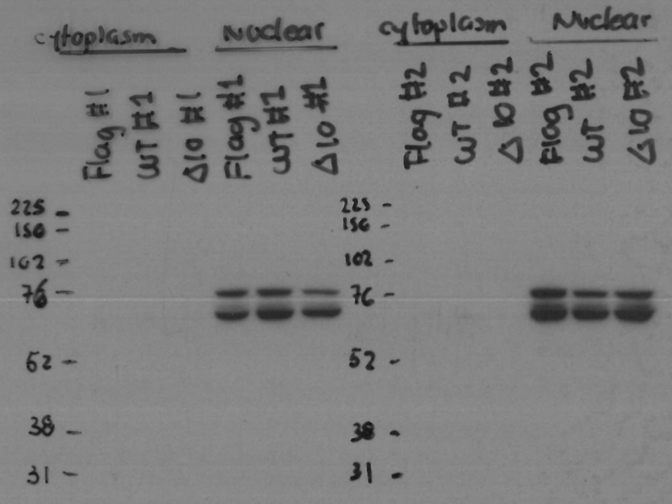

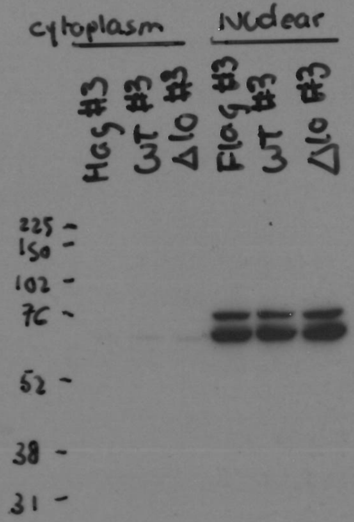

maxime 2022/04/12 10% SDS PAGE Exposition time 5 seconds.

40 μg of cytoplasmic extract and 26 μg of nuclear extract was loaded in each lane

α-rabbit anti lamin A+C (1:2000) and α sheep anti rabbit HRP (1:5000)

Males - Polr3a - mb.tif

2551 x 1902

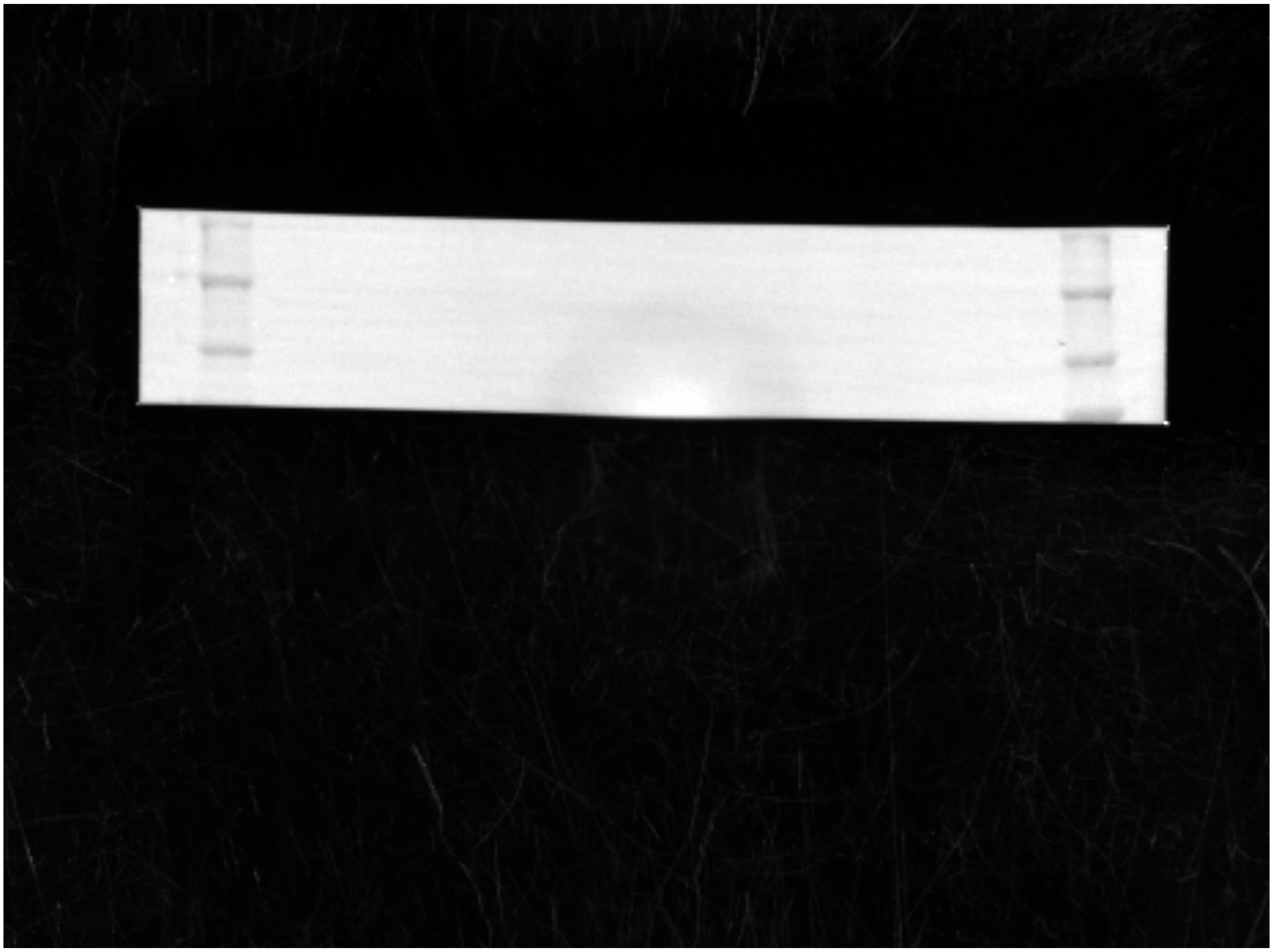

Males - Polr3a - pt.tif

2598 x 1941

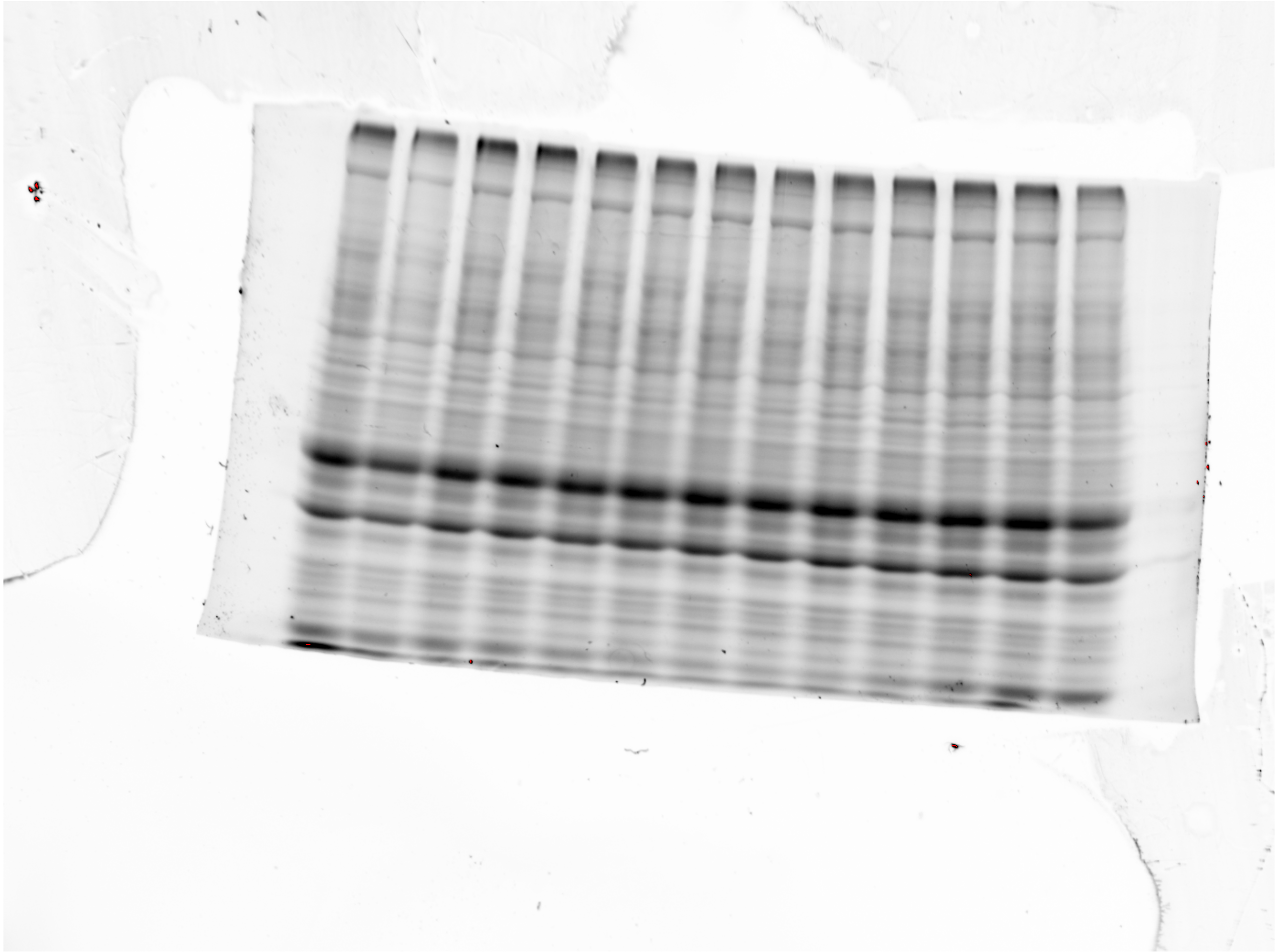

Males - Polr3a - tp.tif

2551 x 1906

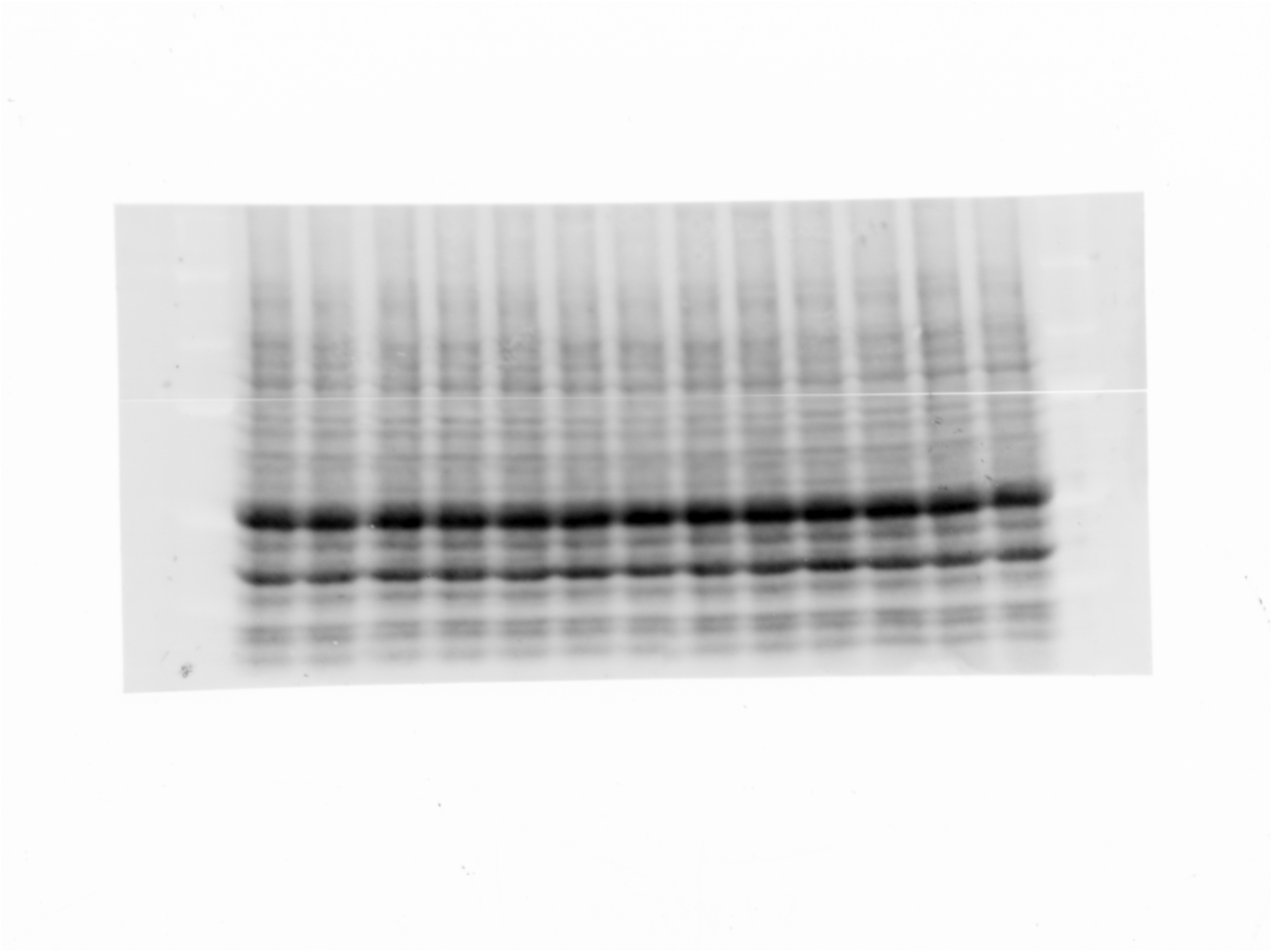

Males - Polr3a.tif

2551 x 1906

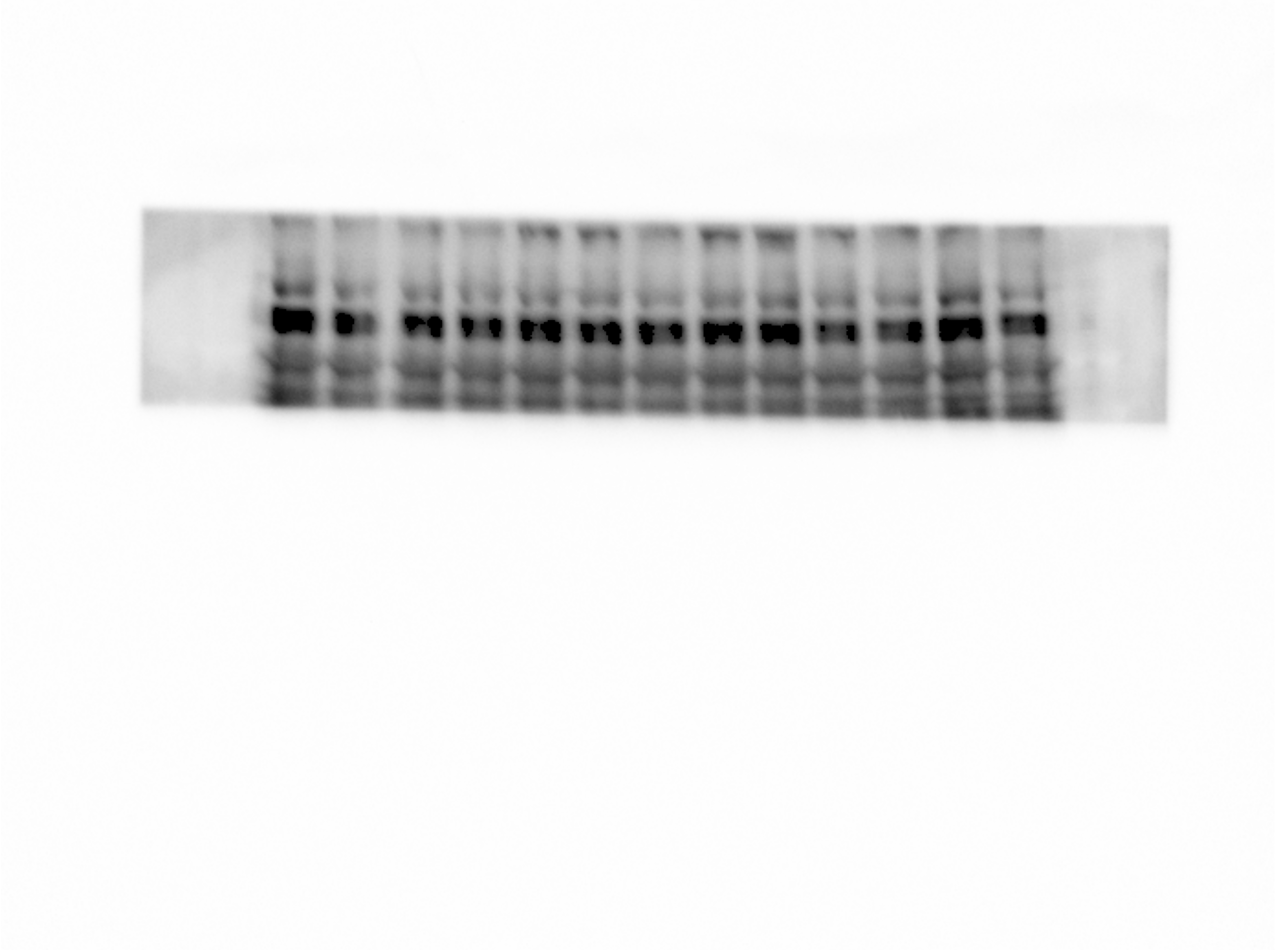

Males - Polr3b - mb.tif

2551 x 1902

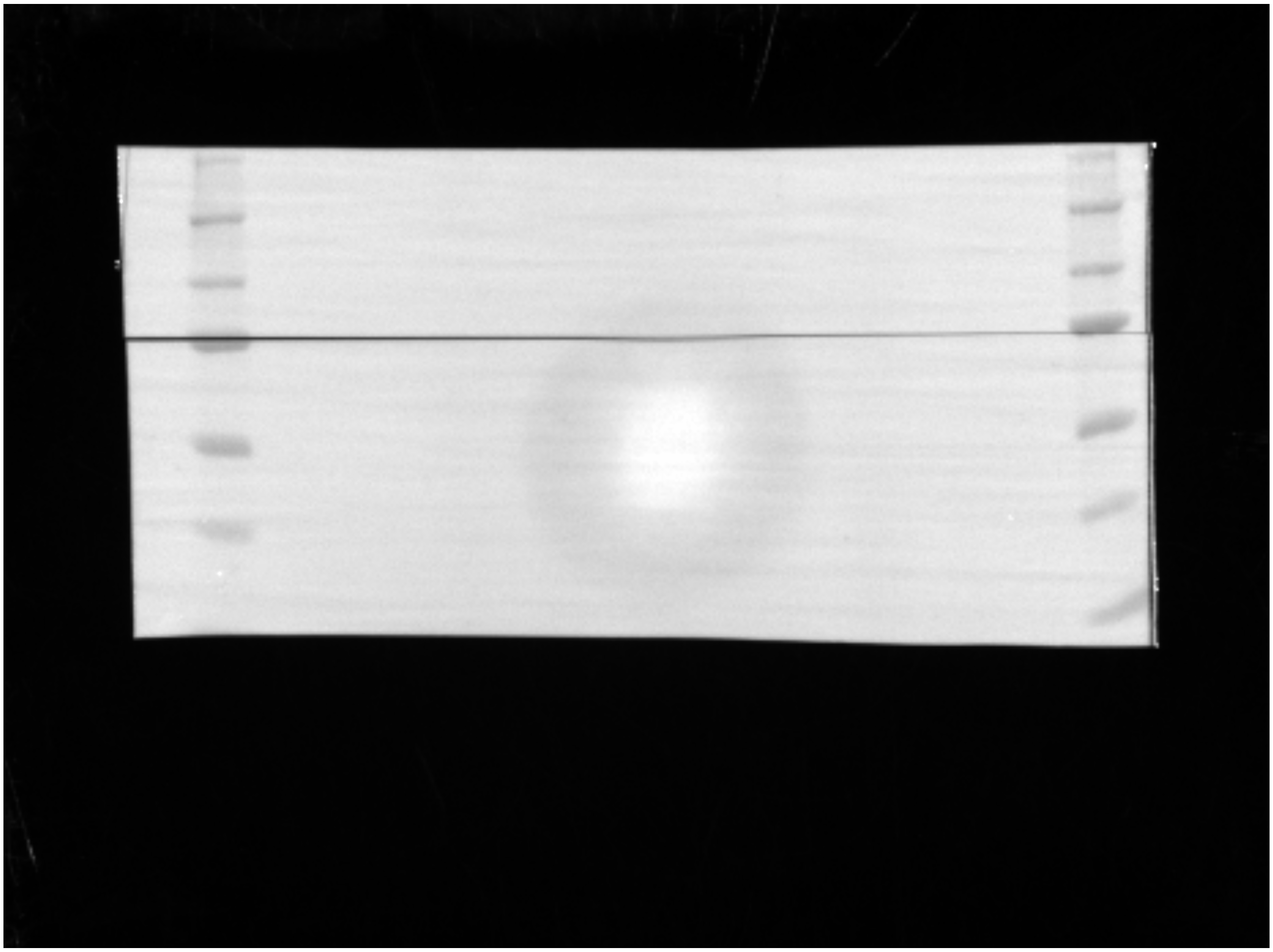

Males - Polr3b - pt.tif

2058 x 1136

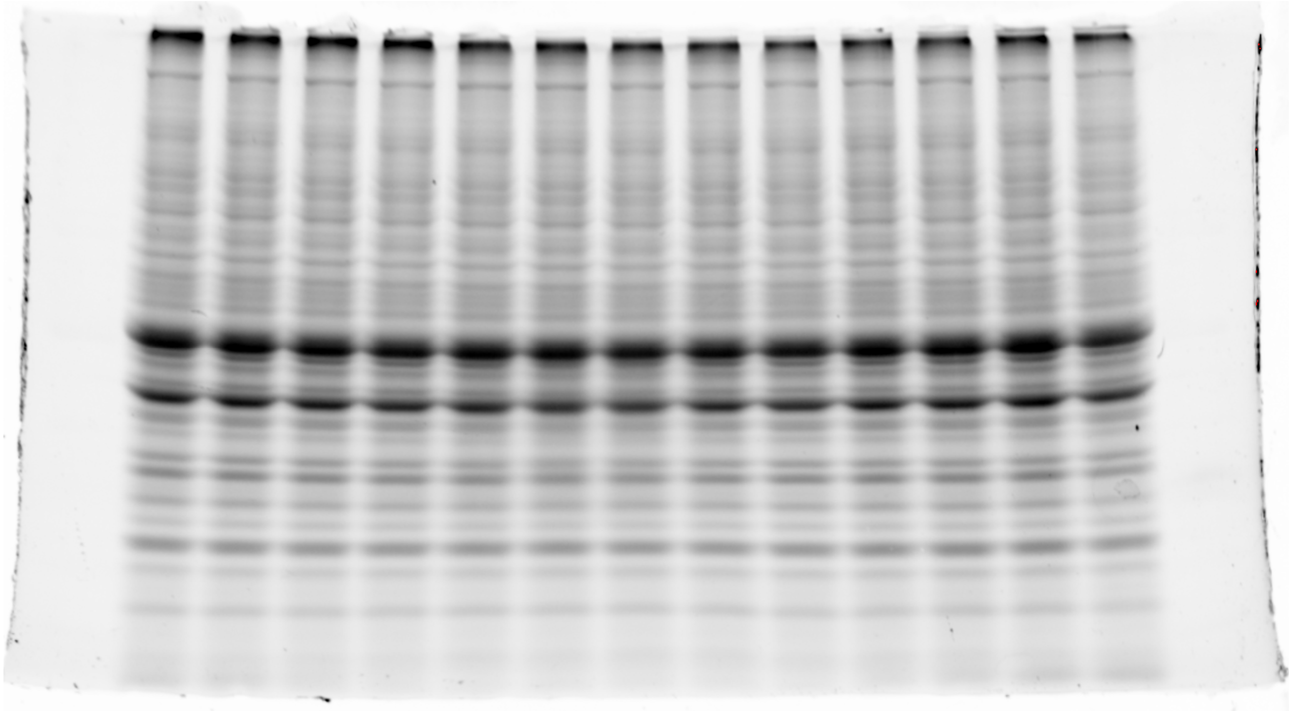

Males - Polr3b - tp.tif

2551 x 1906

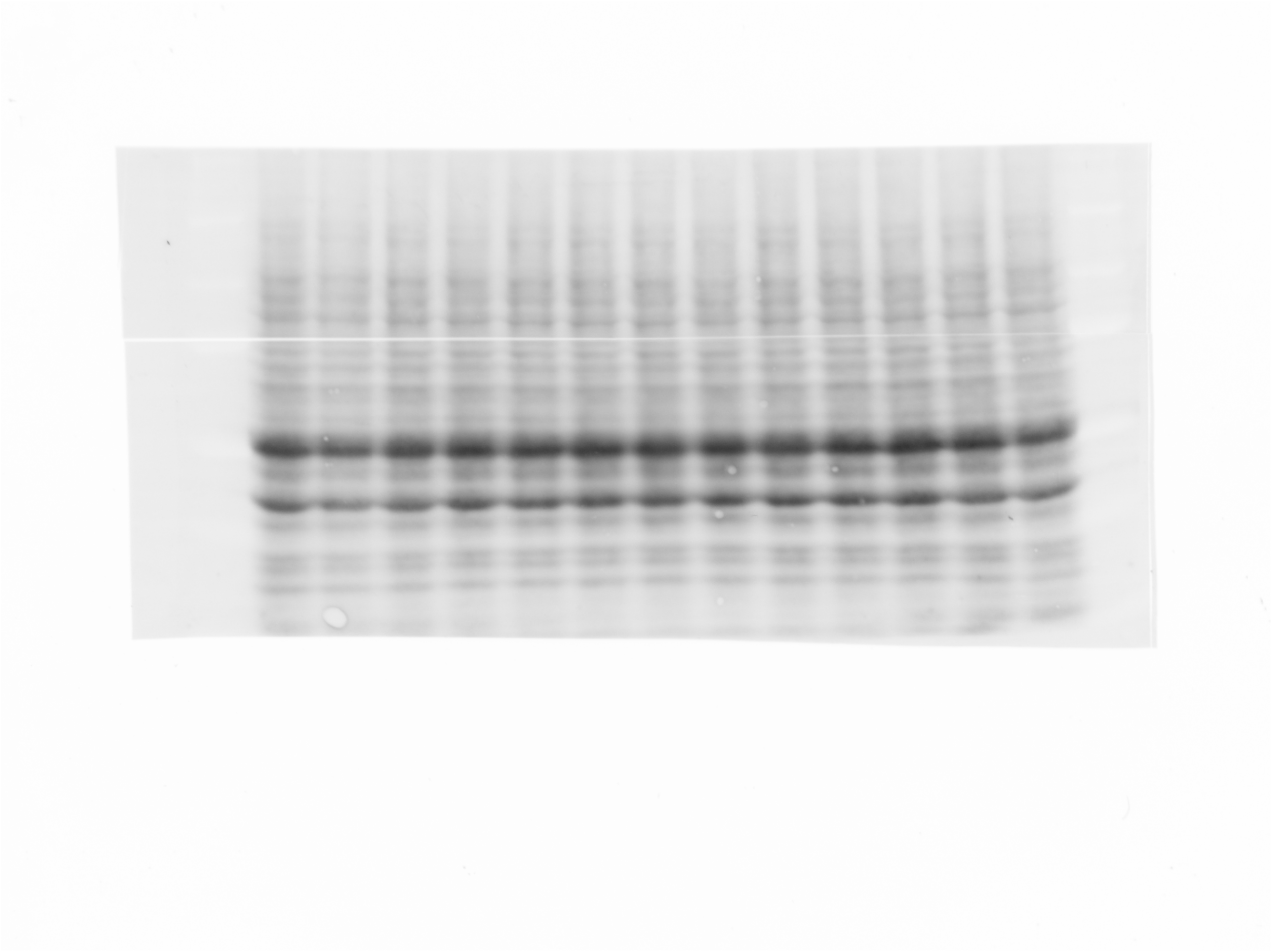

Males - Polr3b.tif

2551 x 1906

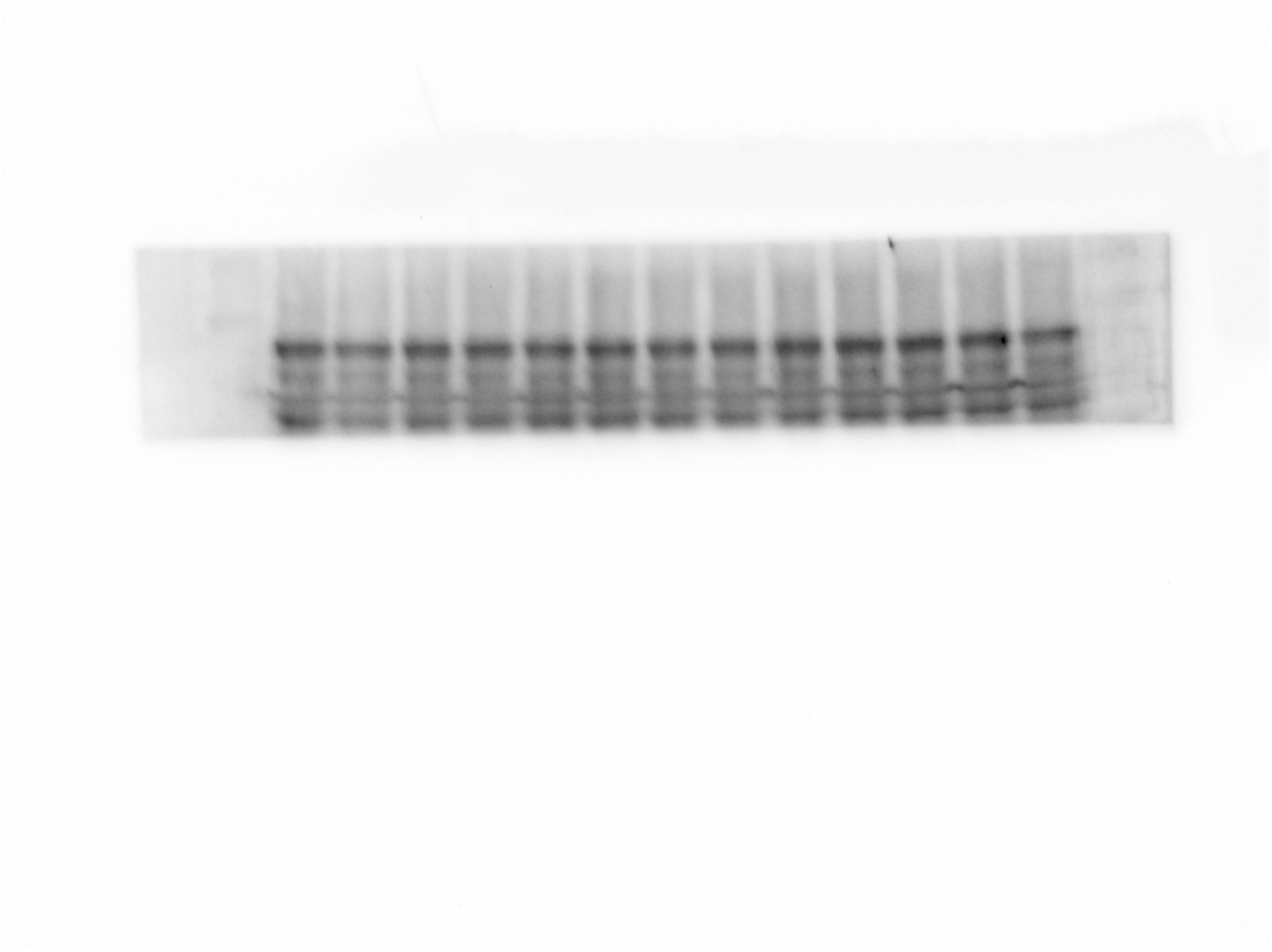

Females - Mbp - pt.tif

2480 x 1853

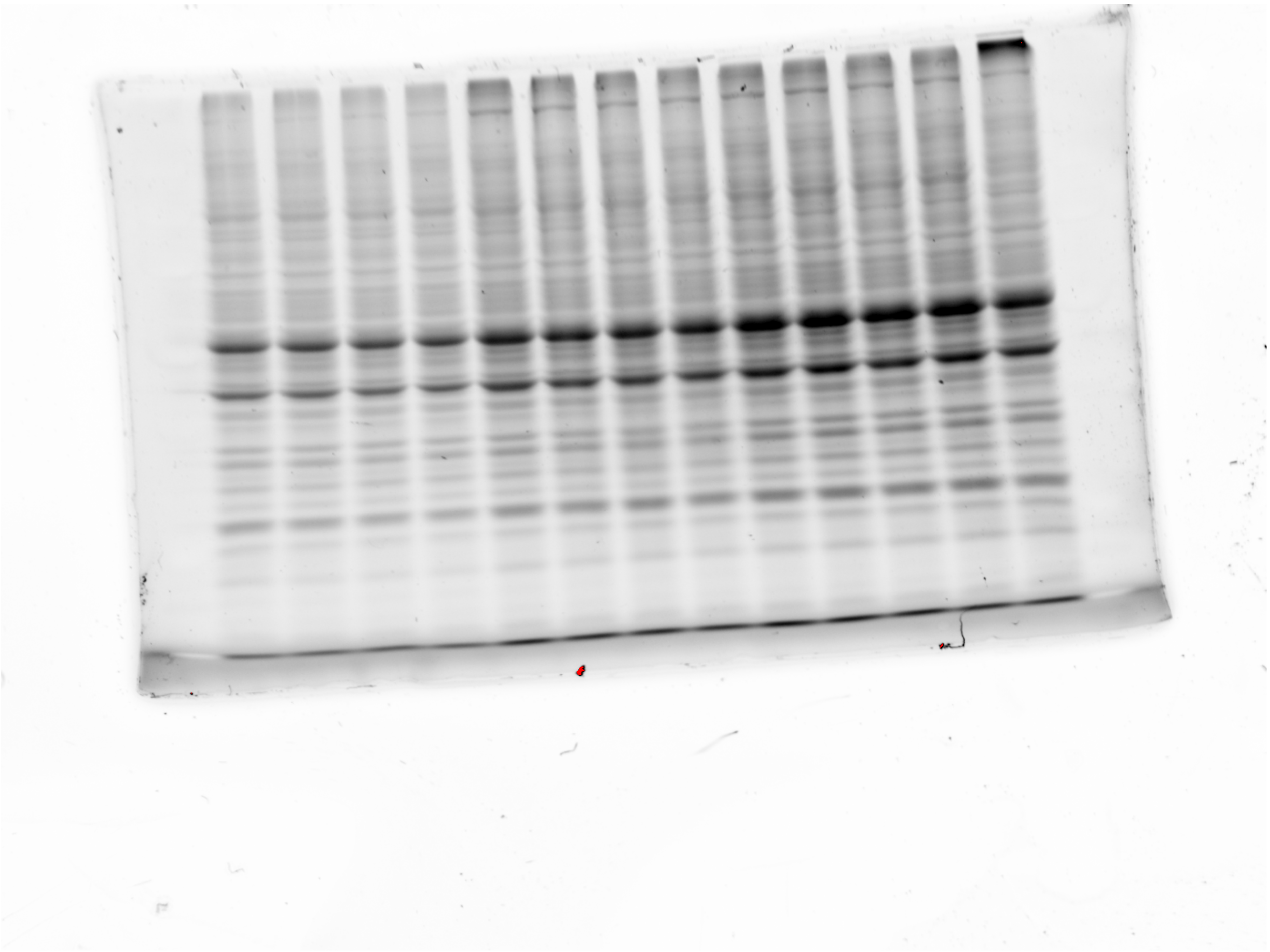

Females - Mbp - tp.tif

2244 x 1676

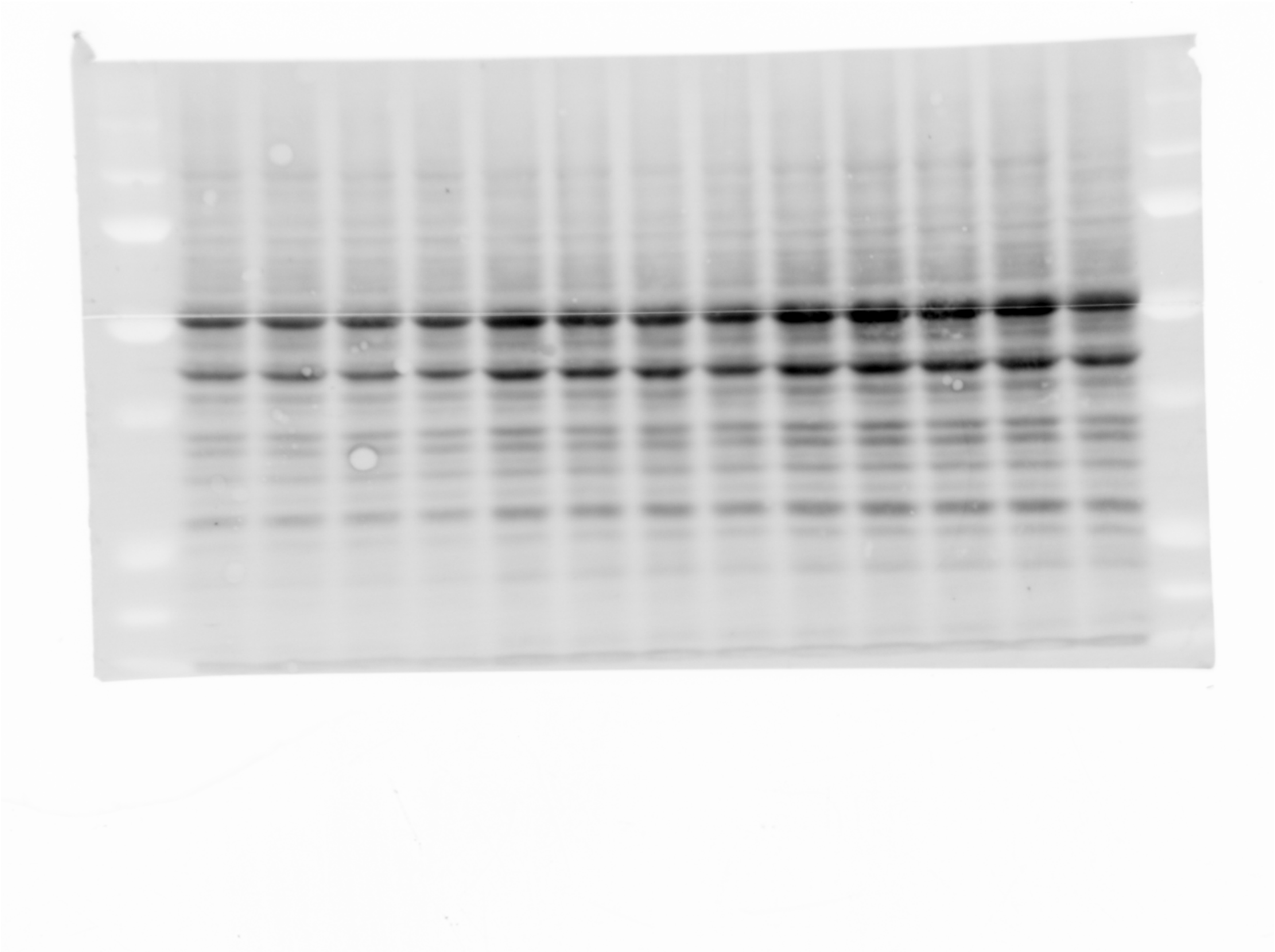

Females - Mbp.tif
2244 x 1676

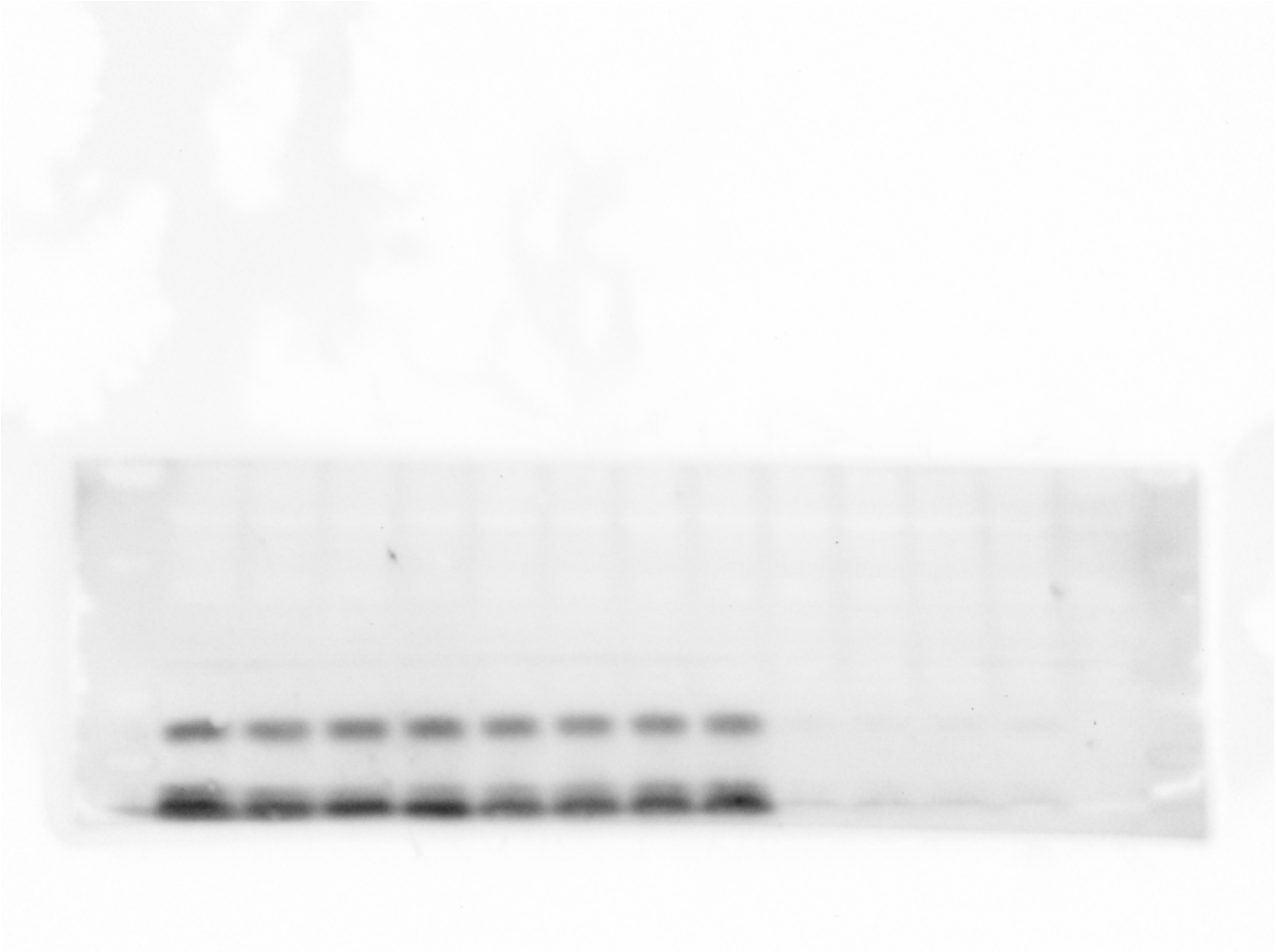

Females - Ng2 - mb.tif

2244 x 1673

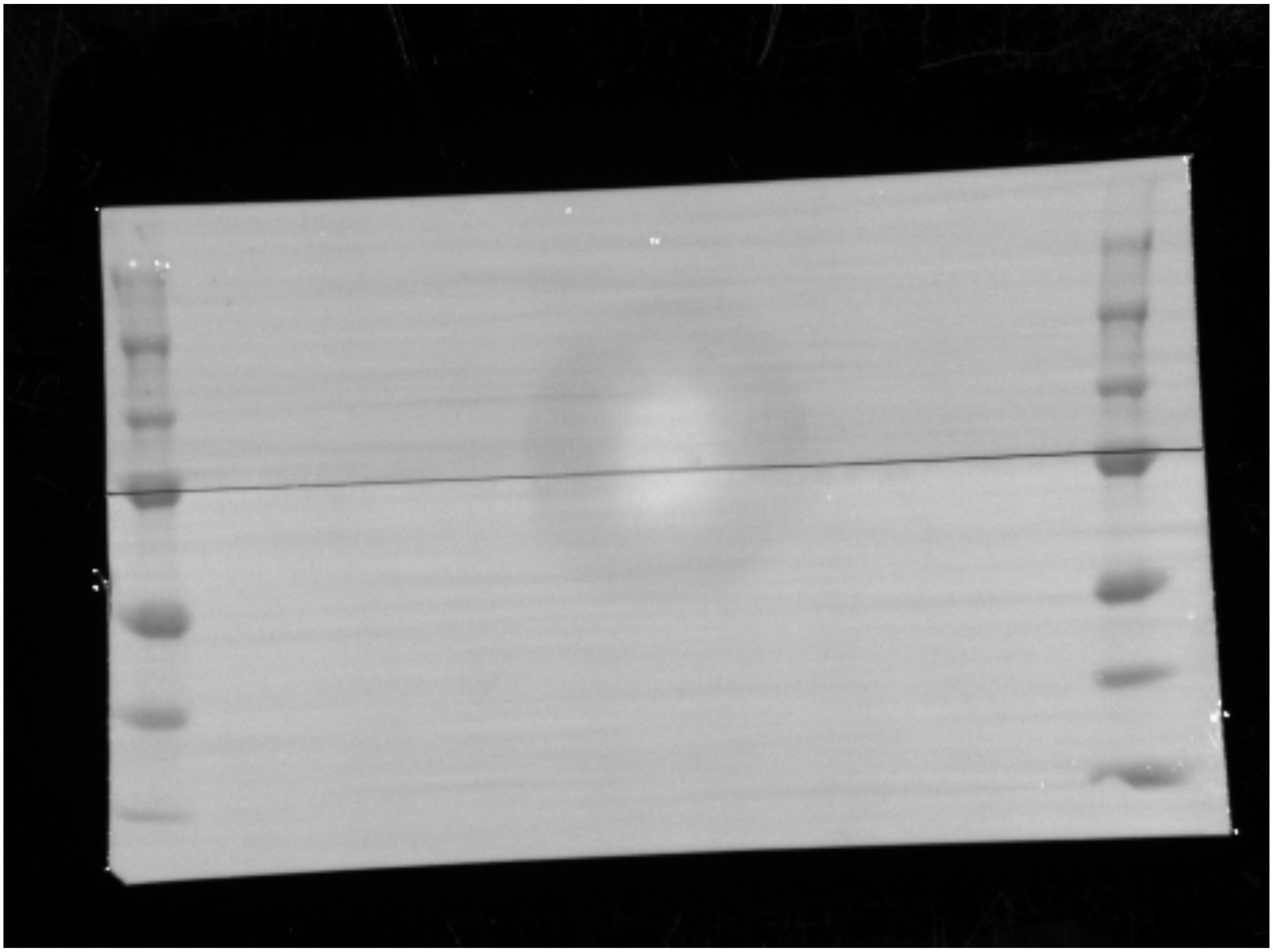

Females - Ng2 - pt.tif

2480 x 1853

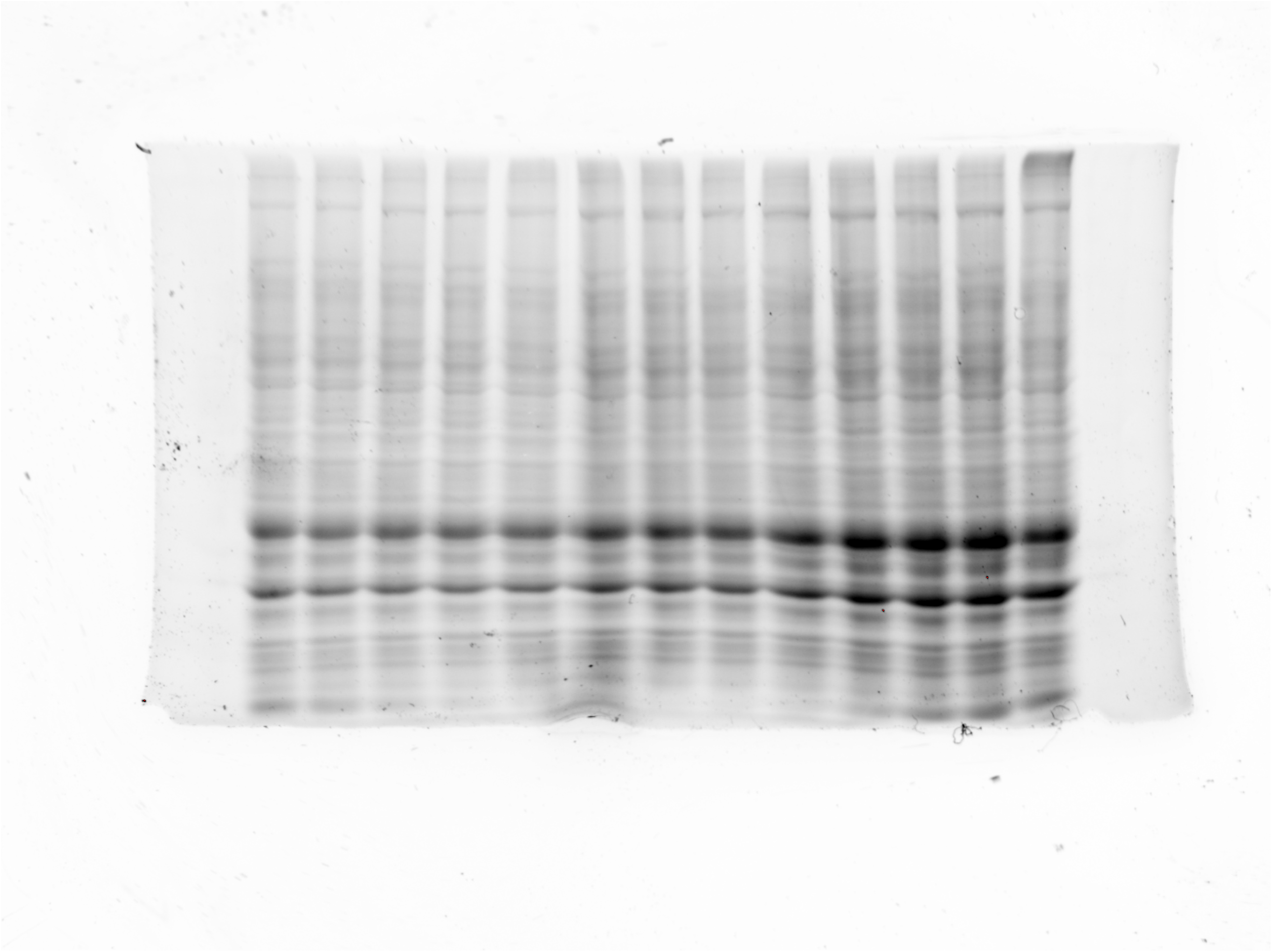

Females - Ng2 - tp.tif
2244 x 1676

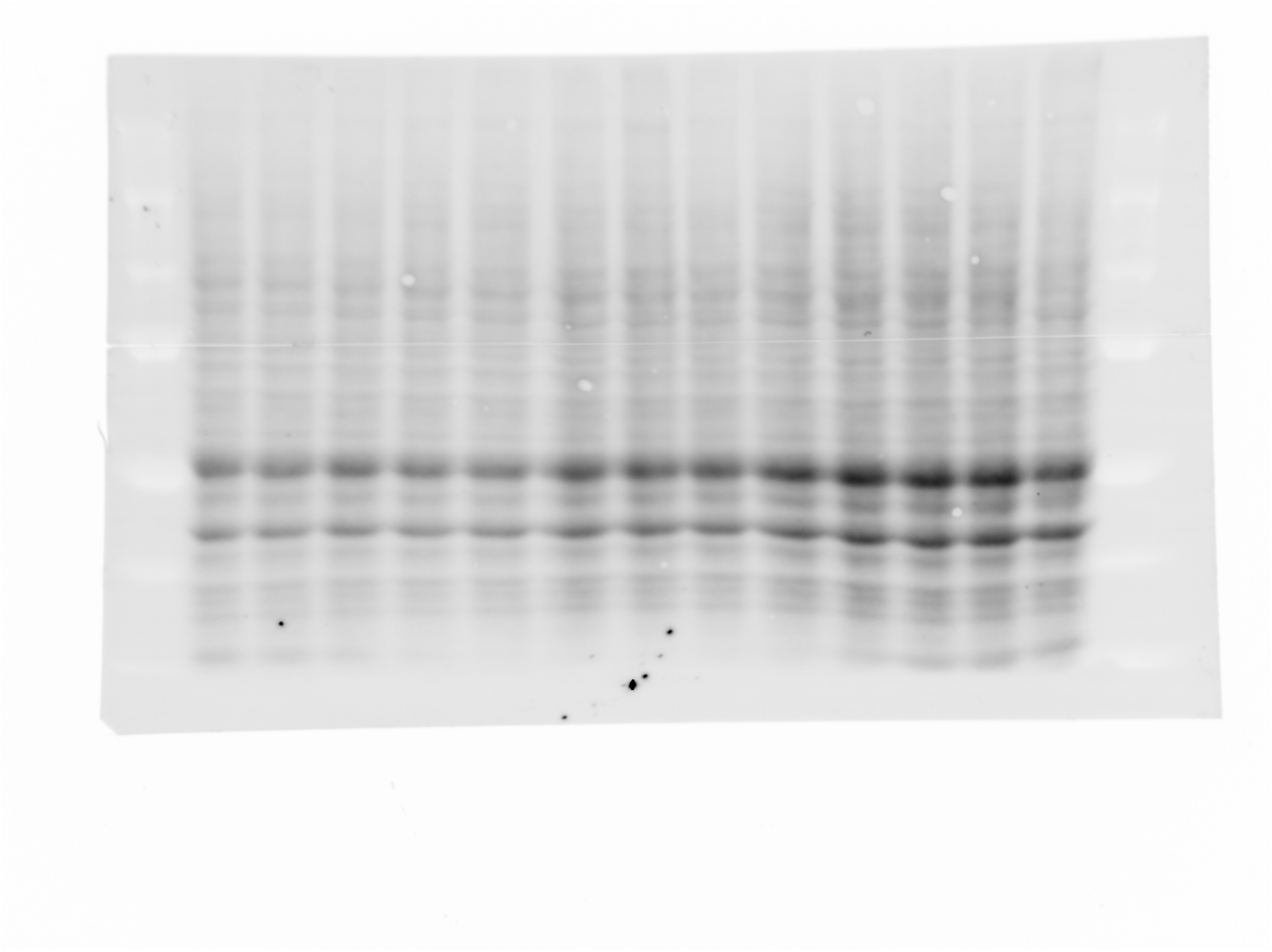

Females - Ng2.tif

2244 x 1676

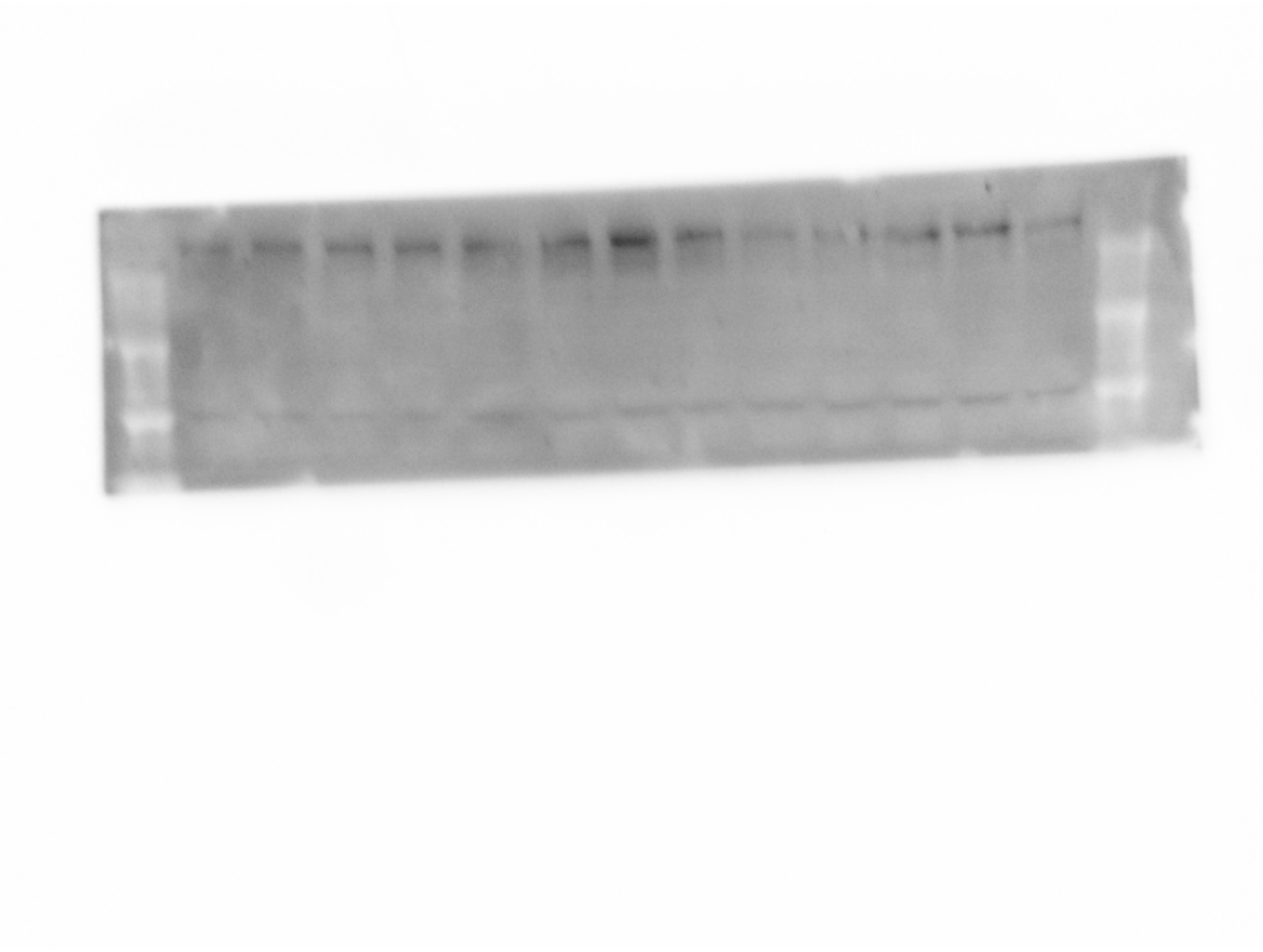

Females - Olig2 - mb.tif

2244 x 1673

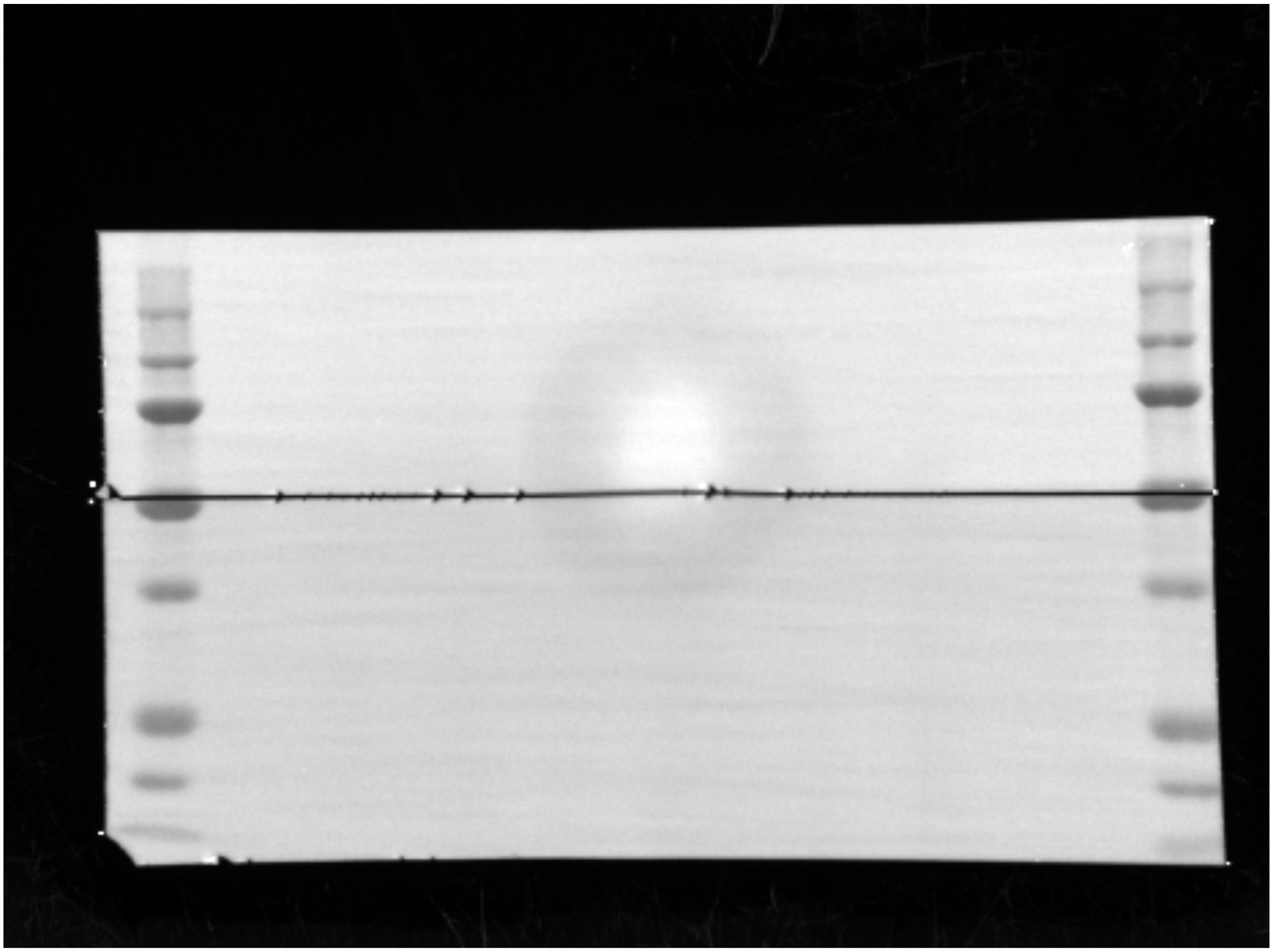

Females - Olig2 - pt.tif

2480 x 1853

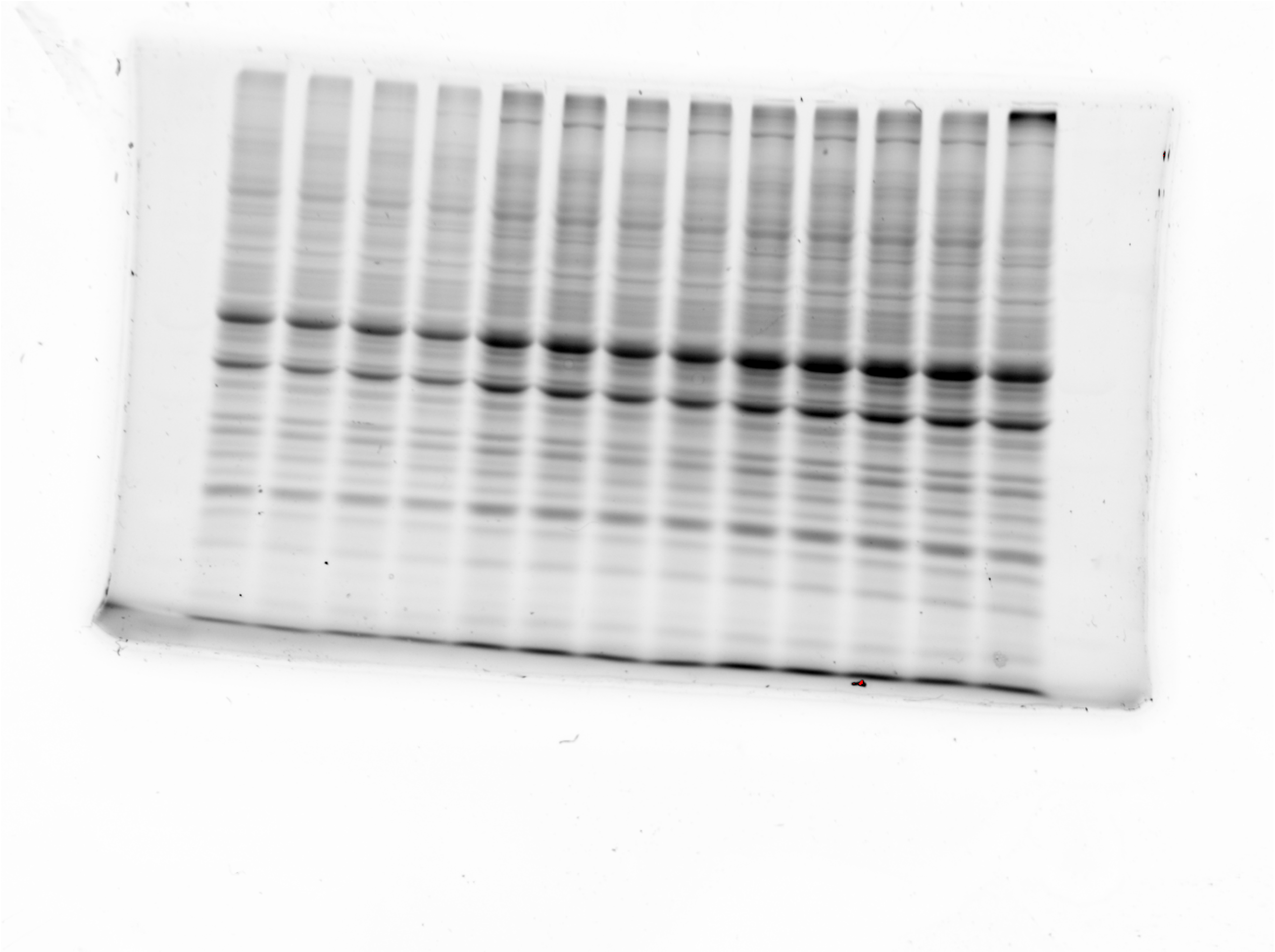

Females - Olig2 - tp.tif
2244 x 1676

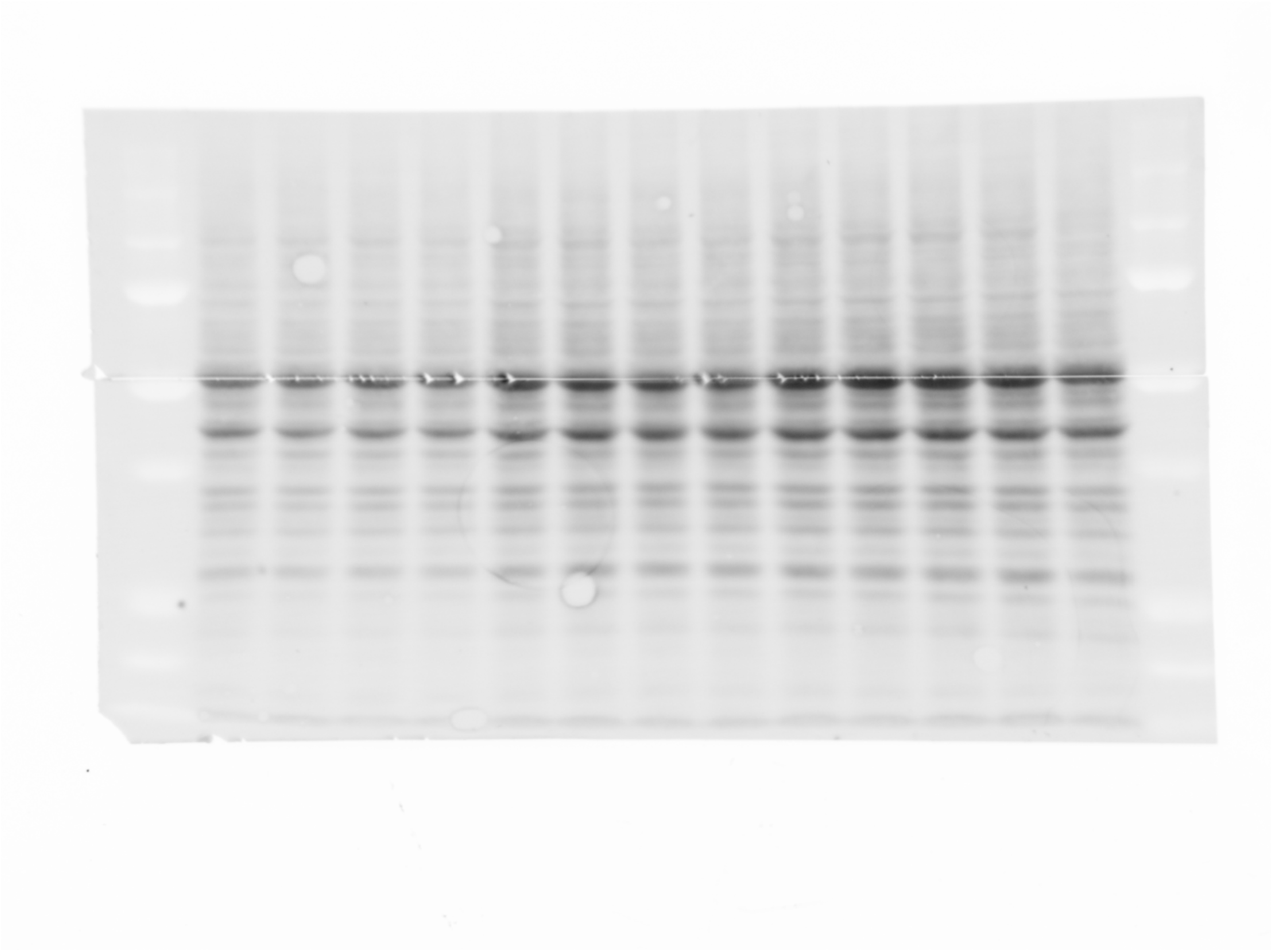

Females - Olig2.tif

2244 x 1676

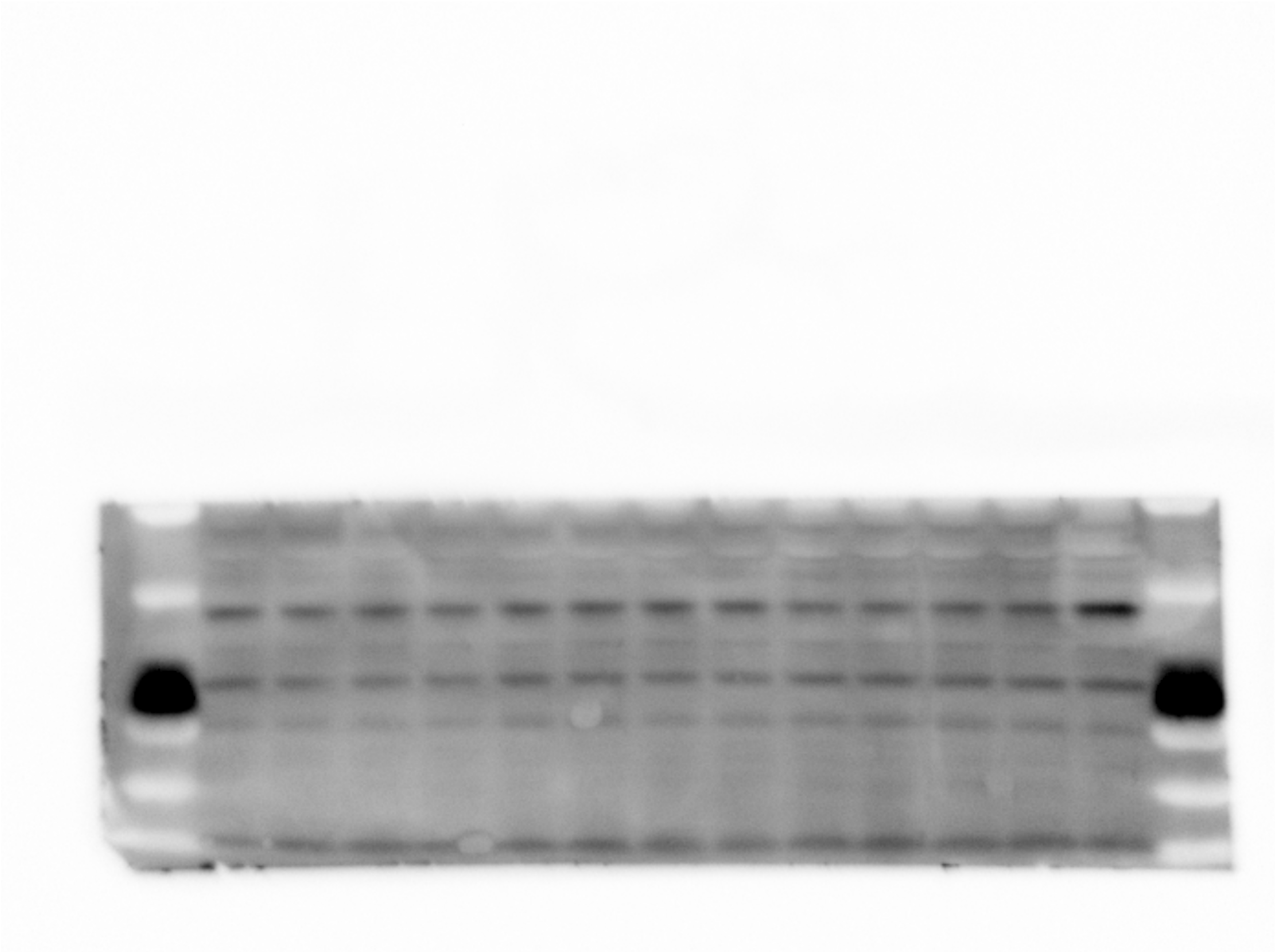

Females - Pdgfra - mb.tif

2362 x 1761

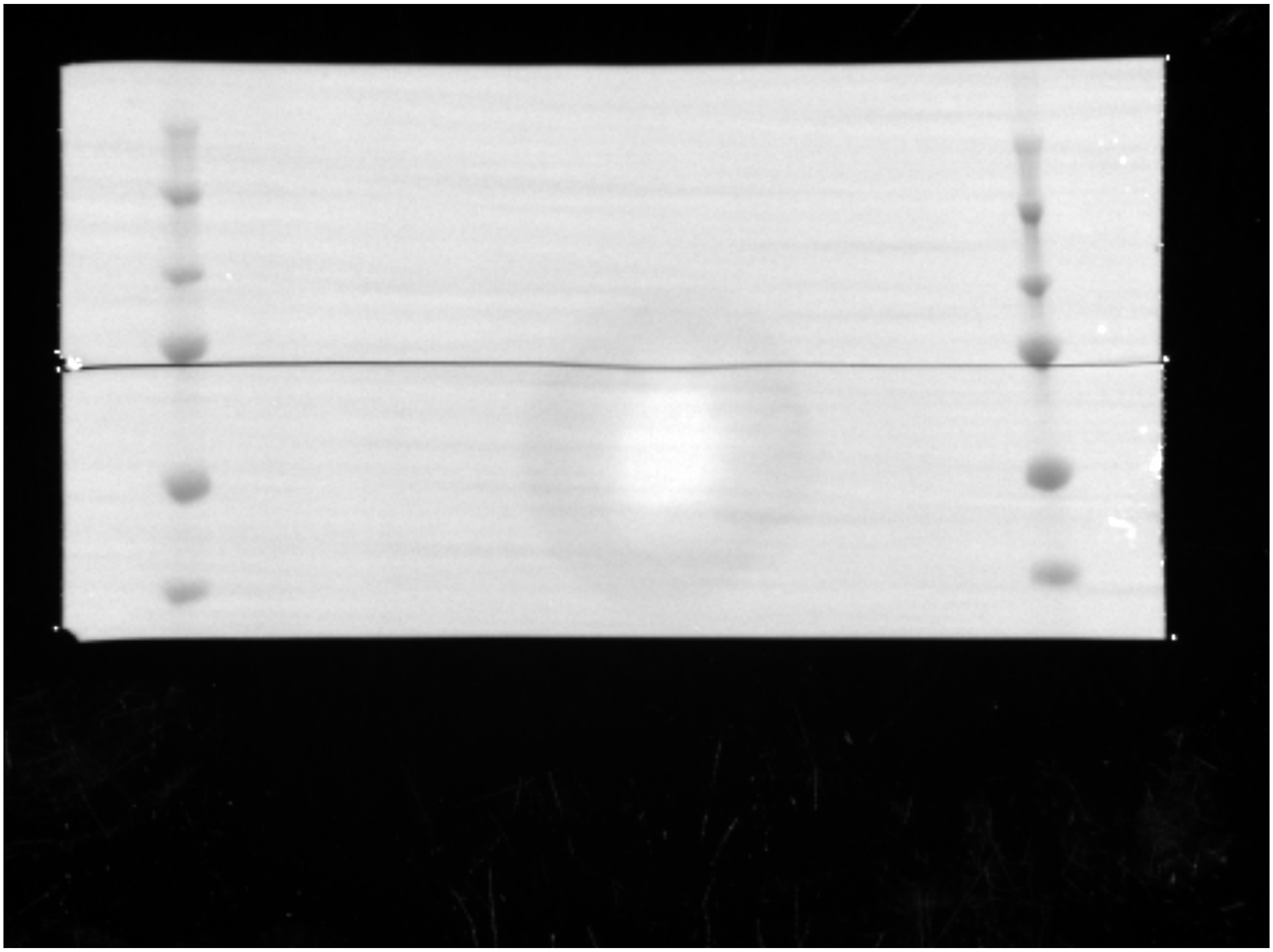

Females - Pdgfra - pt.tif

2598 x 1941

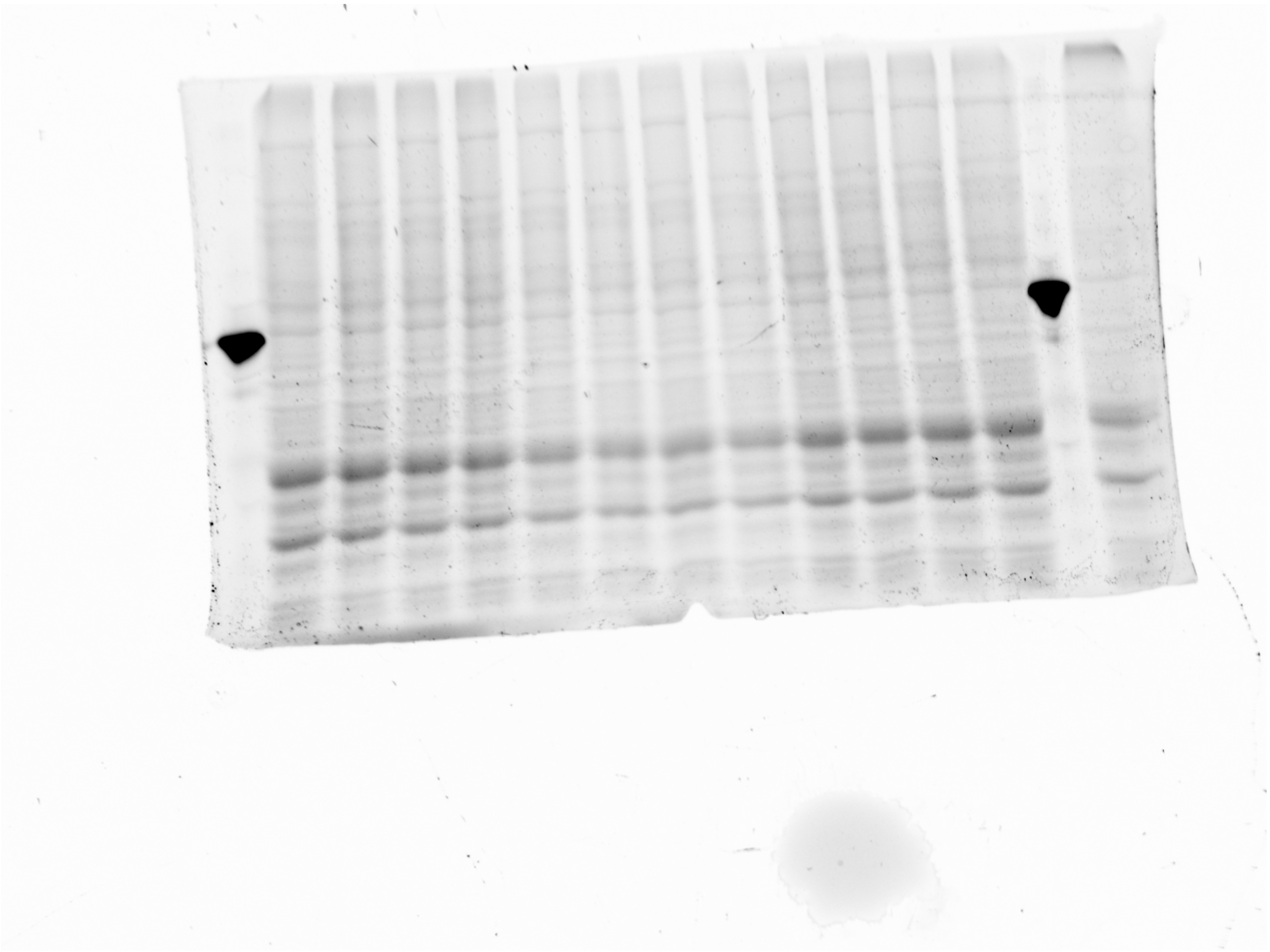

Females - Pdgfra - tp.tif

2362 x 1764

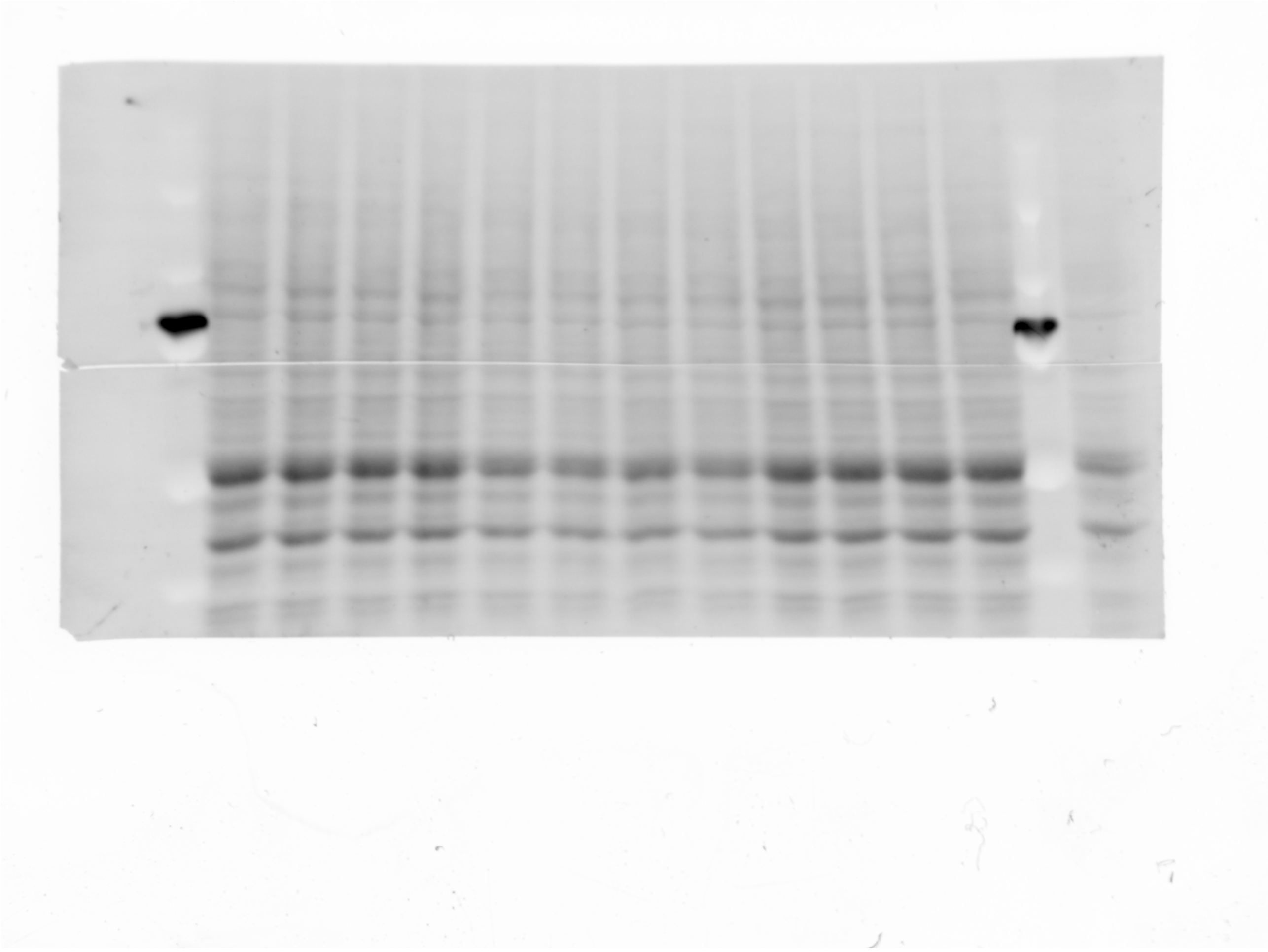

Females - Pdgfra.tif

2362 x 1764

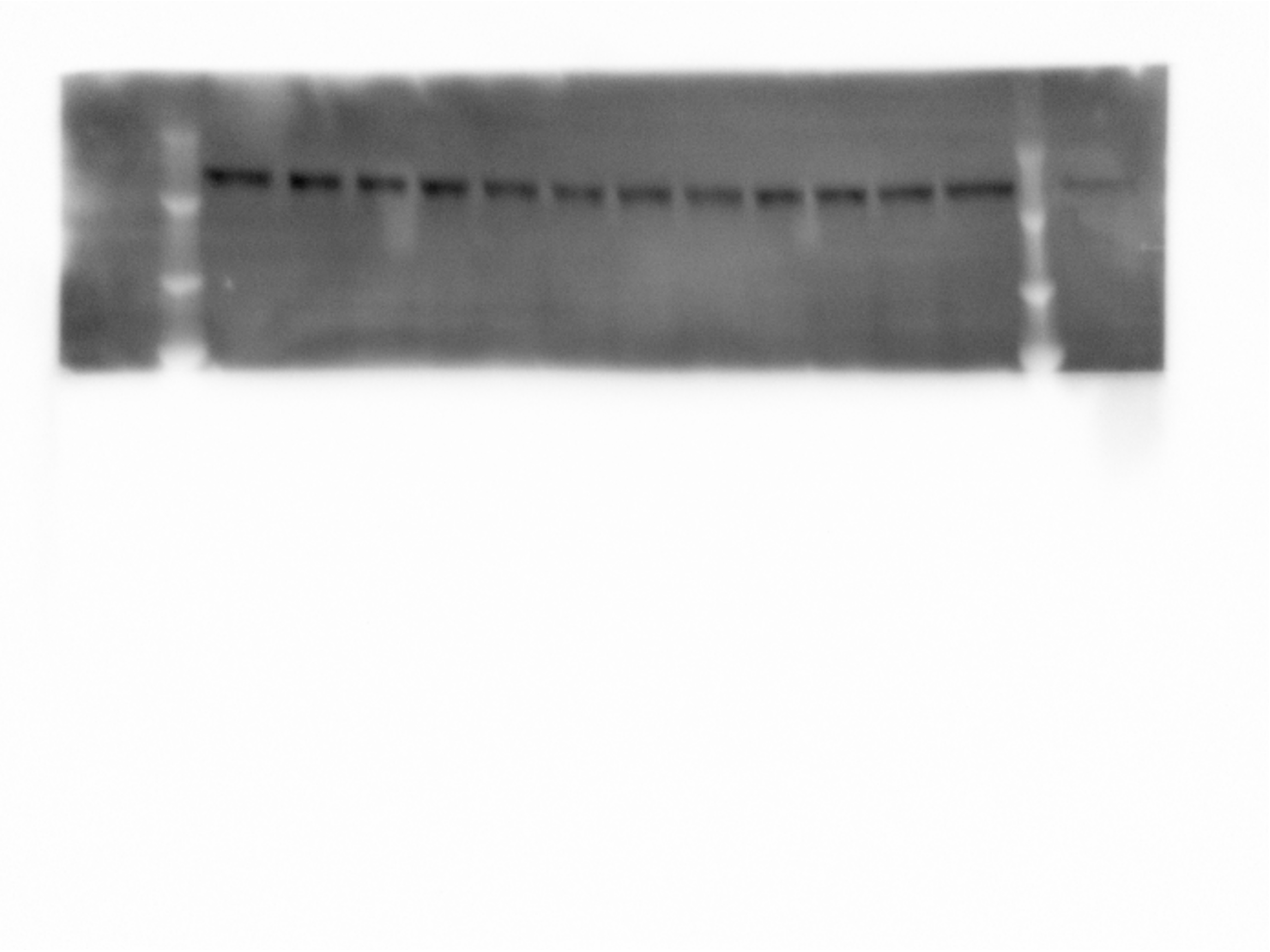

Females - Polr3a - mb.tif

2244 x 1673

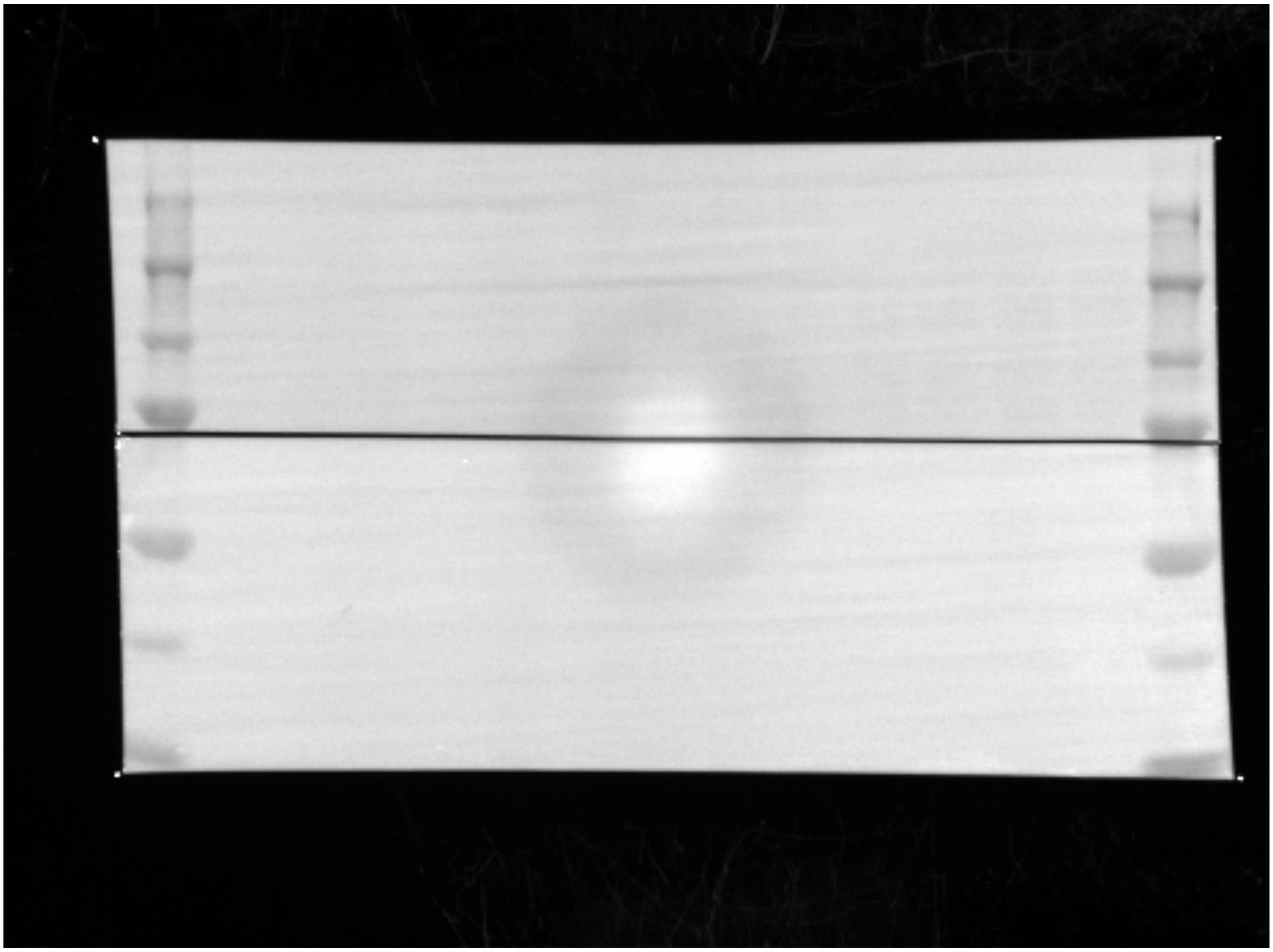

Females - Polr3a - pt.tif

2480 x 1853

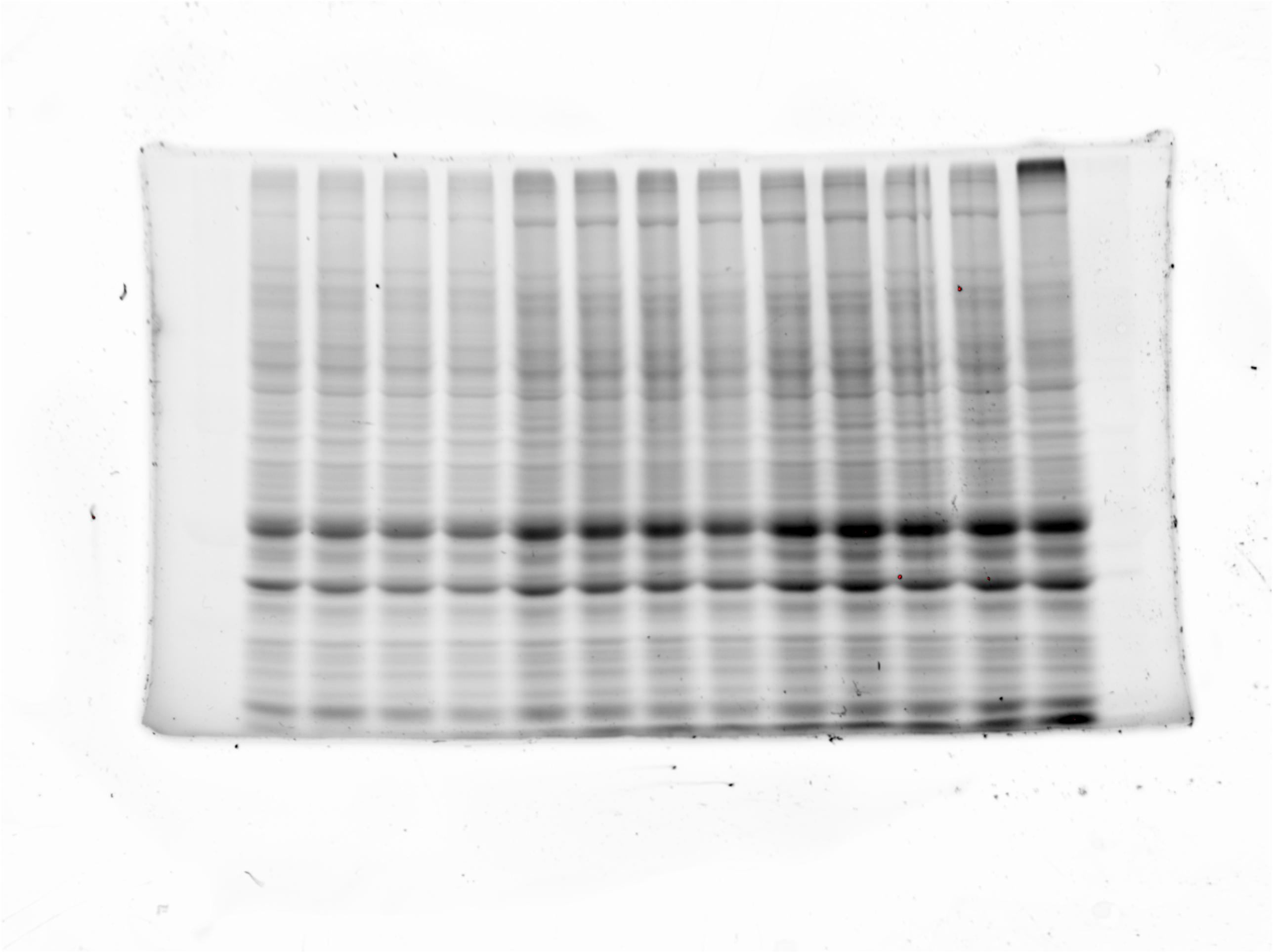

Females - Polr3a - tp.tif

2244 x 1676

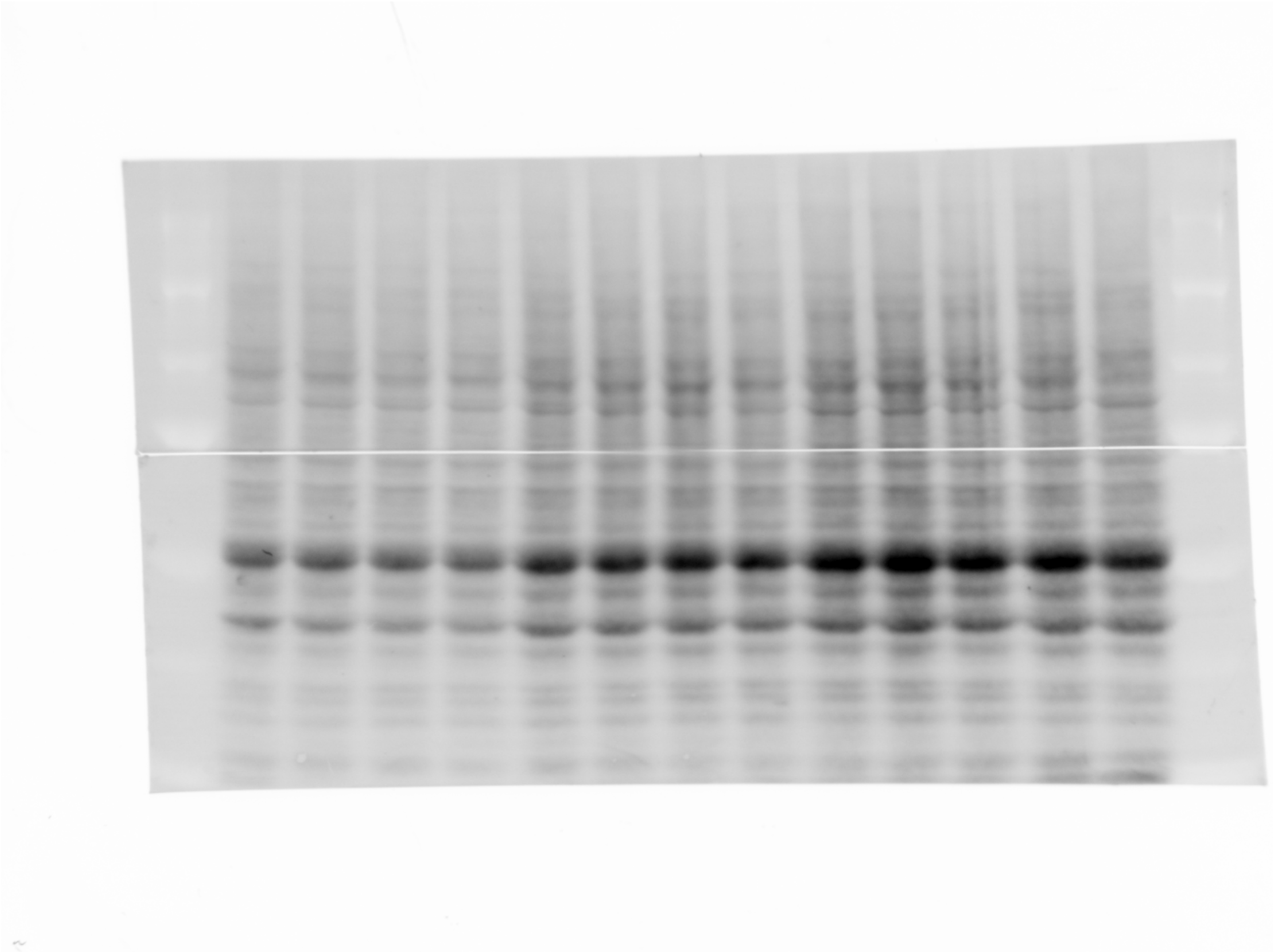

Females - Polr3a.tif

2244 x 1676

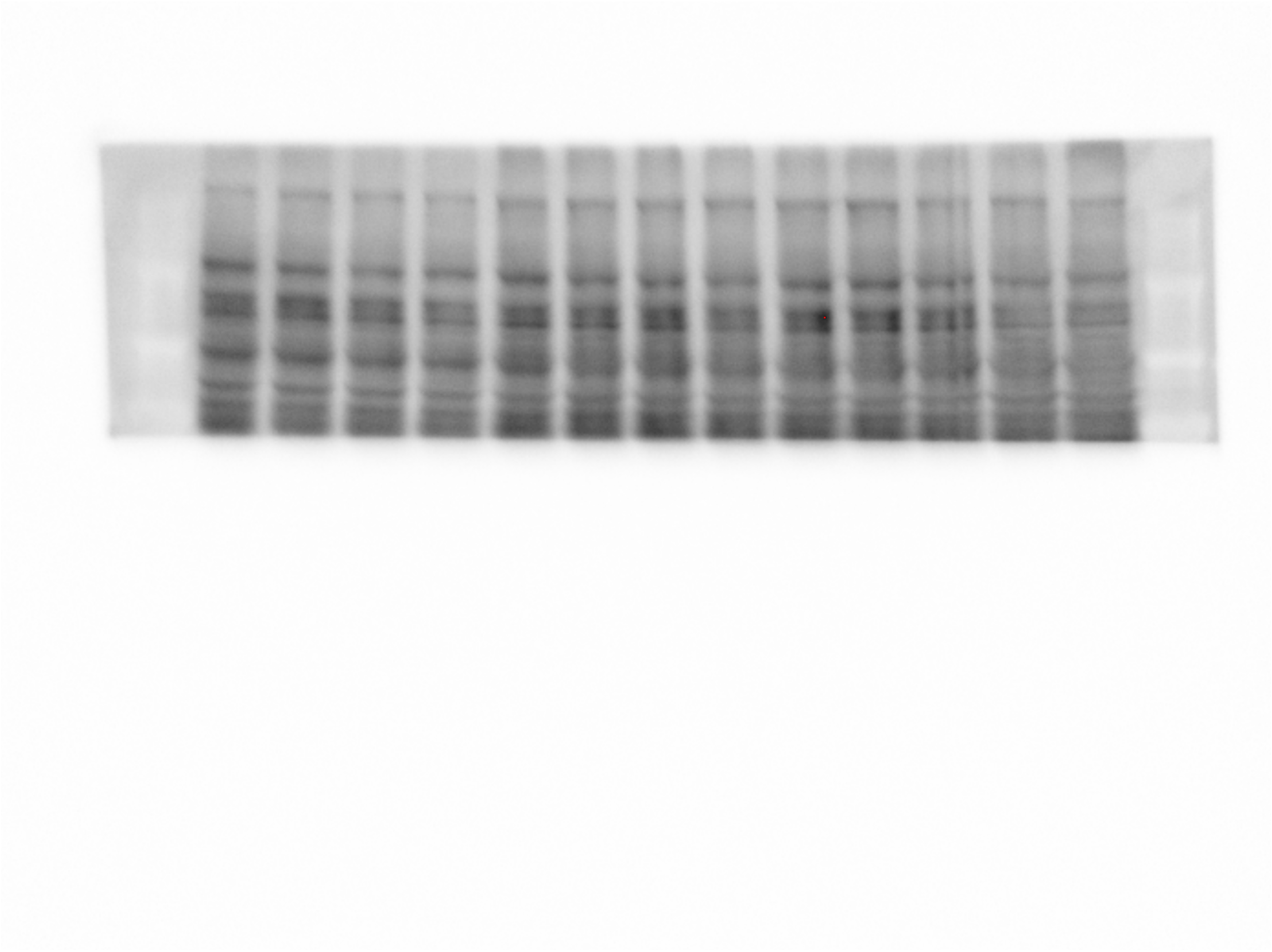

Females - Polr3b - mb.tif

2244 x 1673

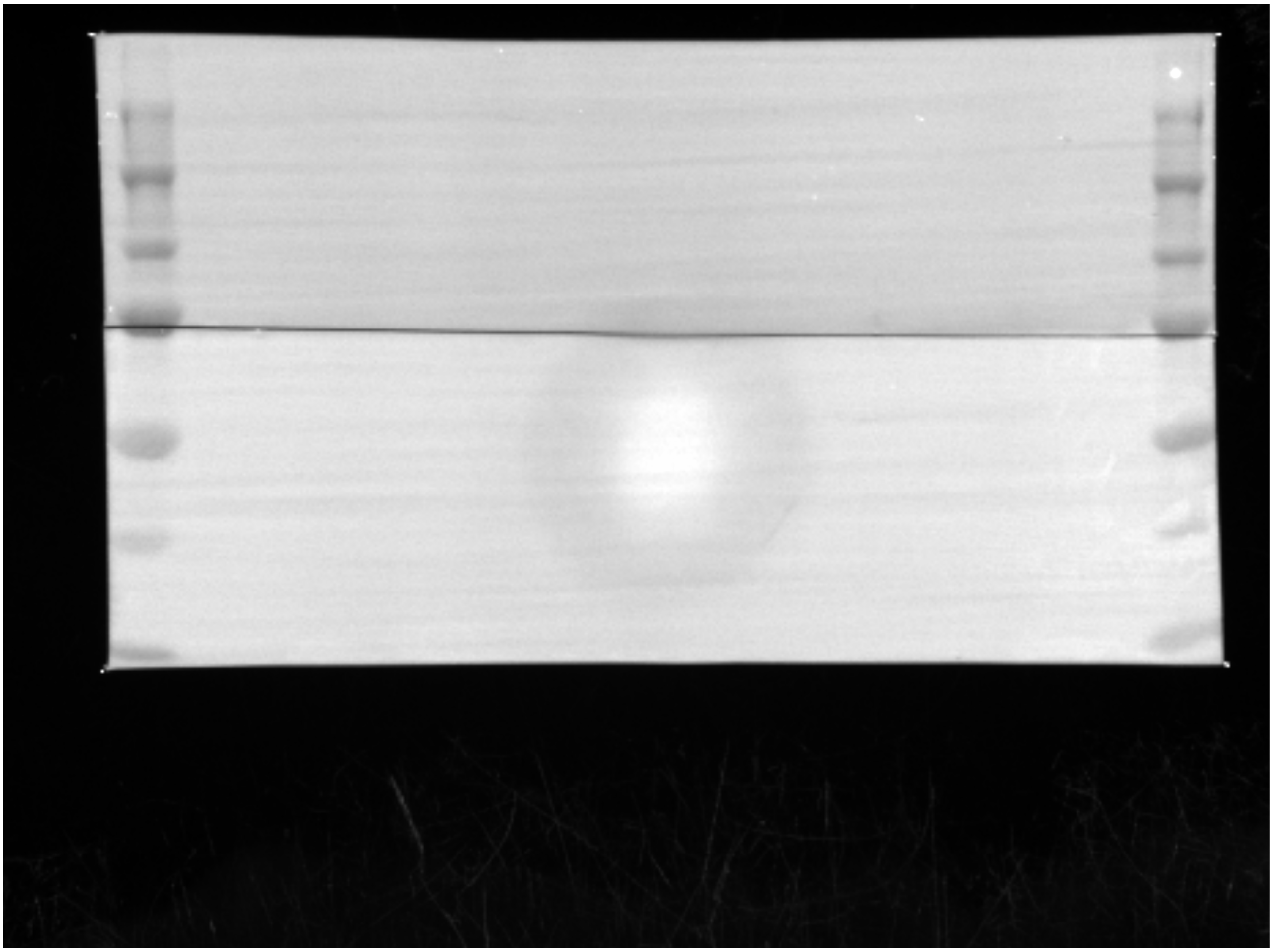

Females - Polr3b - pt.tif

2480 x 1853

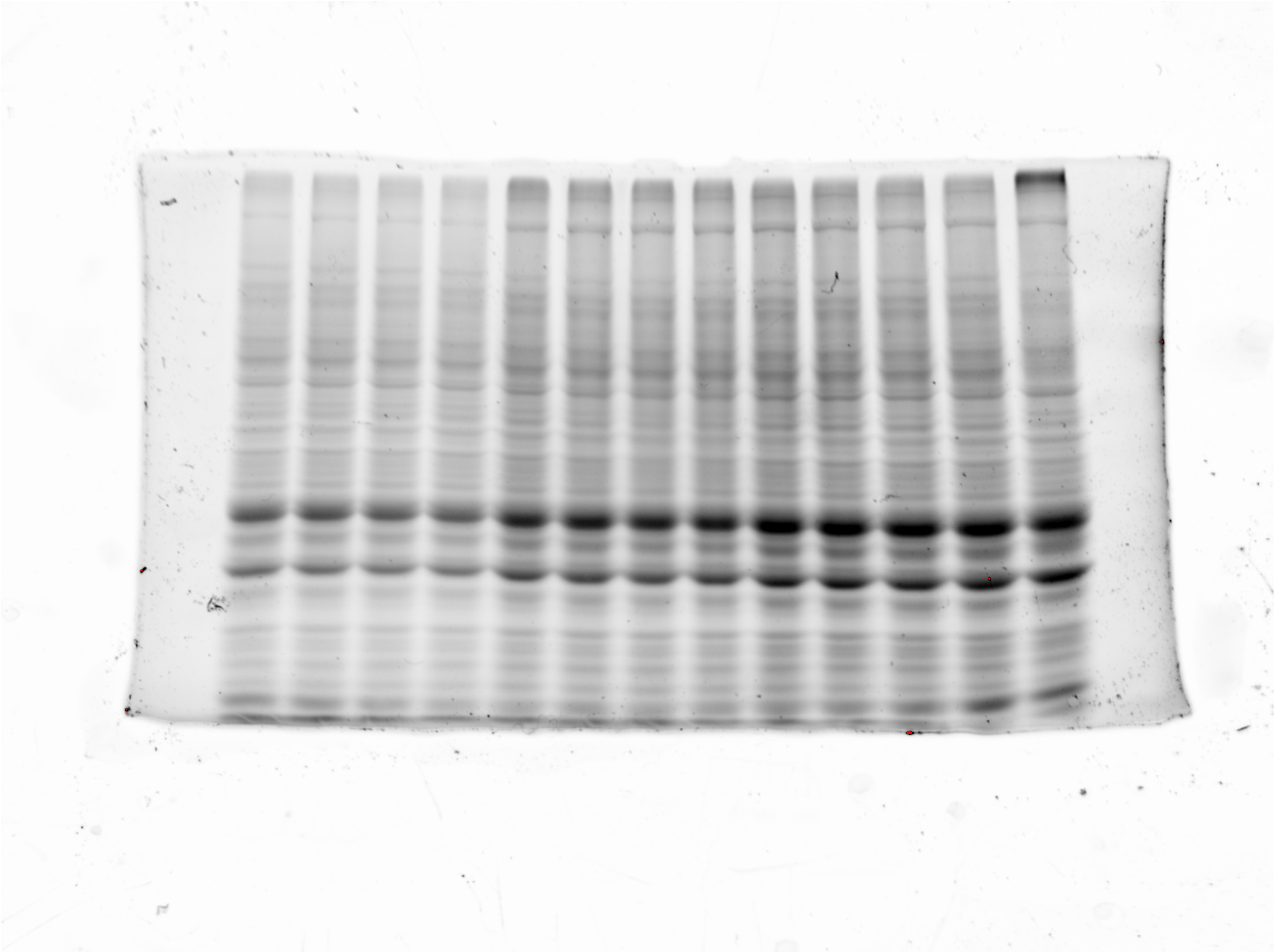

Females - Polr3b - tp.tif
2244 x 1676

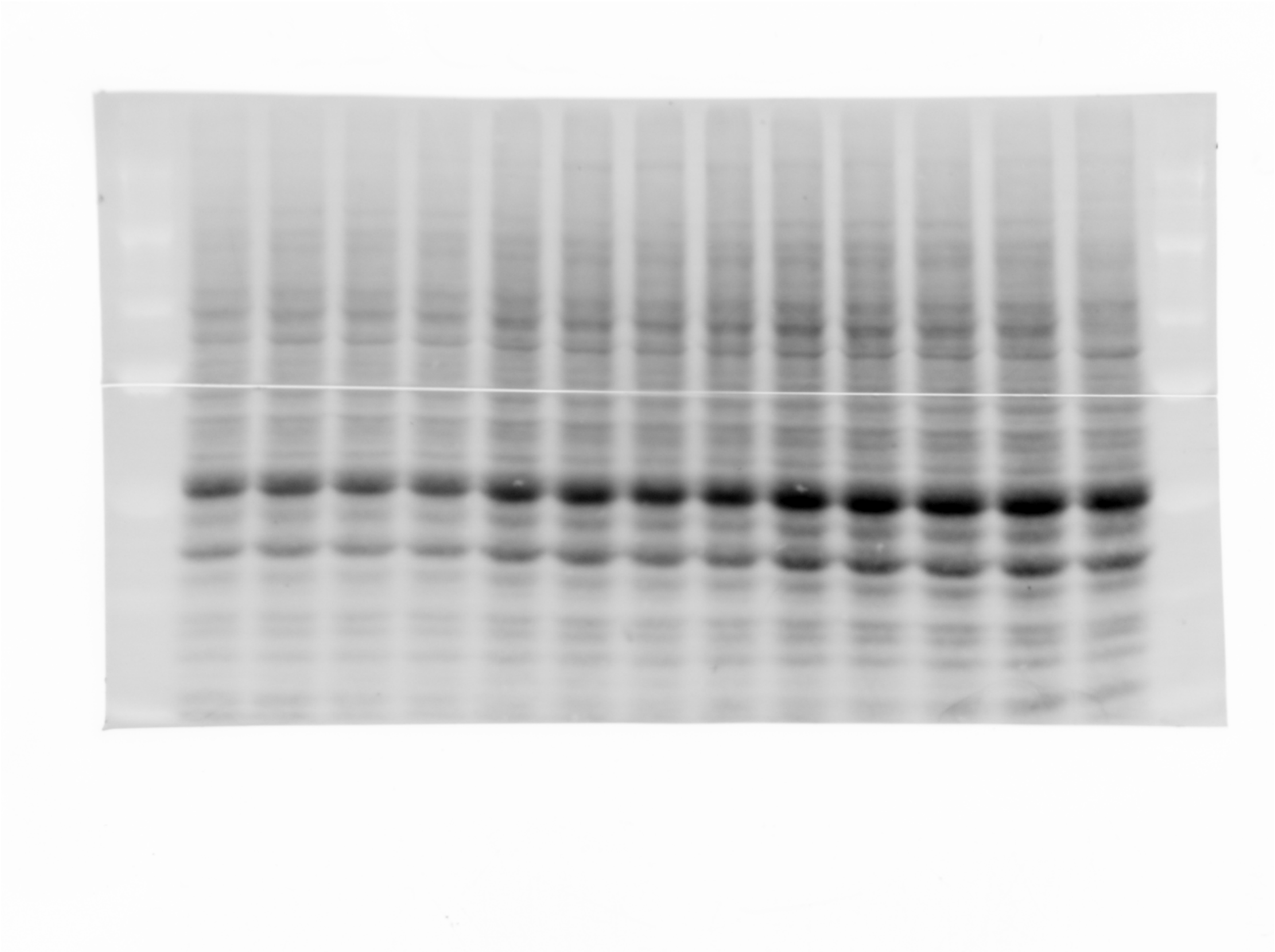

Females - Polr3b.tif

2244 x 1676

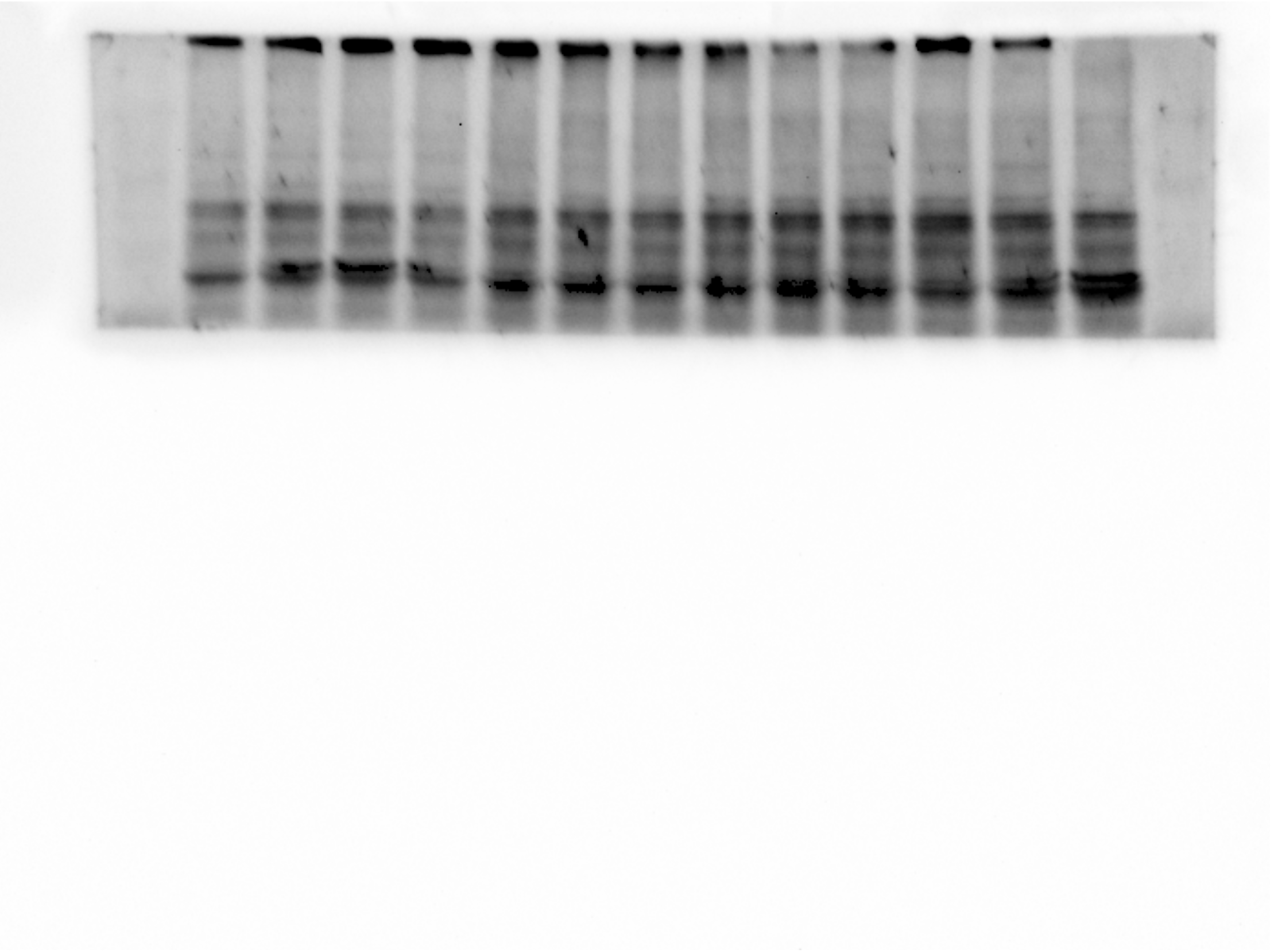

Males - Mbp - mb.tif

2551 x 1902

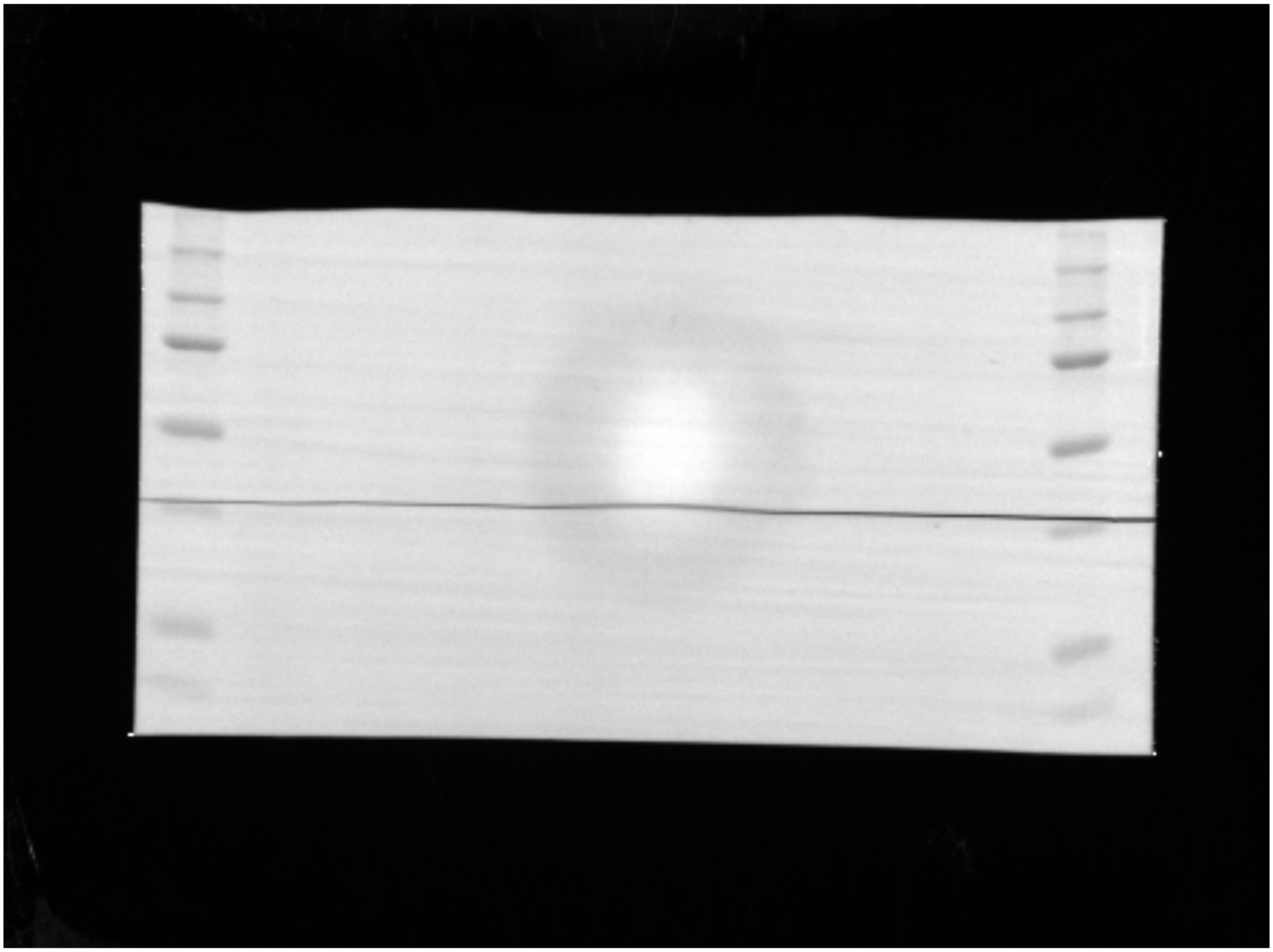

Males - Mbp - pt.tif

2064 x 1200

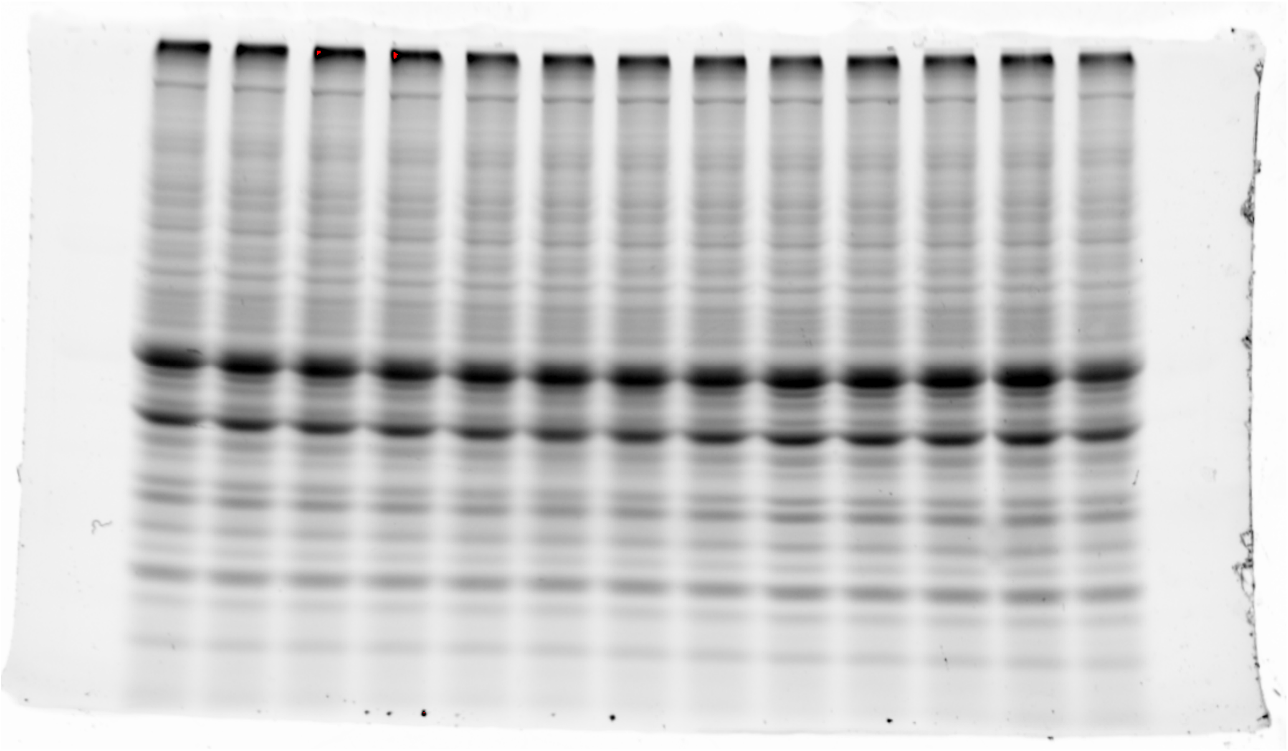

Males - Mbp - tp.tif

2551 x 1906

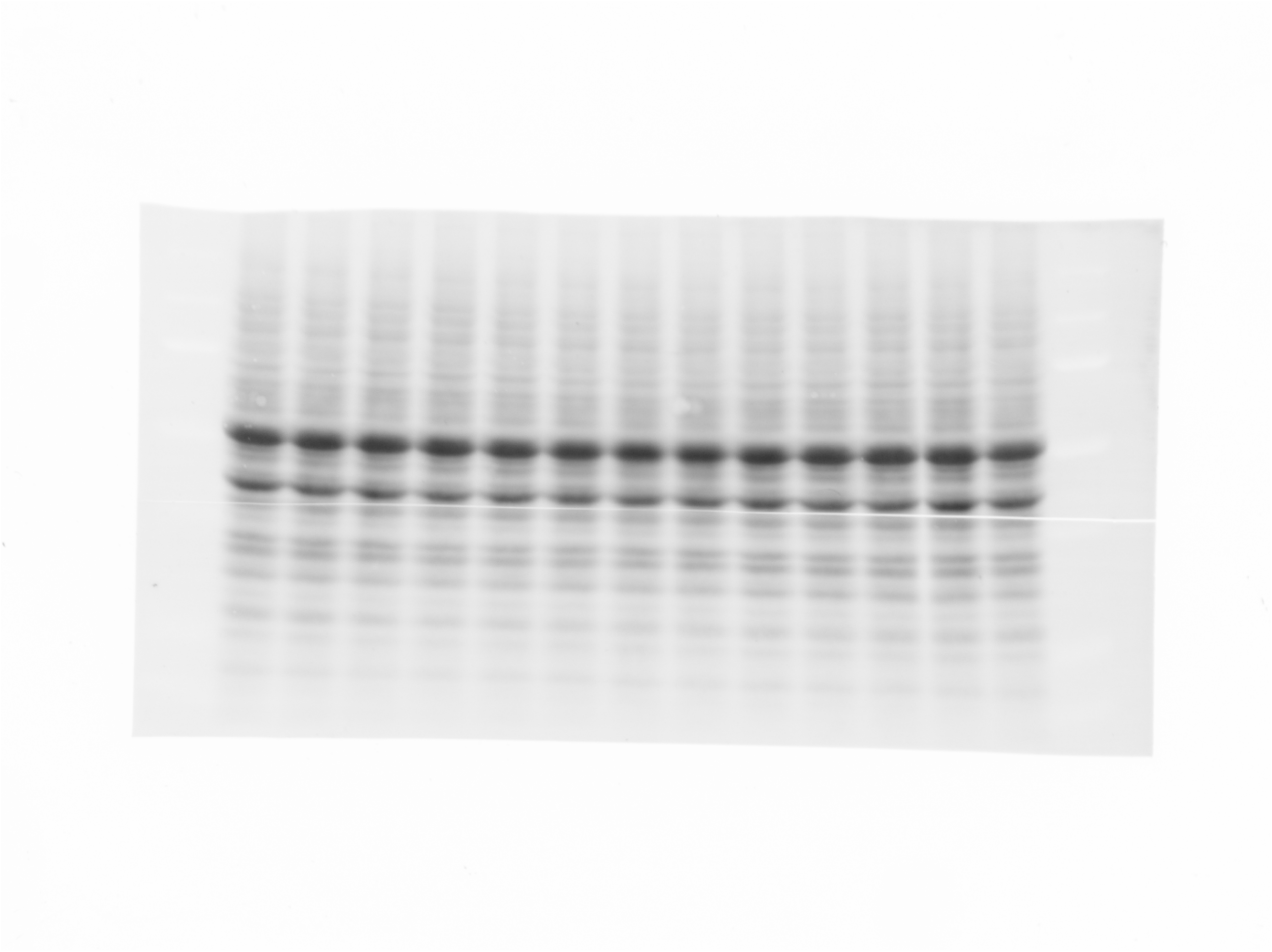

Males - Mbp.tif

2551 x 1906

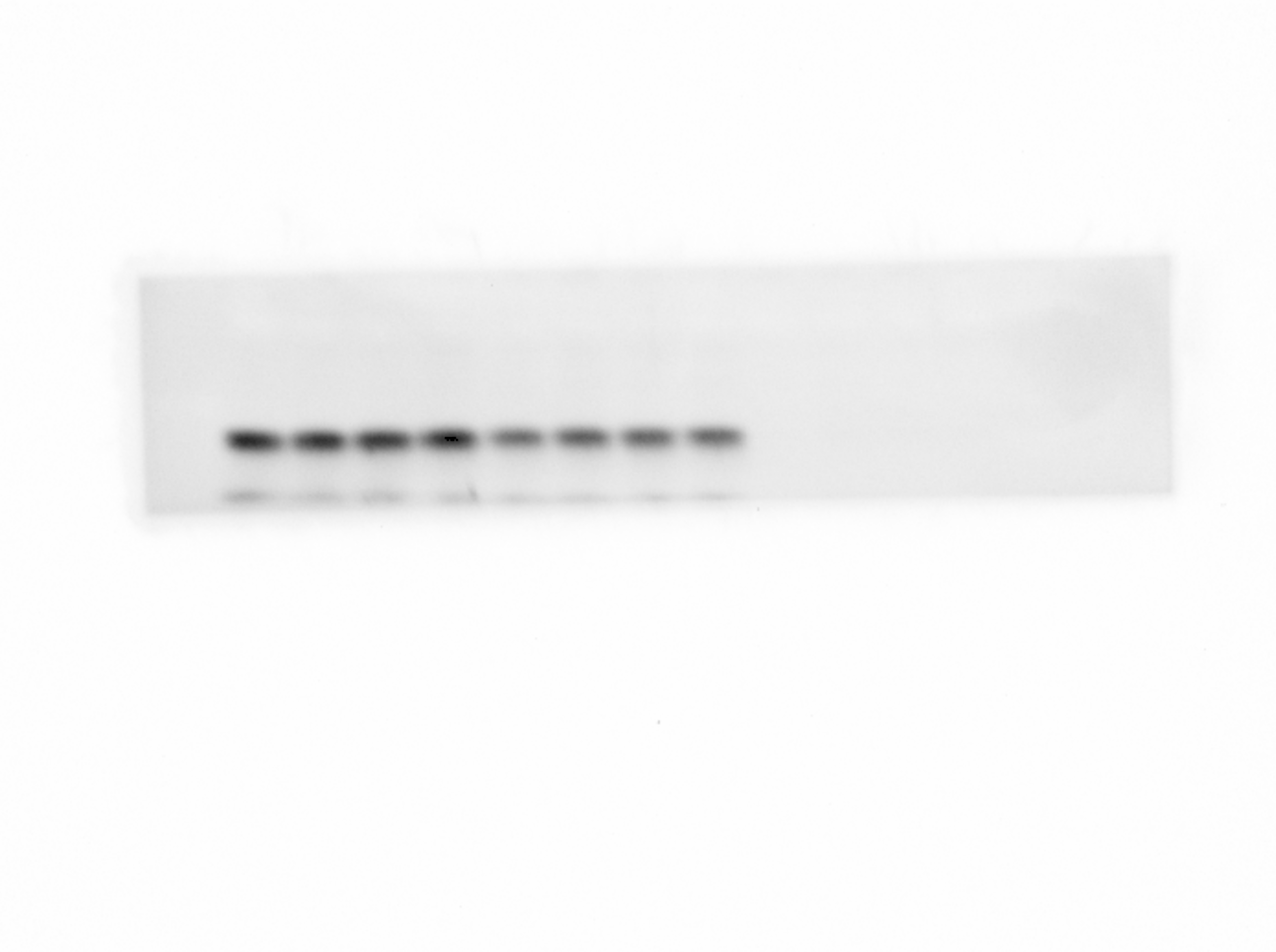

Males - Ng2 - mb.tif
2551 x 1902

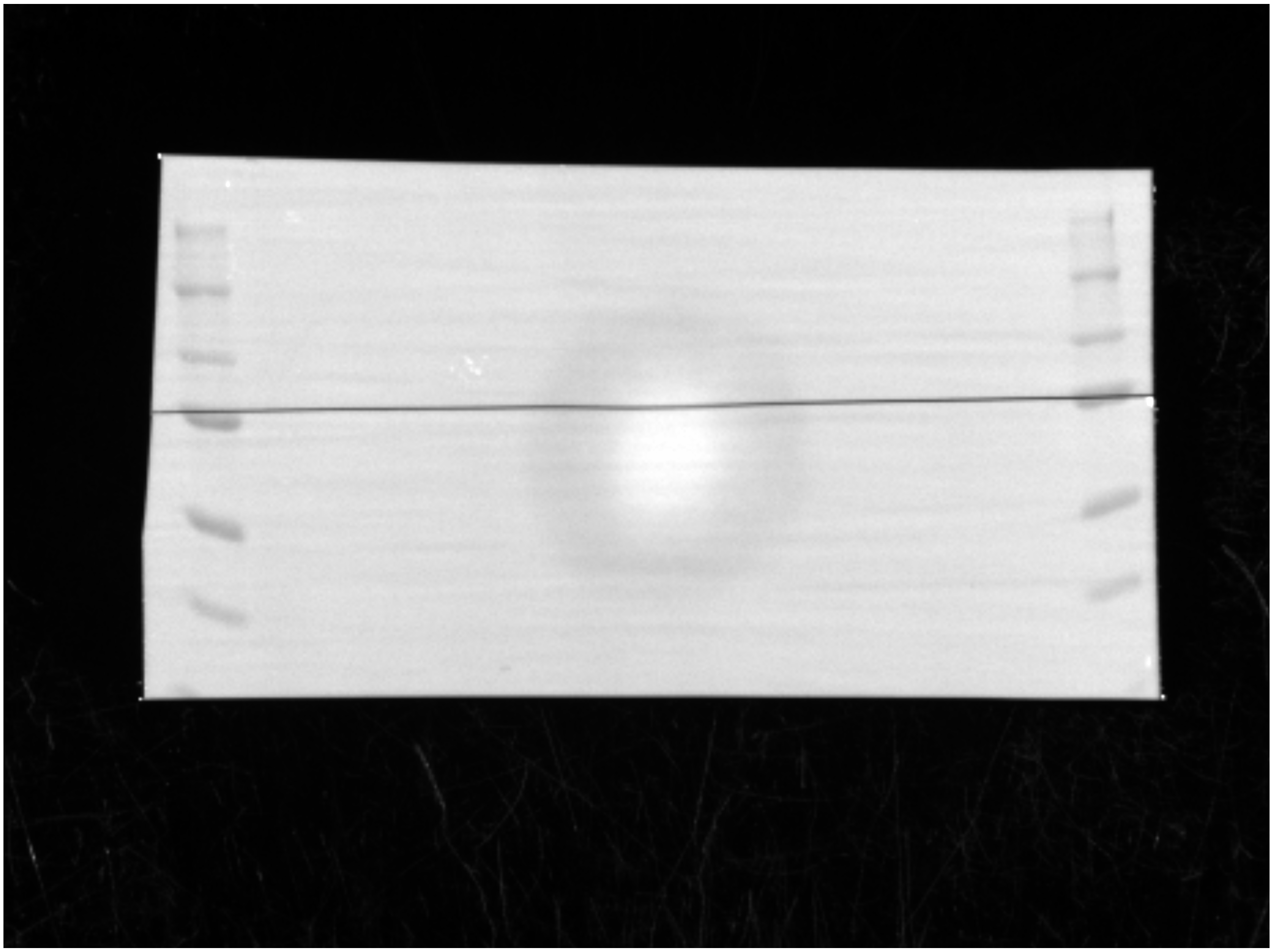

Males - Ng2 - pt.tif

2055 x 1194

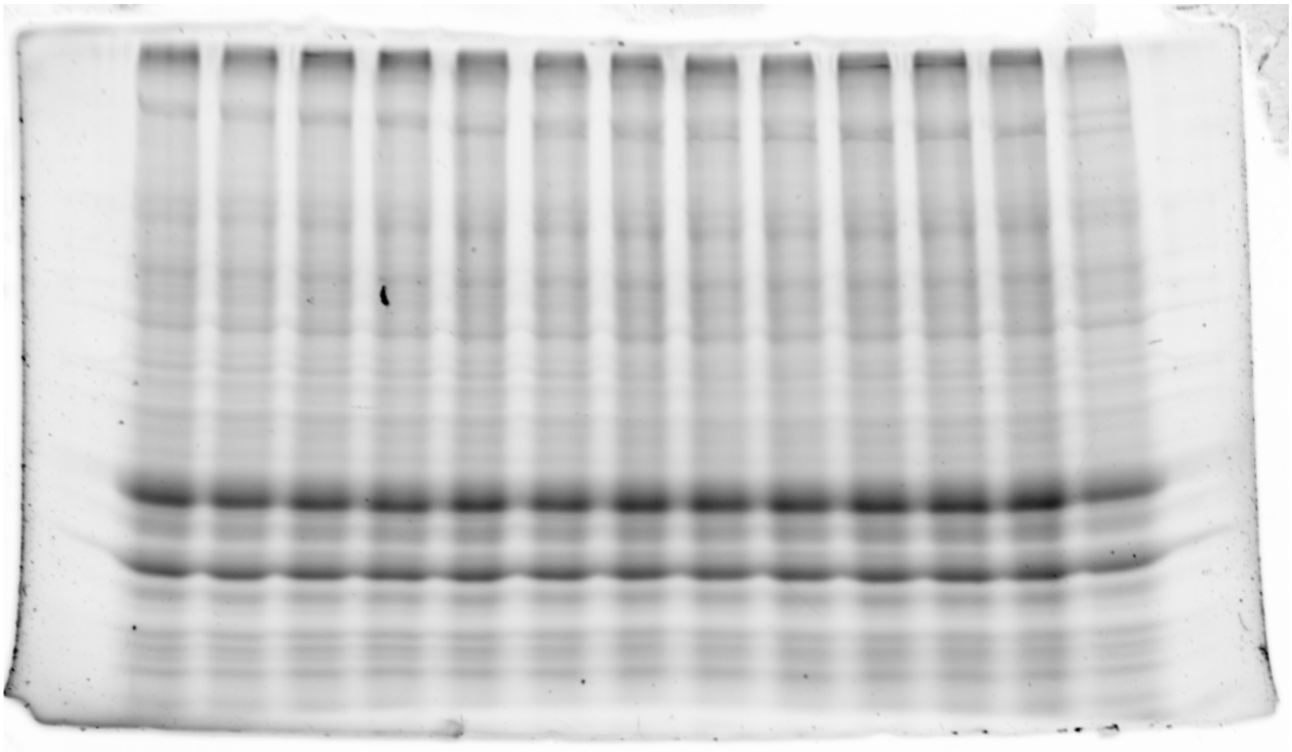

Males - Ng2 - tp.tif

2551 x 1906

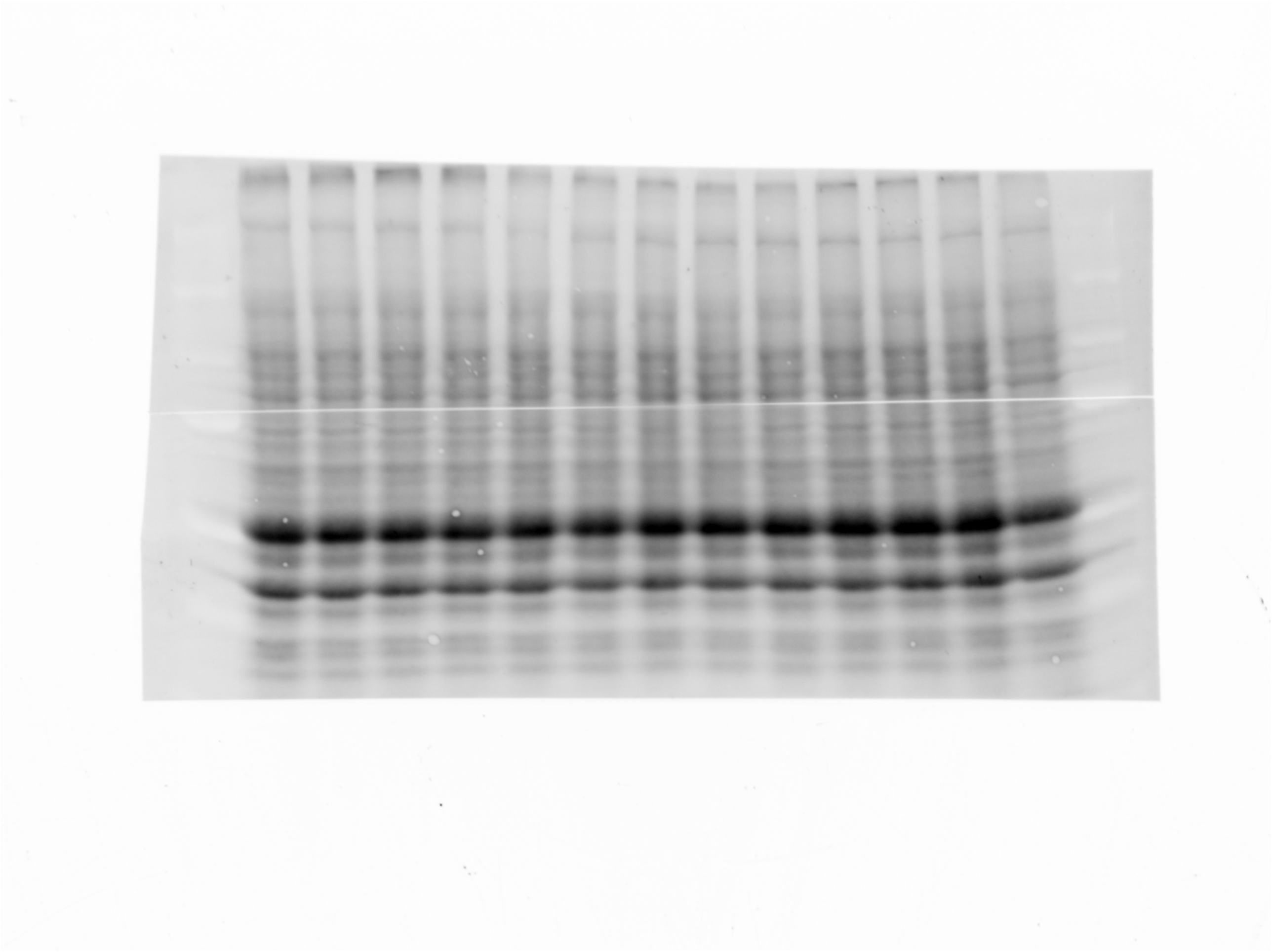

Males - Ng2.tif
2551 x 1906

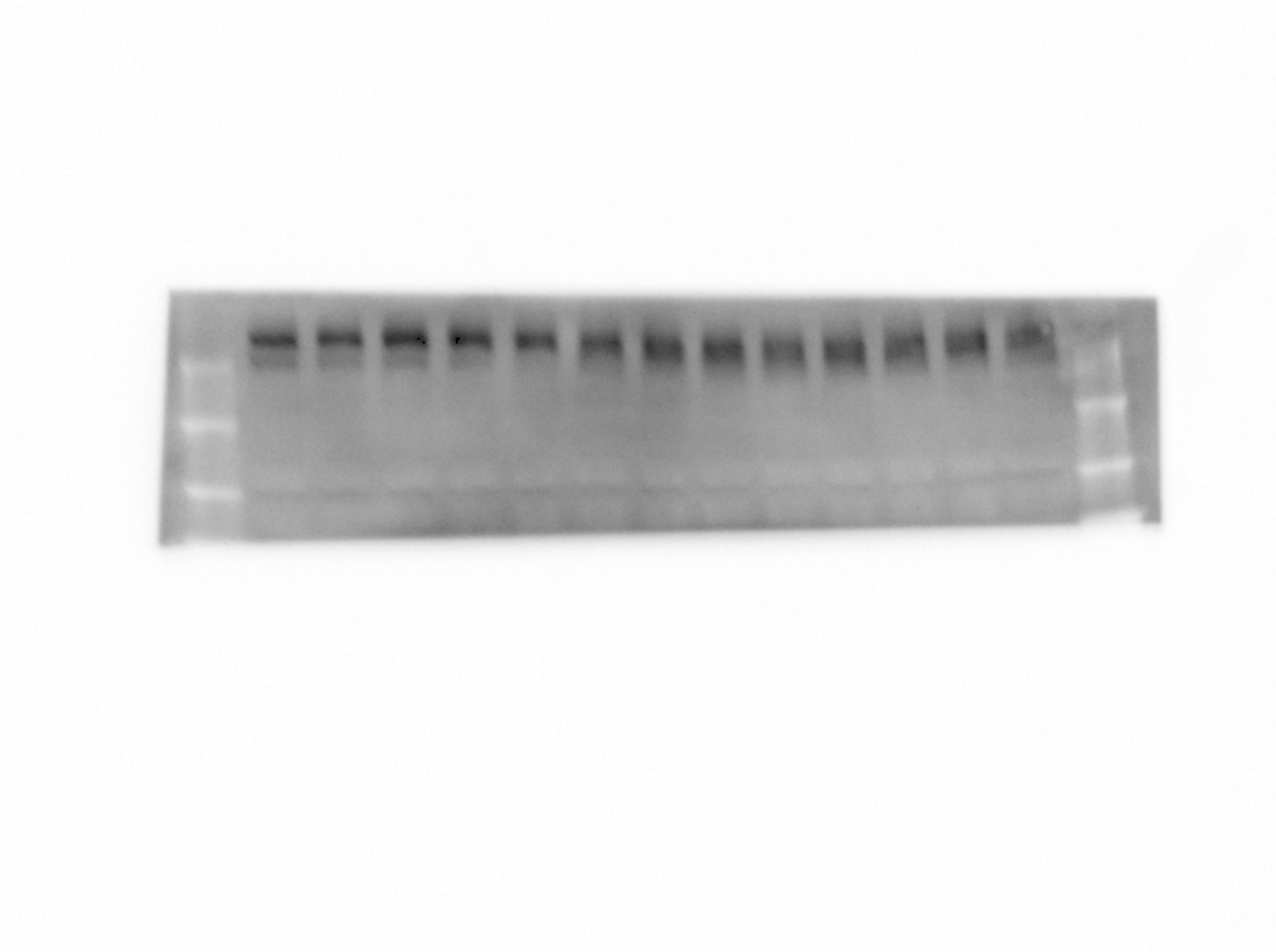

Males - Olig2 - mb.tif

2551 x 1902

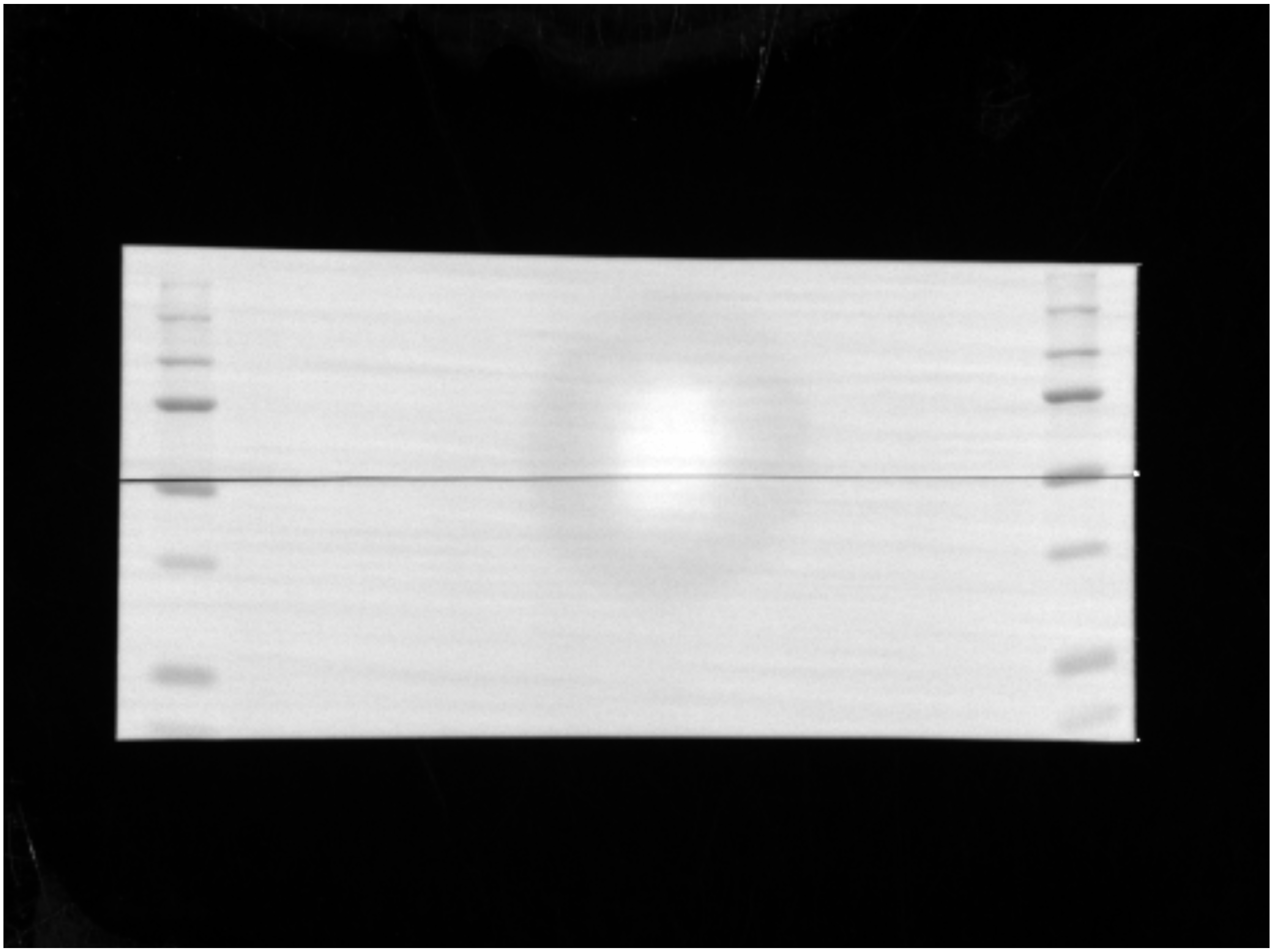

Males - Olig2 - pt.tif

2075 x 1183

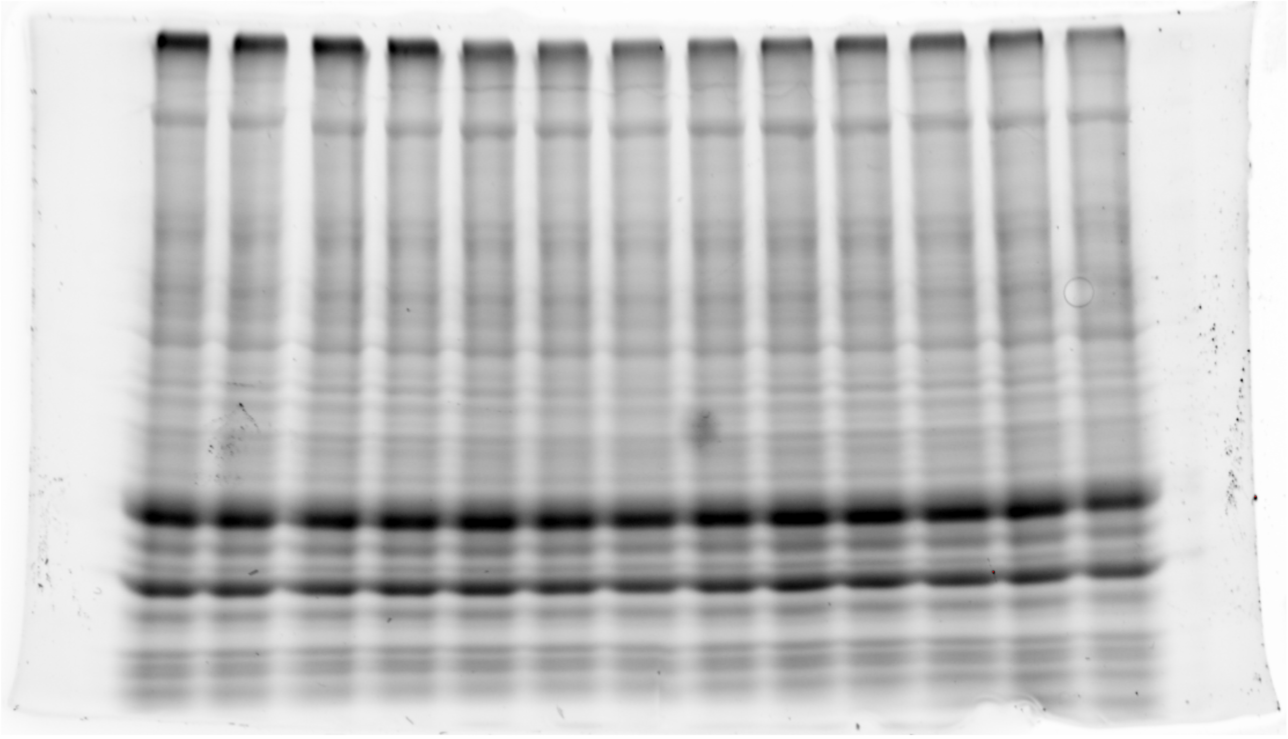

Males - Olig2 - tp.tif

2551 x 1906

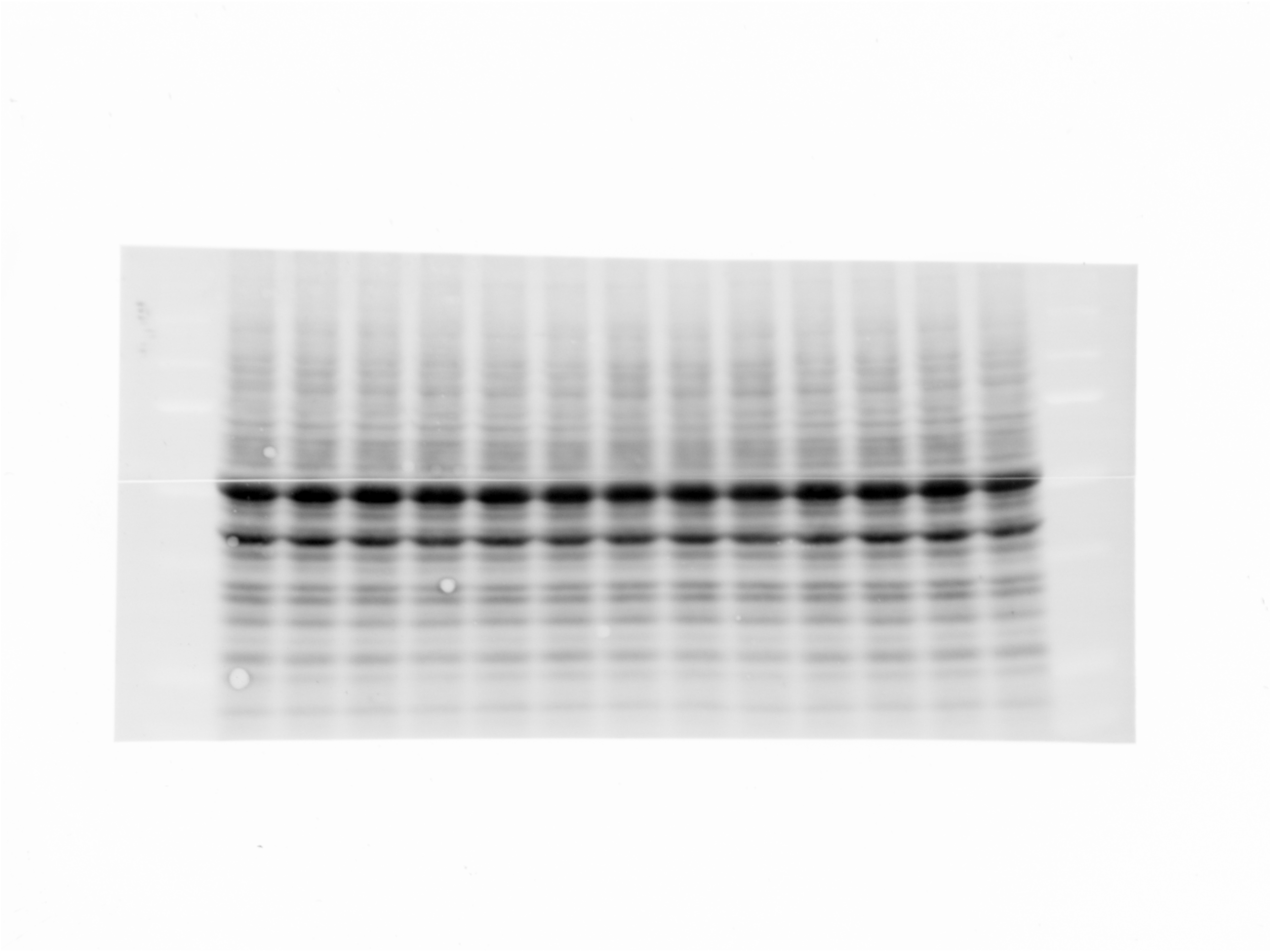

Males - Olig2.tif

2551 x 1906

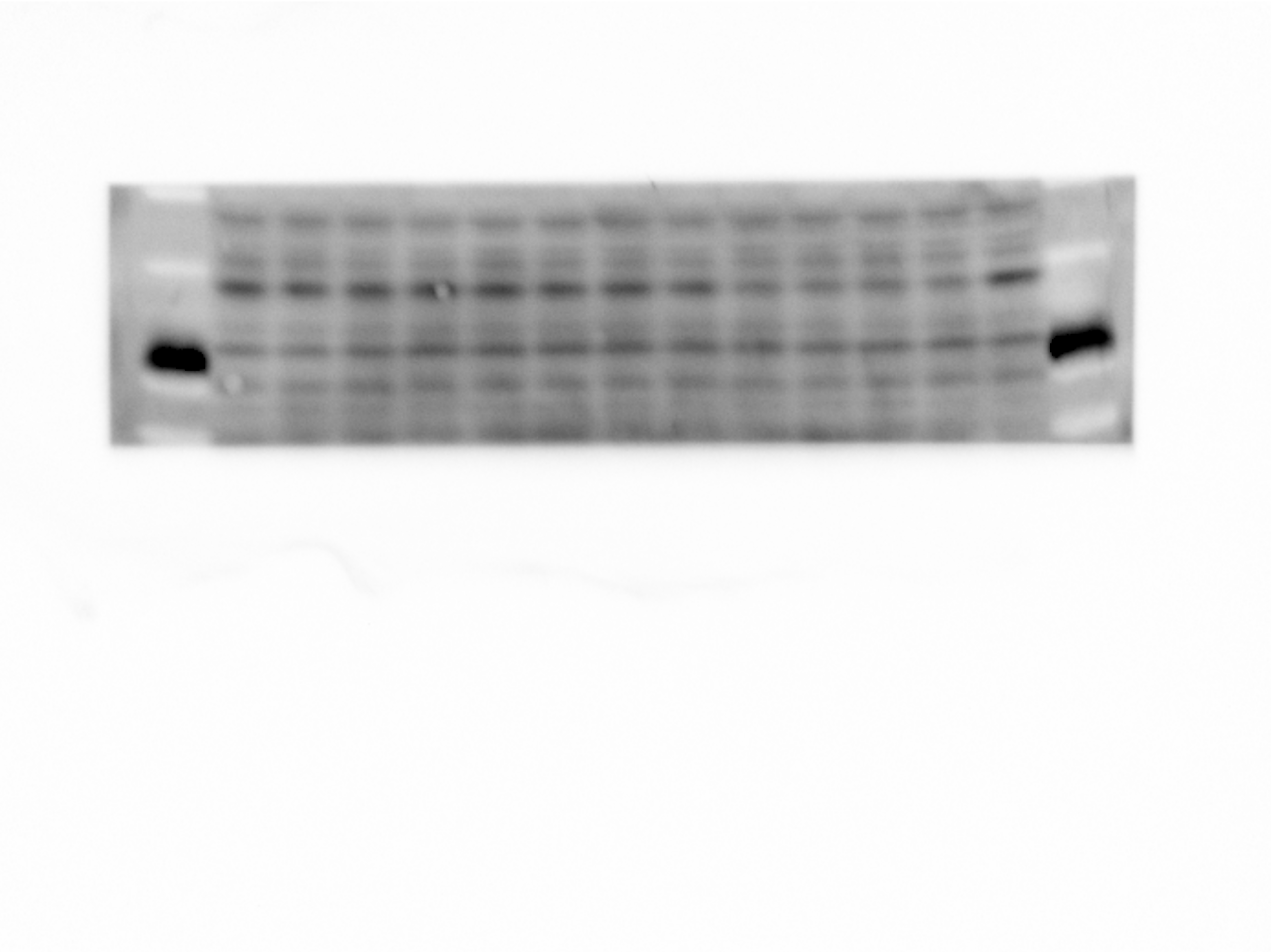

Males - Pdgfra - mb.tif

2551 x 1902

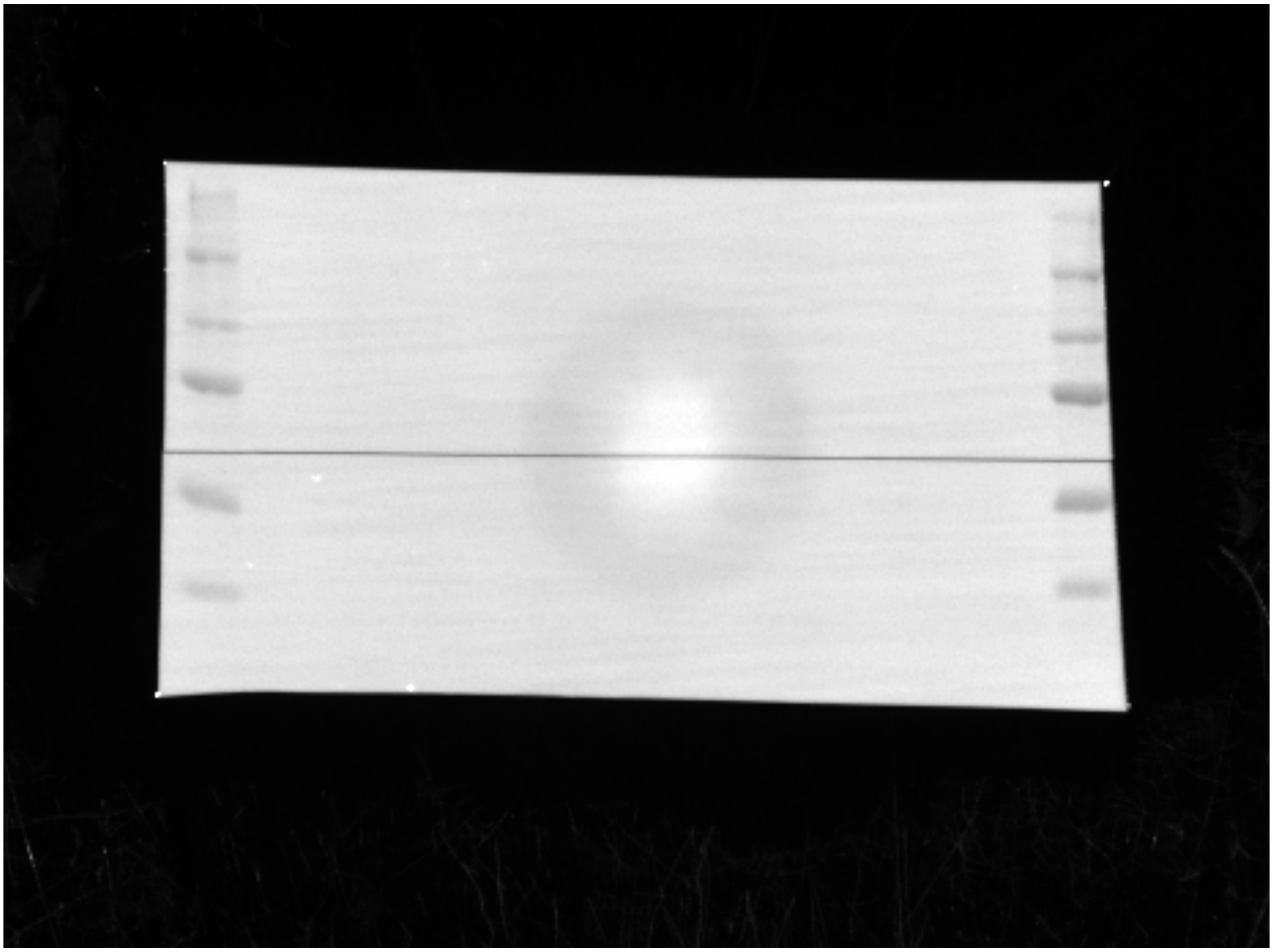

Males - Pdgfra - pt.tif

2103 x 1263

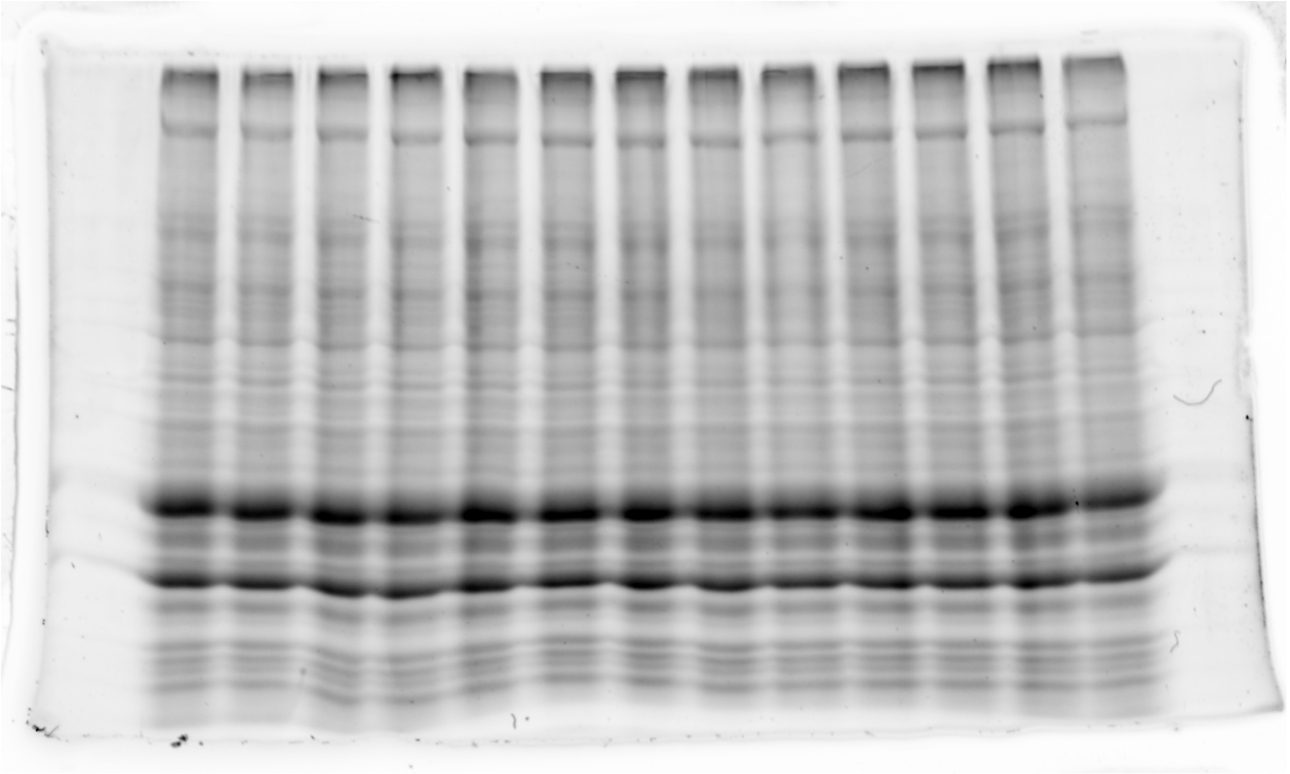

Males - Pdgfra - tp.tif

2551 x 1906

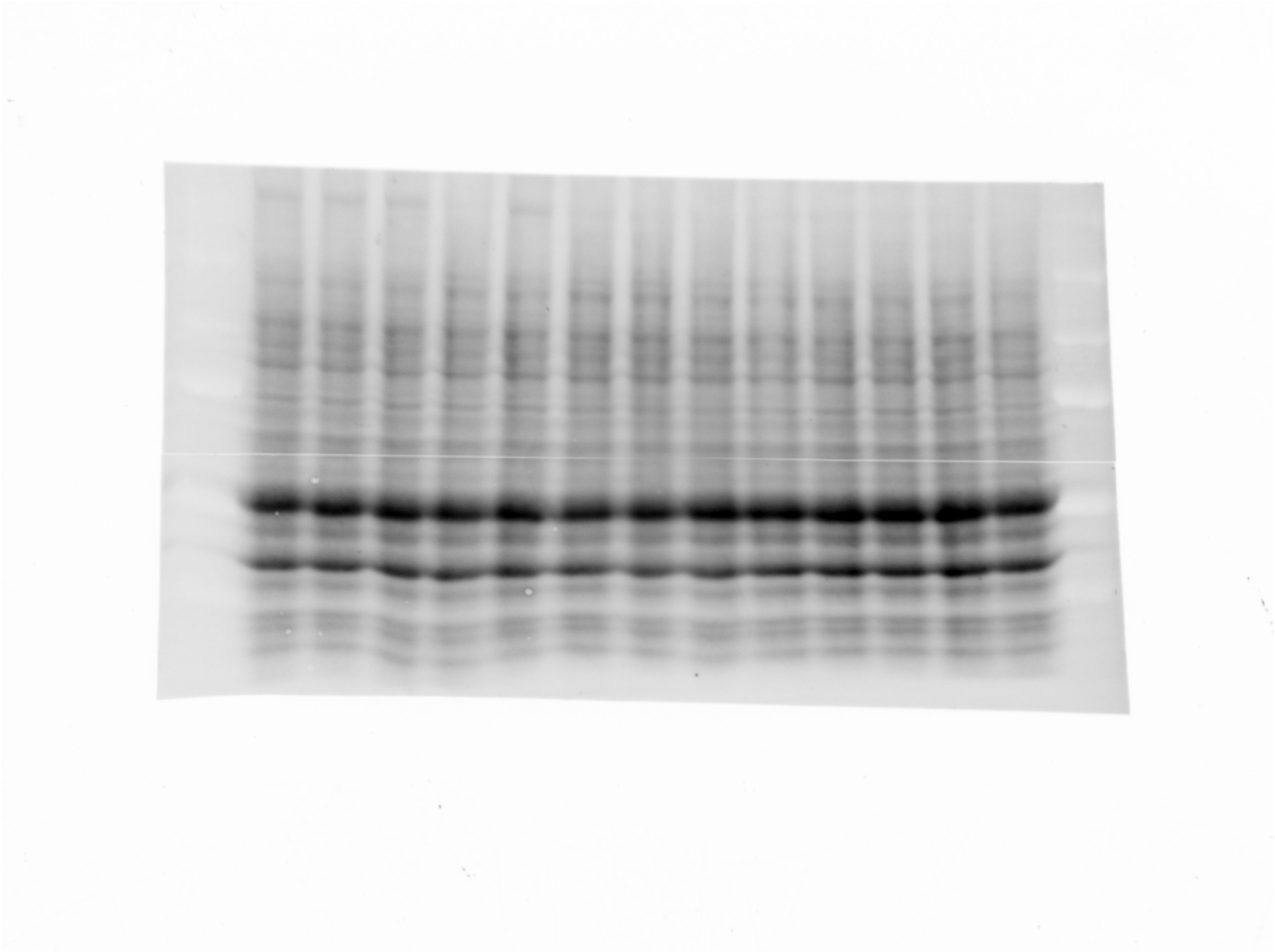

Males - Pdgfra.tif

2551 x 1906

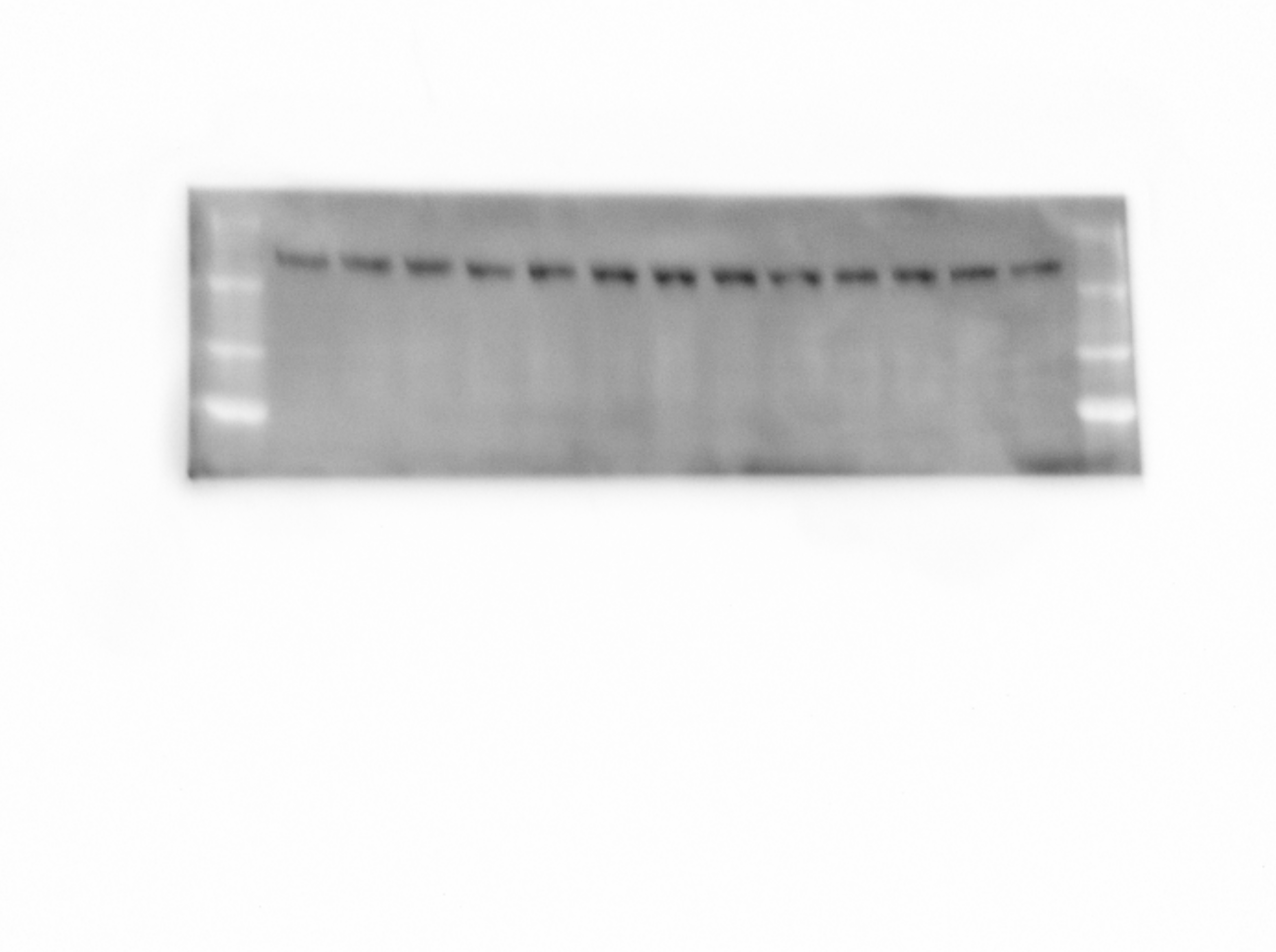

Supplement: awad249_Supplementary_Data [file awad249_supplementary_data.zip › brain-2023-00146-File015.pdf]
