# Supplementary material for: Hypomyelination, hypodontia and craniofacial abnormalities in a Polr3b mouse model of leukodystrophy
Source: Brain. 2023 Aug 28;146(12):5070–85. doi: 10.1093/brain/awad249 (PMC10690025; doi:10.1093/brain/awad249)
Supplement: awad249_Supplementary_Data [file awad249_supplementary_data.zip › Supplementary Materials.pdf]

Supplementary Material for:

Hypomyelination, hypodontia and craniofacial abnormalities in a *Polr3b* mouse model of leukodystrophy

Mackenzie A. Michell-Robinson, Kristin E.N. Watt, Vladimir Grouza, Julia Macintosh, Maxime Pinard, Marius Tuznik, Xiaoru Chen, Lama Darbelli, Chia-Lun Wu, Stefanie Perrier, Daryan Chitsaz, Nonthué A. Uccelli, Hanwen Liu, Timothy C. Cox, Christoph W. Müller, Timothy E. Kennedy, Benoit Coulombe, David A. Rudko, Paul A. Trainor and Geneviève Bernard*

Corresponding Author: Dr. Geneviève Bernard

Email: genevieve.bernard@mcgill.ca

This file contains the following supplementary materials:

1 Supplementary Methods A - G (pp. 2 – 15)
2 Supplementary Figures 1 – 23 (pp. 16 – 50)
3 Supplementary Tables 1 – 3 (pp. 51 – 53)
4 Supplementary Video Legends 1 – 3 (pp. 54)
5 Supplementary References (pp. 55 – 56)

Supplementary Methods

A. Proteomics

i. DNA plasmid construction

POLR3B exon 1-9 fragment was amplified by PCR from the human *POLR3B* cDNA sequence (Dharmacon clone ID: 4905794) using the following primers (Forward: 5’-CTTGGAGCGGCCGCATGGACGTG-3’ and Reverse: 5’-CAACAGATCTATTTAAATGCCTTAAATATGATGACAATGG-3’) and cloned in a p3XFLAG-CMV14 expression plasmid (Sigma) to produce the p3XFLAG-CMV14-*POLR3B* exon 1-9 plasmid. *POLR3B* exon 11-28 fragment was amplified by PCR from the human *POLR3B* cDNA sequence using the following primers (Forward: 5’-CGGTTGATTTAAATATATAGGGAACAAAGTAAG-3’ and Reverse: 5’-CTGAACGGATCCTCTAGATTCATTGTACTT-3’) and cloned in the p3XFLAG-CMV14-*POLR3B* exon 1-9 plasmid to produce a p3XFLAG-CMV14-*POLR3BΔ10* expressing plasmid. The restriction site (SwaI) used for *POLR3B* exon 11-28 cloning introduced a T729A mutation compared to the expected Δ10 sequence (p3XFLAG-CMV14-*POLR3B*Δ10 T729A). The T729A mutation was corrected by site-directed mutagenesis by amplifying the p3XFLAG-CMV14-*POLR3B*Δ10 T729A-expressing plasmid with overlapping primers encoding the desired mutation with a Q5 hot-start high-fidelity DNA polymerase (New England Biolabs) and overnight digestion with DpnI (New England Biolabs) prior to transformation. All *POLR3B* plasmid sequences were verified by sequencing before moving to each subsequent construction step.

ii. FLAG affinity purification

Human embryonic kidney cell line 293 (HEK293T) were maintained in culture in DMEM containing 4.5g/L glucose (ThermoFisher, 11995-065) supplemented with 10% fetal bovine serum (Wisent, 080-150), 2 mM glutamine (ThermoFisher, 25030081), 100 U/mL penicillin and 100 µg/mL streptomycin (ThermoFisher, 15140122). 8.51 µg of p3XFLAG-CMV14-POLR3BΔ10 expression vectors or an empty vector were transiently transfected in 15 cm plates containing $1.6x10^{7}$ cells/plate, grown overnight, by using Jet Prime transfection reagent (PolyPlus) according to manufacturer’s instructions. Transfected cells were incubated at 37°C for 24 hours, harvested, washed with 1X PBS and snap frozen in liquid nitrogen. FLAG affinity purification protocol was modified from Kean et al.[1] Cell membranes were disrupted by a 30-minute incubation at 4°C followed by a liquid nitrogen freeze-thaw cycles in lysis buffer (25 mM HEPES, 1 mM EDTA, 0,05% NP-40, 5% Glycerol, 50 mM KCl, 1 mM DTT, 1X cOmplete EDTA-free Protease Inhibitor Cocktail (Millipore Sigma, COEDTAF-RO), 1 mM PMSF, 10 mM NaF and 1 mM Na3VO4). The protein extracts were cleared of insoluble material by a 30-minute centrifugation at 4°C (16000 x g) and the protein content was assessed by Bradford assay. 30 µL of anti-FLAG (M2) magnetic bead slurry (MilliporeSigma, M8823) were washed four times with 1 mL of lysis buffer, then incubated for three hours with 1.5 mg of total proteins extract at 4°C on a tube rotator. The beads were then washed four times in 1 mL of lysis buffer followed by four more times with 1 mL wash buffer (75 mM KCl, 50 mM ammonium bicarbonate pH 8.0). The bound proteins were eluted by three successive 15-minute incubations in 150 µL of

ammonium hydroxide solution pH 11-12 (roughly 10 %) in HPLC-grade water (Sigma, 7732-18-5). The combined fractions were dried in a speed-vac and then resuspended in 10 µL of 6 M urea. Reduction buffer was added at a volume of 2.5 µL (45 mM DTT, 100 mM ammonium bicarbonate) and the samples were incubated for 30 minutes at 37°C. An additional 2.5 µL of alkylation buffer (100 mM iodoacetamide, 100 mM ammonium bicarbonate) were included to the mix followed by a 20-minute incubation at 24°C in the dark. 20 µL of HPLC-grade water were added to reduce urea concentration and trypsin digestion was performed using a 1:20 (enzyme:protein) ratio of sequencing grade modified trypsin (Promega, V5111) and 18-hour incubation at 37°C on a ThermoMixer (Eppendorf, 5382000023). Following a quick centrifugation (500 x g, 1 minute) the peptides were collected. Trifluoroacetic acid was added to the samples and residual salts and detergents were removed using an Oasis MCX 96-well Elution Plate (Waters, 186001830BA) loaded onto a Positive Pressure-96 Processor (Waters, 186006961) according to the manufacturer's instructions. Eluates were dried down in vacuum centrifuge and then re-solubilized under agitation for 15 minutes in 11 µL of 2% acetonitrile, 1% formic acid, of which 5 µL were used for LC-MS/MS.

iii. Mass spectrometry

High performance liquid chromatography (HPLC) was performed in a 75 µm i.d.x150 mm Self-Pack C18 column installed in the Easy-nLC II system (Proxeon Biosystems). Employed buffers were: 0.2% formic acid (buffer A) and 90% acetonitrile/0.2% formic acid (buffer B). Peptide elution was performed with a two-slope gradient at a flowrate of 260 nL/minute. Solvent B gradually increased from 2 to 37% over the course of 90 minutes and then from 37 to 85% B in 10 minutes. The HPLC system was coupled to an Orbitrap Fusion mass spectrometer (Thermo Scientific) through a Nanospray Flex Ion Source (ThermoFisher, ES071). Nanospray and S-lens voltages were set to 1.3-1.8 kV and 60 V, respectively. Capillary temperature was set to 250°C. Full scan MS survey spectra (m/z 360-1560) in profile mode were acquired in the Orbitrap with a resolution of 120,000 with a target value at $3x10^5$. The 25 most intense peptide ions were fragmented in the HCD collision cell and analyzed in the linear ion trap with a target value at $2x10^4$ and normalized collision energy at 29. Target ions selected for fragmentation were dynamically excluded for 15 seconds after 2 MS/MS events.

iv. Data analysis

Label-Free Quantification (LFQ) intensity for each proteins were obtained by using MaxQuant (version 1.6.0.16) against the characterized human UniProtKB database (release on June 3th 2018).[2,3] Log2 transformation, imputation and further statistical analysis were performed with Perseus (version 1.6.14.0).[4] All purifications were done in triplicate and proteins detected in all experiments were kept for further analysis. Missing values were replaced by randomly generated intensities normally distributed with a width of 0.3 times and a downshift of 1.8 times the standard deviation of non-zero intensities. Significant differences between Log2 protein intensities from bait purifications and the control groups were then determined using a two-tailed T-test subsequently adjusted for multiple hypothesis testing with a permutation-based False Discovery Rate (FDR) of 0.05

and a s0 factor of 0.1 with 10,000 iterations. For all analyses, q-value≤0.05 was considered statistically enriched.

B. Transgenic Mice

Animal husbandry and all studies described in the publication including animal euthanasia adhered to our Animal Care and Use Protocol, as approved by the McGill University Health Center Animal Resource Division (AUP8055).

i. Backcrossing

All parental strains were received at our facility as live breeders except the *Polr3b*^{fl} transgenic strain which was rederived from embryos on a C57BL6J background by the McGill Integrated Core for Animal Modelling (MICAM) Facility. All parental strains were backcrossed at least 6 times to C57BL6J, where two crosses to the background strain alternating male and female transgenic heterozygotes counted as 1 backcross generation (n=1). Founders were received from Dr. William Richardson's group as *Myrf*$^{+/fl}$*; Pdgfrα-Cre/ERT2; R26-Stop*^{fl}*-EYFP* mice (Supplementary Figure 1B) which were subsequently backcrossed to C57BL6J mice in order to isolate the *R26-Stop-EYFP* ("*YFP*") parental strain. NB. We used *Pdgfrα*-Cre/ERT ("*Pdgfrα-CreERT*") and not the Cre/ERT2 strain from Dr. Richardson's lab in this study.

ii. Breeding

Breeding was carried out as follows, where P represents the fully backcrossed heterozygous parental strain (*i.e.,* N?+N6 according to Jax breeding nomenclature):

Cross 1:
Pdgfrα-CreERT x (P) *Polr3b*$^{+/fl}$ (P) → *Pdgfrα-CreERT; Polr3b*$^{+/fl}$ - 25%

Cross 2:
Pdgfrα-CreERT; Polr3b$^{+/fl}$ x *YFP* (P) →
Pdgfrα-CreERT; Polr3b$^{+/fl}$*; YFP* - 12.5%

Cross 3:
Polr3b$^{+/fl}$ (P) x *Polr3b*$^{+/fl}$ (P) → *Polr3b*$^{fl/fl}$ - 25%

Cross 4: *Pdgfrα-CreERT; Polr3b*$^{+/fl}$*; YFP* x *Polr3b*$^{fl/fl}$ →

Pdgfrα-CreERT; Polr3b$^{+/fl}$*; YFP*	- 12.5%
Pdgfrα-CreERT; Polr3b$^{fl/fl}$*; YFP*	- 12.5%
Polr3b$^{+/fl}$*; YFP*	- 12.5%
Polr3b$^{fl/fl}$*; YFP*	- 12.5%
Pdgfrα-CreER$^{+/cre}$*; Polr3b*$^{+/fl}$	- 12.5%
Pdgfrα-CreER$^{+/cre}$*; Polr3b*$^{fl/fl}$	- 12.5%
Polr3b$^{+/fl}$	- 12.5%
Polr3b$^{fl/fl}$	- 12.5%

iii. Tamoxifen Administration

Tamoxifen was prepared in corn oil at 20 mg/mL and dissolved at 55°C for 3 hours on a heating block. The product was sterile filtered (0.45 um) and stored as 1 mL aliquots at 4°C for no more than one month. Prior to injections, tamoxifen aliquots were re-heated at 55°C for 15 minutes on a heating block, taking care not to perform injections until the solution was cooled to room temperature. Tamoxifen solution was loaded into a 100 uL gas-tight syringe (Hamilton) using a 26-gauge needle and the needle was swapped to a 36-gauge needle to perform injections. Mice were weighed prior to injections. Standard weight at P2 was approximately 1.5-2 g, and 3.5-4 g at P5. Mice were injected with 1 uL (20 µg) of prepared tamoxifen solution for every half gram of weight rounded to the nearest half gram (40 mg/kg). Injections were performed in the evening at 7 PM once daily for four days (P2-P5 inclusive). Average total drug exposure was approximately 400 µg tamoxifen delivered over 4 days.

iv. Genotyping

Genotyping was performed using the AccuStart II genotyping kit (QuantaBio) according to manufacturer's protocols. All genotyping PCRs were run at 95°C for 5 minutes (denaturation) to start, then 30 cycles at 95°C for 30 seconds, 58°C for 40 seconds, 72°C for 60 seconds, followed by 72°C for 7 minutes (termination) and a hold at 4°C. Collection of samples for genotyping was performed on P2, prior to injection of tamoxifen. Pups were identified by tattooing. Pups with homozygous *YFP* expression or germ-line recombination at the *Polr3b* locus were screened out of subsequent studies. Primers and PCR reaction parameters for $Myrf^{fl}$ are not shown as we did not maintain the line for further studies but were identical to those used in prior publications by Dr. Richardson's group.[5]

v. Genotyping Primers

Polr3b Genotyping:
Forward: AGCCTCATTTCTTCGTGGTGA
Reverse: TGGCCTAGGAAGGGAAGAAAA

Polr3b Recombination:
Forward: AGCCTCATTTCTTCGTGGTGA
Reverse: CCCTGGAATGAACAGGGGAAA

Pdgfrα-CreERT Genotyping:
Forward: CAGGTCTCAGGAGCTATGTCCAATTTACTGAACGTA
Reverse: GGTGTTATAAGCAATCCCCAGAA

YFP Genotyping:
Forward 1: AAAGTCGCTCTGAGTTGTTAT
Forward 2: GCGAAGAGTTTGTCCTCAACC
Reverse: GGAGCGGGAGAAATGGATATG

vi. Reverse-Transcription – Polymerase Chain Reaction (RT-PCR)

Total RNA was isolated from fresh P30 brain tissue by homogenizing the whole brain with a razor blade on a glass slide on top of wet ice. Homogenized tissues were snap frozen in liquid nitrogen and stored at -80°C until use. Brain tissue was washed twice in ice cold PBS and mixed with a glass rod in Qiazol (Qiagen) on wet ice, then triturated through a 1000 uL pipet tip until a clear solution was obtained. Total RNA was extracted on columns using the manufacturer's protocol with an amended 30-minute DNAse treatment (miRNeasy, Qiagen). cDNA was reverse transcribed from total RNA using Superscript IV according to the manufacturer's protocol (ThermoFisher). The subsequent PCR reaction used Qiagen Taq polymerase and was carried out in a 50 uL reaction according to the manufacturer's protocol using the following primers:

Forward (exon 8/9 boundary): CTCAGTTACCAGCTCCACCC
Reverse (exon 14): GGCCACTCACTTTCCTCGTC

C. Tissue Histology

i. Tissue Blocks

Tissue blocks were prepared as outlined in the manuscript.

ii. Hematoxylin and Eosin (H&E)

H&E staining was carried out on 10 μm FFPE sections prepared as outlined in the manuscript, using the Abcam H&E staining kit (ab245880). Staining of FFPE sections with Hematoxylin and Eosin was carried out using the manufacturer's protocol including washes in absolute ethanol and differentiation for 2 minutes in bluing reagent.

iii. Luxol fast Blue (LFB)

LFB staining was carried out using the Abcam LFB Stain kit (ab150675), as directed by the manufacturer. In short, 15 μm FF sagittal sections were incubated overnight with LFB solution. Sections were differentiated in lithium carbonate 0.05%, followed by alcohol 70%. After dehydrating with ethanol 100%, sections were cleared and mounted with Permount™ mounting medium (Thermo Fisher Scientific).

iv. Immunofluorescence

Immunofluorescence was carried out on 15 μm FF sections prepared as outlined in the manuscript. Staining was performed by equilibrating sections for 30 minutes at room temperature, blocking in 5% FBS with 0.2% Triton-X100 in PBS for one hour, followed by incubation with primary antibody overnight at room temperature, followed by three washes in 0.2% Triton-X100 in PBS, followed by incubation with secondary antibody for 3 hours at RT with DAPI, followed by 3 washes in PBS and a final wash in double distilled water. Sections were mounted using Shandon Immumount (ThermoScientific) and stored at 4°C. Images were acquired within 24 hours of preparing slides. Primary antibodies raised against Mbp (Novus Biologicals NB600-717), Olig2 (Abcam ab136253), Ng2 (Millipore AB5320), Pdgfrα

(Cell Signalling D1E1E), Apc “CC1” (Millipore OP80), Cleaved Caspase 3 (Cell Signalling 9661), or YFP (Nacalai Tesque GF090R) were used. Secondary antibodies used were Donkey Anti-Rat Alexa 488, Donkey Anti-Rabbit 546 or Donkey Anti-Mouse 594, Donkey Anti-Goat 680 (Thermo).

v. 5-ethynyl-2'-deoxyuridine (EdU) Labelling

Mice were exposed to EdU prepared according to manufacturer’s recommendations in phosphate buffered saline at 1 mg/mL. Mice were exposed to EdU by administration of two intraperitoneal injections separated by three hours. The total administration of EdU was 100 ug (50 uL per injection), and the total labelling time was 6 hours, three hours following each injection. Mice were subsequently euthanized at the 6-hour time point.

vi. Imaging

Images of hematoxylin and eosin stained FFPE sections were acquired on a Leica Aperio AT Turbo digital whole slide scanning system at 20x resolution. Fluorescence images were acquired using a Zeiss Axioscan Z1 slide scanner employing a plan-apochromat 10x objective and AxioCam MR R3 camera. Imaging parameters were adjusted such that histograms were non-saturating and focus was automatically adjusted using the onion setting. Imaging settings were maintained, and all slides were imaged in a single batch with the exception of Ng2 stained slides which were imaged in two age-grouped batches with identical settings. Photomicrographs of LFB stained sections were acquired on a Zeiss Axio Observer.Z1 microscope with a 10x objective. All sections were imaged using a Axiocam 506 camera, in a single session and maintaining the imaging parameters. Images were processed with ImageJ (NIH) software. A threshold was set up for each area (corpus callosum and cerebellum), and the relative stained area was quantified.

D. Electrophoresis and Immunoblotting

i. Tissue Dissection

Mice underwent euthanasia according to Animal Care and Use Protocol procedures (isoflurane and CO_2). Animals were decapitated, cranial skin removed, and frontal, parietal, and occipital skull bones were dissected away using dissection scissors and forceps. The brains were removed from the skull gently and placed on a histology slide on top of wet ice. The cerebellum was gently pulled away posteriorly from the midbrain structures, and forceps were used to cut away the cerebellar and pontine tissues. The olfactory bulbs were similarly removed using forceps. The midbrain and forebrain structures were chopped into a fine homogenate on the cold slide using a razorblade and approximately half the tissue was loaded into each of two 1.5 mL centrifuge tubes. Immediately after preparing the tissue and loading the centrifuge tubes, the tubes were dropped into liquid nitrogen. The tubes were subsequently stored at -80°C until use.

ii. Sample Preparation

Tissues were recovered from -80°C storage and equilibrated to 0°C on wet ice. Tissue was weighed (aliquots were approximately 150 mg on average). Fresh protease inhibitors were added to RIPA buffer (Thermo), and RIPA buffer was added to a final concentration of 100 mg of tissue per mL of buffer in disposable 13 mL round bottom tubes. Tissue was mechanically homogenized using a Dounce homogenizer rod in the disposable tube and centrifuged briefly at 4°C to remove bubbles. Lysates were left to sit on ice for 15 minutes before being mechanically dissociated twice more by repeated trituration through a 1000 µL pipet tip, each trituration step followed by 10 minutes of rest on wet ice. Samples were finally sonicated using a tip sonicator for 1.5 minutes total in cycles of 10-second sonication increments at 30% amplitude with 10 seconds of rest on wet ice. After sonication, lysates were resuspended in Laemmli buffer (BioRad) with fresh reducing agent (beta-mercaptoethanol) added. Samples were heated for 10 minutes at 70°C and cooled on wet ice, then stored at -20°C until use.

iii. Protein Quantitation

Protein standards were prepared from wildtype C57BL6J brains (P28) by precipitating proteins from the aqueous phase of a Bligh and Dyer extraction, which was carried out primarily to discard lipids in order to measure the dry protein weight. Briefly, brains were dissected, and tissue lysates prepared in RIPA buffer as described above. 1 volume of the resulting lysate was resuspended in a final volume consisting of 0.8 volumes of ddH2O, 2 volumes of methanol, and 2 volumes of chloroform. The solution was vortexed vigorously (3 times for 10 seconds, each followed by a 10-second rest on ice), and centrifuged at 4000 RPM for 10 minutes at 4°C. The aqueous phase containing protein was recovered by pipetting into a new tube, and 4 volumes of methanol was added to precipitate the proteins, followed by vortexing and centrifugation as described in the previous step. The resulting pellet was subsequently washed in 4 volumes of methanol, vortexed, and centrifuged once more. The supernatant was discarded, and the pellet was dried in a Speedvac at 25 bar pressure with no heat. The pellet was weighed and resuspended in RIPA buffer with protease inhibitors and sonicated, followed by resuspension in Laemmli buffer and further sonication as described above. The final concentration of the resuspended protein standards was 20 mg/mL. The standards were heated at 70°C for 10 minutes and stored at -20°C.

Protein standards were resuspended in Laemmli buffer at different concentrations in order to prepare an 8-point dilution curve from 10 mg to 0 mg in ½ dilution increments. 1 µL of each of the resulting standards was pipetted onto dry nitrocellulose membrane in triplicate. 1 µL of each sample (diluted 1:5 in Laemmli buffer) was pipetted onto the same membrane in duplicate. The membrane was allowed to dry at room temperature for 10 minutes and then washed twice in ddH2O. The membrane was then incubated in 4% Trichloroethanol (TCE) in ddH2O at room temperature and then placed on the stage of the Chemidoc MP (Biorad), with the buffer layered on top. The membrane was activated for 5 minutes under ultraviolet light using the manufacturer's Stain-Free gel activation protocol, and then imaged. The resulting dot blot image was analyzed in ImageLab (BioRad) to acquire background-corrected pixel intensity values for each dot. Sample intensity values were used to interpolate sample concentration from a standard

curve created by linear regression to the average protein standard pixel intensity values (Graphpad Prism 9.4.1). Samples were normalized to 1 mg/mL in Laemmli buffer after protein concentrations were obtained.

iv. Electrophoresis

SDS-PAGE was carried out according to standard protocols with minor modifications. Tris-Glycine polyacrylamide gels were prepared according to the original Laemmli protocol with 0.075% APS and TEMED used to initiate resolving gel polymerization, and 0.075% APS and 0.15% TEMED used to initiate stacking gel polymerization. Additionally, 0.5% v/v TCE was added to the resolving gel mixture prior to initiating gel polymerization. Gel thickness was tailored to the size of the target protein to facilitate recovery during protein transfer, with 0.75 mm gels used for proteins over 150 kDa (Pdgfrα, Ng2), 1 mm gels used for proteins in the 100-150 kDa range (Polr3a, Polr3b), and 1.5 mm gels used for proteins <100 kDa. Polyacrylamide percentage also varied according to the size of the protein target; we used 8% gels for most targets, except for samples probed for Mbp and Olig2 which were run on 10% gels. Typically, Mbp requires higher percentage gels (12-15%) to resolve its isoforms from one another; in this case we quantified Mbp in aggregate from a single band resolved on 10% gels. The antibody was previously validated by immunoblotting membranes where SDS-PAGE was performed with 15% gels and we did not observe differences in isoform recovery among genotypes of *Polr3bΔ10* mice used in the study, rather, uniform decreases in Mbp recovery were observed in samples from +/Δ10 and Δ10/Δ10 mice relative to CTRLs (not shown). Electrophoresis was carried out at 100 volts, typically for 3-3.5 hours, using standard Tris-Glycine running buffers and a BioRad Mini Protean Tetra Cell with an ice pack in the lower reservoir. Gels were subsequently removed from the glass spacing plates and TCE labelling of proteins was activated under UV light on the Chemidoc MP (BioRad), using the manufacturer's 1-minute Stain-Free gel activation protocol. Gels were then immediately transferred to PVDF support.

v. Protein Transfer

Semi-dry protein transfer was carried out using the TransBlot Turbo and ready-to-assemble kit reagents (BioRad). Briefly, proprietary transfer buffer was prepared with ethanol according to the manufacturer's recommendations, and the transfer sandwich was assembled while soaked in buffer including the gel which was allowed to equilibrate in the buffer for approximately 5 minutes. BioRad low fluorescence PVDF was substituted for Immobilon-P low fluorescence PDVF membranes, which were activated in 100% methanol at RT with gentle nutation until translucent prior to sandwich assembly. The sandwich was assembled and placed into the transfer cassette, taking care to remove all air bubbles. Protein transfer was carried out using the manufacturer's pre-programmed 10-minute, 25-volt protocol for 1.5 mm gels, regardless of the gel thickness. PVDF membranes were then extracted from the apparatus and left to dry on fresh Whatman paper for 15 minutes. Dried PVDF membranes were re-equilibrated for 30 minutes in 100% methanol, followed by three washes in double distilled water and left submerged in Tris-Buffered Saline with 0.1% Tween (TBST-0.1%) while blocking reagents were prepared.

vi. Immunoblotting

Blocking was carried out overnight at 4°C on a rocking platform using 4% non-fat dry milk resuspended in TBST-0.1%. In the morning, membranes were washed twice in TBST-0.1% for ten minutes each at 4°C and left in fresh TBST-0.1% until the end of the day. Primary antibodies were resuspended in 4% Bovine Serum Albumin in TBST-0.1%, except the primary antibody raised against Ng2, which was resuspended in 4% non-fat dry milk in TBST-0.1%. Primary antibody incubation was carried out overnight at 4°C. Following primary antibody incubation, membranes were first washed three times in TBST-0.1% for 10 minutes each at RT, and a further three times in TBST-0.05% for 10 minutes each at RT. Secondary antibodies were resuspended in 4% non-fat dry milk in TBST-0.1%, and membranes were incubated in the secondary antibody solution for 2 hours at RT. Six 10-minute washes were performed following the incubation with secondary antibody as were performed following the incubation with primary antibody. An additional two washes in Tris-buffered saline with no tween, each for 5 to 10 minutes, were performed while ECL reagents were prepared.

vii. Antibodies

The antibodies and diluents used for immunoblotting are outlined in **Supplementary Table 1.**

viii. Imaging

Imaging was carried out on the Chemidoc MP (BioRad) as follows. First, membranes were placed on the imaging stage and imaged under UV light using the Stain-Free manufacturer’s settings with approximately 2 seconds of exposure to acquire the total protein image. The membrane was then removed from the stage and incubated in Clarity Max ECL reagent and allowed to develop for 1-3 minutes depending on the target, before the membrane was placed back on the imaging stage to acquire the chemiluminescent signal image. A third image of the protein ladder was also acquired (Precision Plus – All Blue, BioRad).

ix. Analysis

Western blot images were analyzed in ImageLab (BioRad) using total protein normalization methodology.[6-8] Background-corrected integrated pixel intensities were obtained for each lane from the total protein image, as were values for individual protein bands from the chemiluminescence images. Integrated Intensity values for protein target bands were first divided by the total protein values in each lane (*i.e.,* samples were first normalized to the total amount of protein detected in the lane). Then, the average normalized intensity value was acquired from lanes run with control samples. All normalized intensity values were subsequently divided by this average (*i.e.,* all samples were normalized to the average of the controls). This method produces fold-change values, where the average expression among controls is 1. For each blot, 15 lanes were present including four individual samples of each genotype of the same sex (CTRL, +/Δ10, Δ10/Δ10), one age and sex matched *Shiverer* sample as a negative control for Mbp expression, and two ladder lanes. Each blot included in the analysis was done

once for each sex (n=4 males, n=4 females for each genotype included in the statistical analysis).

x. Nuclear/Cytosolic Western Blot

40 µg of cytoplasm extract and 26 µg of nucleus extract were loaded as indicated in Figure 1. Cell extracts were obtained from HEK293T cells transiently transfected with either *POLR3B* WT (WT) or the Δ10 mutant expressing plasmid and harvested 24 hours post transfection. p3XFLAG transfected cells were used as a negative control (Mock). Shown in Figure 1 (main manuscript) is a representative immunoblot performed from three independent experiments. 3XFLAG-POLR3B WT or 3XFLAG-POLR3BΔ10 expression was assessed by western blot using an anti-FLAG primary antibody (1:2000) with an exposure time of 5 seconds or 30 seconds. GAPDH (1:1000) and Lamin A+C (1:2000) were used as loading control for the cytoplasm and the nucleus respectively. Although they are presented separately, the nuclear and cytoplasmic extracts were run simultaneously on the same gels/blots.

E. Magnetic Resonance Imaging

i. Animals

Four animals from each genotype (2 males, 2 females) were used in the study comprising animals from four litters. Δ10/Δ10s, +/Δ10s, and CTRLs were grouped by litter, whereas tamoxifen negative controls were taken from a separate, untreated litter. CTRLs (Cre negative controls) consisted of three *Polr3b*$^{+/fl}$ mice, two of which also carried an *YFP* allele. The fourth CTRL mouse had a *Polr3b*$^{fl/fl}$ genotype. All animals used in the MR imaging studies are detailed below in **Supplementary Table 2**.

ii. Tissue Preparation

Mice underwent trans-cardiac perfusion under isoflurane anaesthesia with PBS followed by freshly prepared 4% paraformaldehyde. Brains were extracted by dissection as detailed in the manuscript and were subsequently post-fixed in 4% paraformaldehyde for 24 hours at 4°C. Brains were then transferred to fresh PBS with 0.05% sodium azide and stored at 4°C until use. Brains were transferred to the McConnell Brain Imaging Centre on ice and subsequently stored locally at 4°C until scans were performed.

F. Cell Counting

i. Animals

Animals used in the cell counting study came from at least two litters including at least one animal of each genotype (CTRL, +/Δ10s, Δ10/Δ10s). Additional animals included in the analysis were usually but not always littermates and were added according to the availability of tissue (**Supplementary Table 3**).

ii. Tissue preparation

Tissue preparation was done as outlined in the manuscript. For each analysis, tissue from littermates (all three genotype groups) were arranged on the same

slide, up to a maximum of five sections including additional n's from the same or other litters. Slides were stained simultaneously for all studies except Ng2 stained slides which were prepared and imaged together, but at an earlier date.

iii. Staining

Staining was carried out as outlined in Supplementary Methods C. Antibody panel 1 was used to assess DAPI, YFP (Alexa 488), CC1 (Alexa 594) and Pdgfrα (Alexa 647) labelled cells. Antibody panel 2 was used to assess DAPI, YFP (Alexa 488) and Ng2 (Alexa 647) labelled cells. Antibody panel 3 was used to assess DAPI, YFP (Alexa 488), Olig2 (Alexa 546) and Edu-647 labelled cells. Antibody panel 4 used in the study included DAPI, YFP (Alexa 488) and Cleaved Caspase 3 (Alexa 647), but cells were not quantified due to low and similar labelling index across all samples.

iv. Image Export

Histological images were exported from Zeiss proprietary format to full depth 16-bit .tiff images in FIJI (Fiji is Just ImageJ) using the Bioformats Import tool. Binary masks were created from manually delineated regions of interest and included the Medulla, Pons, Cerebellum, Midbrain, Thalamus, Hypothalamus, Anterior Commissure, Corpus Callosum, and Cortex/Striatum and whole brain. All files were stored on an external hard disk, and copies were uploaded to Amazon S3 storage services using the PuTTY secure copy command line client.

v. Image Processing Pipelines

Image processing pipelines were developed in CellProfiler 4.2.1 on a local machine using representative images from each dataset.[9-13] The imaging pipeline used in the analysis calculates an illumination function with which it performs background correction of the original image, followed by isolation of the brain section from the slide background using a manually delineated mask. Nuclei are detected using an automatic global threshold (Robust Background method) from the Dapi channel image. Nuclear objects are then filtered according to a set size (equivalent diameter, major axis length, area). Secondary objects (cell features, e.g., YFP+ cytoplasm) in the green or red channel are detected based on the nuclear object segmentation according to a propagation algorithm which appends pixels from a globally thresholded image where the brightest 5% of pixels (>2 standard deviations above the mean) are selected for propagation. An array of over 250 object-based measurements is made for each object in each category (nuclear, secondary green, secondary red) including size, shape, intensity, and intensity distribution measurements which are outputted as values in a database unique to each image in SqLite format.

vi. Object-Based Measurements

Measurements of objects in images stored on Amazon S3 were obtained using r6a.48xlarge machines (Amazon EC2) running Distributed CellProfiler 2.0.0_4.1.3 (https://github.com/DistributedScience/Distributed-CellProfiler). Distributed CellProfiler is analogous to running CellProfiler on a local machine but allows for parallel computing wherein individual images are analyzed on separate machines

in the cloud. The imaging pipelines developed as described above were uploaded and used to analyze the images. Object measurements outputted in SQLIte format were uploaded to Amazon S3 and subsequently collated together.

vii. Data Collation

Individual SQLite databases for each image were collated together for each experiment so that sections from all animals in an experiment would be analyzed together. Collating the databases together involved merging the databases while preserving unique identifiers mapping to each image. Python code was written for this purpose which functions in the context of PyPy 3 or Python 3 with the appropriate libraries and packages included and was uploaded to GitHub along with documentation (https://github.com/mamrrmam). Briefly, the program takes a list of SQLite databases and merges them, while performing some basic manipulations to ensure that data from each image is stored correctly and is traceable back to the original image. The program also performs some quality control to ensure the databases contain the same tables and notifies the user if errors arise. Finally, the data is post-processed to ensure that the merged database interfaces correctly with CellProfiler Analyst. For example, post-processing involves redefining primary keys and foreign keys such that objects and images are referenceable to one another in the relational database and are therefore accessible to CellProfiler Analyst.

viii. Classification

Collated databases were subsequently downloaded and analyzed in CellProfiler Analyst to produce separate, automated classification models for the green and red channels, using randomly selected training images from each database. Training images of individual cells are produced in CellProfiler Analyst by cropping the area around individual objects. Training images were iteratively added to the training set until at least 50 images of marker positive cells were obtained. Classifiers in individual channels obtained higher than 90% accuracy on training sets. Unclassifiable cells were screened out using a specific category which identified and removed cell objects detected in meninges, choroid plexus, or along tissue edges or in areas that otherwise had less well-defined focus. Cell counts for each classification category were processed using CellProfiler Analyst and written to the individual databases, at which point results were queried from the databases directly. Classification produced consistent total cell counts per unit area and similar numbers of unclassifiable cells for all sections.

ix. Data Analysis

Cell counts were used to calculate tissue density in mm2 using the three-step approach outlined below:

$$Cell\ Density = \frac{Cell\ Count}{Tissue\ Area\ (mm^2)} \quad (1)$$

$$Density\ Normalization\ Factor = \frac{Cell\ Density}{Group\ Average\ Cell\ Density} \quad (2)$$

$$Normalized\ Cell\ Density = \frac{Cell\ Density\ (1)}{Density\ Normalization\ Factor\ (2)} \quad (3)$$

Values were plotted and data analyzed by Two-Way ANOVA with multiple comparisons evaluated according to the Tukey method with α=0.05. Comparisons between two groups at an individual time point were made by Welch's two-tailed t-test with α=0.05. Data were presented as the mean of biological replicates with standard deviation.

G. Study Design and Statistical Analysis

i. Study Design

Studies were designed to minimize the total number of animals used. Sample sizes were decided a priori based on prior work, consensus among colleagues, and in line with Animal Care and Use Protocol (AUP 8055) specifications. All litters containing at least one animal with an appropriate genotype (ie. *Pdgfra-CreERT*; *Polr3b*$^{fl/fl}$ with or without *YFP* (as required) were included in the experimental pipeline. Entire litters were injected with tamoxifen in all such cases. When there were excess non-homozygous animals (*e.g.,* +/Δ10, CTRL) in a litter, mice were chosen randomly for downstream tissue collection. Randomization was done by selecting animals by their animal number after genotyping, allocating one or more animals (per genotype group) as required. Other environmental/experimental confounders are not anticipated to have played a major role in animal development and were minimized by randomizing the order in which animals were manipulated. Blinding was maintained throughout sample analysis using a numbering system to collect and store samples but could not be reliably done during behavioural assessment of live animals due to obvious physical differences between Δ10/Δ10s and +/Δ10 or CTRLs. Analysis included samples from all animals initially included in the experimental pipeline that were euthanized for tissue collection at an appropriate time point. Animals/samples that were excluded from statistical analysis are indicated in the final graphs using a special symbol "*" if the values fell more than 2 standard deviations away of the mean of their experimental group *and* there was rationale for removing the value from the analysis (*e.g.,* suspected poor measurement).

ii. Statistical Analysis

Biological replicates (*i.e.,* individual mice) are presented for all measurements in all figures, represented as individual points in their respective graphs with group mean and standard deviation indicated unless otherwise stated. Parametric statistical analysis validity was evaluated by testing data with normality/lognormality tests (*e.g.,* Shapiro-Wilk Test) and by evaluating QQ plot linearity. Where possible, statistical analyses that did not assume the comparison groups had equal variances were used (*e.g.,* Brown-Forsythe and Welch's tests). Comparisons between 2 groups was done using unpaired two-tailed Welch's t-tests (α=0.05). Statistical analysis between 3 groups at an individual time point were done by unpaired Brown-Forsythe and Welch's One-Way Analysis of Variance (ANOVA) with group means compared to each other and significance tested post-hoc adjusting for multiple comparisons by the Dunnett T3 method (α=0.05). Cell count time series were analyzed by ordinary two-way ANOVA with all genotype group means compared to each other at each time point, and

significance tested post-hoc adjusting for multiple comparisons by the Tukey method (α=0.05). Statistical analyses were performed with GraphPad Prism 9.4.1, except for statistics on hypodontia which were performed in SPSS v28.0.1.1 (IBM) as detailed in Table 1 (main manuscript).

Supplementary Figures

Supplementary Figure 1: Recapitulating Pdgfrα-dependent *Polr3b∆10* expression during oligodendrocyte development in vivo. (A) Tamoxifen dosing trials were conducted to determine a tolerable dosing strategy. Injections were delivered subcutaneously once daily in the evening. P2-P5 was selected as the best dosing strategy because the dosing schedule starting 1 day earlier (P1-4) and injection protocols delivering an additional injection (P1-5, P2-6) were associated with increased toxicity in WT mice. **(B)** Recombination efficiency was assessed in *Pdgfrα-CreERT*; *YFP* mice induced with tamoxifen using the 40 mg/kg, P2-5 protocol described above. 50 μm spinal cord, cerebellum, or coronal sections at the level of the corpus callosum were prepared on a vibratome and stained as free-floating sections using Anti-Olig2 (1:200) and Anti-YFP (1:1000). Cells were counted and the proportion of double-positive cells divided by the total number of cells positive for Olig2 were used to represent recombination efficiency in the structures indicated. **(C)** RT-PCR was performed using mRNA prepared from P30 brain tissue in 2 Cre negative controls and Δ10 heterozygotes induced with tamoxifen from P2-5 using the standard protocol in order to compare the abundance of WT and Δ10 transcripts in whole brain tissue. Sanger sequencing confirmed the integrity of the exon 9-11 boundary in the mutant transcript. Pictured in the top panel are duplicate lanes run from each individual with the low molecular weight product present only in ∆10 heterozygotes, which was used to sequence the ∆10 transcript. The middle panel are lanes run using the no reverse transcriptase control samples, which were prepared simultaneously but without including the RT enzyme in the RT reaction mix. The lowest panel demonstrates housekeeping control lanes for each individual sample, demonstrating equivalent cDNA content of the RT product (Hprt). **(D)** Founder line genotyping results showed expected results including the *Polr3b*^{fl} allele, the *Pdgfrα-CreERT* allele, and the *YFP* allele. **(E)** Representative recombination in genomic DNA isolated from brain tissue of *Pdgfrα-CreERT;Polr3b*$^{fl/fl}$ mouse and a Cre negative littermate showed the appropriate genotyping PCR bands as well as recombination at the *Polr3b*^{fl} locus. **(F)** Figure legend for the illustration in E.

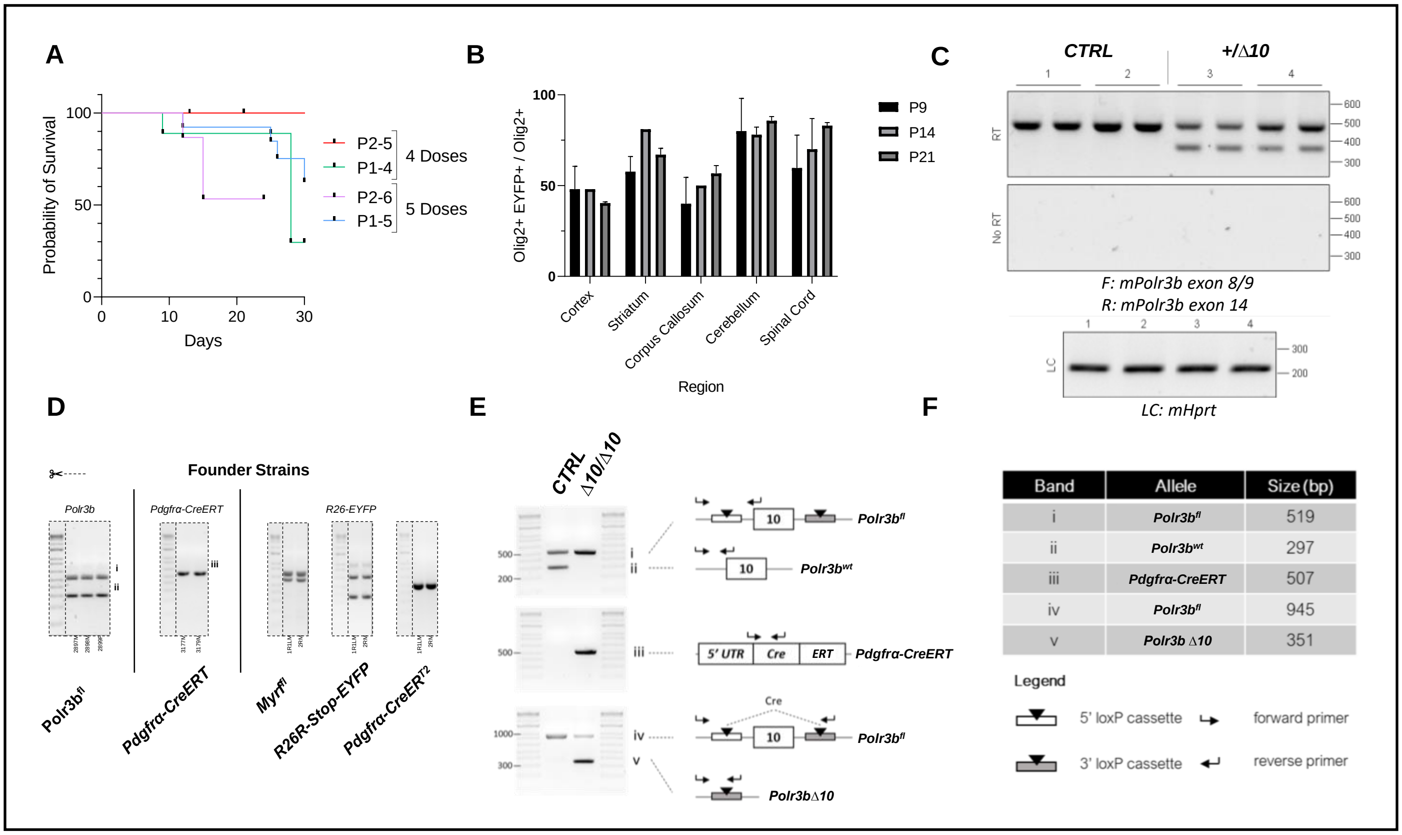
A
Probability of Survival
Days
P2-5
P1-4
4 Doses
P2-6
P1-5
5 Doses
B
Olig2+ EYFP+ / Olig2+
P9
P14
P21
Cortex
Striatum
Corpus Callosum
Cerebellum
Spinal Cord
Region
C
CTRL
+/Δ10
RT
No RT
F: mPolr3b exon 8/9
R: mPolr3b exon 14
LC
LC: mHprt
D
Founder Strains
Polr3b
Pdgfra-CreERT
R26-EYFP
Polr3b^fl
Pdgfra-CreERT
Myrf^fl
R26R-Stop-EYFP
Pdgfra-CreER^T2
E
CTRL
Δ10/Δ10
Polr3b^fl
Polr3b^wt
5' UTR
Cre
ERT
Pdgfra-CreERT
Polr3bΔ10
F
Band | Allele | Size (bp)
i | Polr3b^fl | 519
ii | Polr3b^wt | 297
iii | Pdgfra-CreERT | 507
iv | Polr3b^fl | 945
v | Polr3b Δ10 | 351
Legend
5' loxP cassette
forward primer
3' loxP cassette
reverse primer

Figure S1

Supplementary Figure 2: CNS anatomy is relatively normal in ∆10 mice with the exception of visible ventral hypomyelination. **(A)** On gross inspection, ∆10/∆10 showed obvious and consistent differences in white matter content in the ventral hindbrain when compared with **(B)** +/∆10 littermates at P14. Scale bar represents 2 mm, representative images shown. **(C-D)** Using standard hematoxylin and eosin staining on sections prepared from P14 brains, we did not observe obvious differences in histological features such as cellularity (number of nuclei) within areas corresponding to major CNS white matter tracts (C: ∆10/∆10, D: +/∆10 littermate). Scale bar represents 1 mm. **(E, F)** The large box indicates the ventral pontomedullary area comparable to A, B, magnified 6X. Scale bar represents 300 micrometers. **(G)** Overall histology appears comparable on H&E staining among transgenic mice used in the study. **Top panel:** ∆10/∆10 mice (*Pdgfra-CreER*;*Polr3b*$^{fl/fl}$) body of the corpus callosum in the peri-hippocampal area (left), arbor vitae of the cerebellum (center) and putaminal pencils of the striatum (right) shown. **Middle panel:** +/∆10mice (*Pdgfra-CreER*;*Polr3b*$^{+/fl}$) body of the corpus callosum in the perihippocampal area (left), arbor vitae of the cerebellum (center) and putaminal pencils of the striatum (right) shown. **Lower panel:** CTRL mice (*Polr3b*$^{+/fl}$) body of the corpus callosum in the perihippocampal area (left), arbor vitae of the cerebellum (center) and putaminal pencils of the striatum (right) shown.

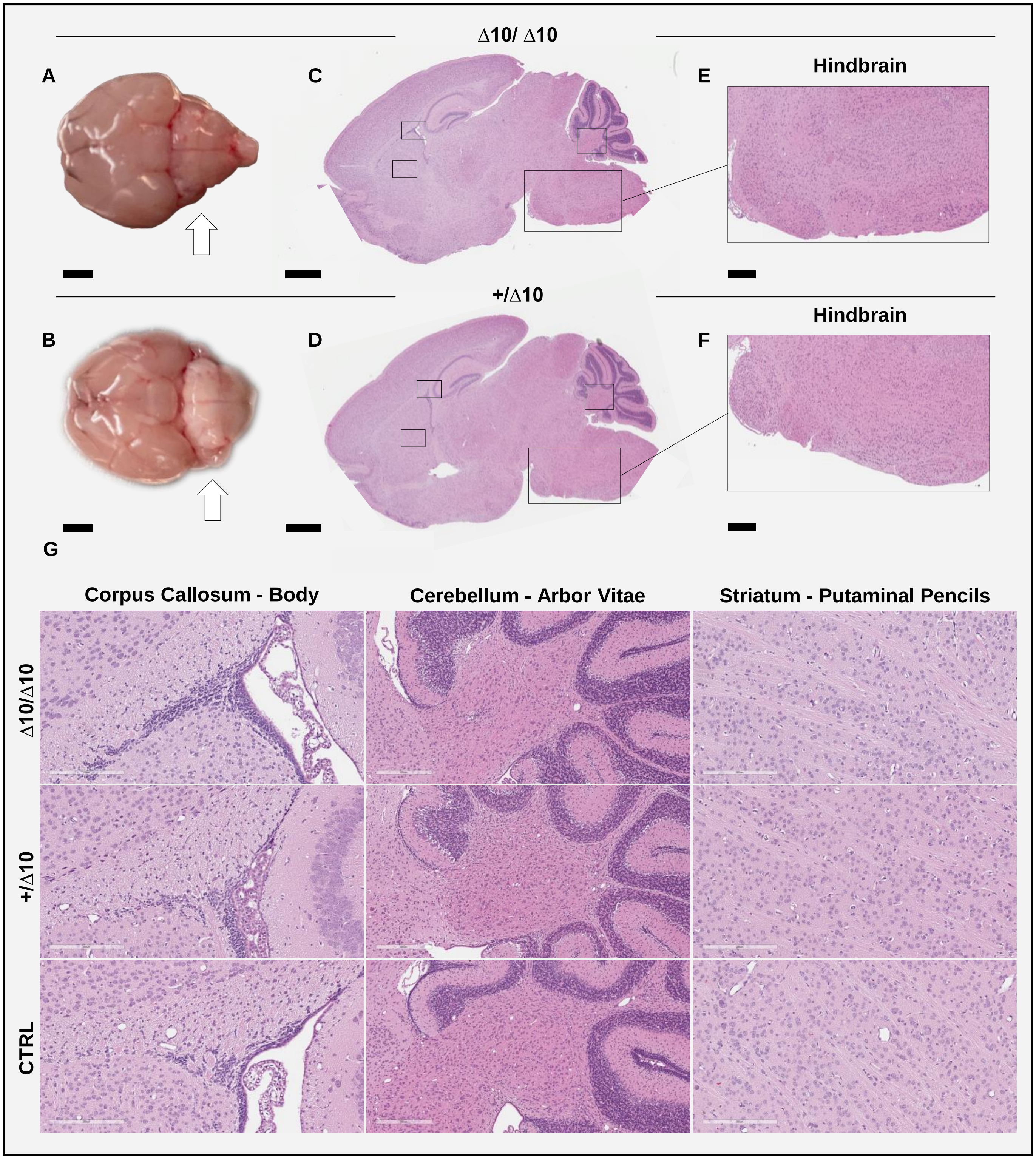

Δ10/ Δ10
A
C
E
Hindbrain
+/Δ10
B
D
F
Hindbrain
G
Corpus Callosum - Body
Cerebellum - Arbor Vitae
Striatum - Putaminal Pencils
Δ10/Δ10
+/Δ10
CTRL

Figure S2

Supplementary Figure 3: Myelination is reduced along the rostro-caudal axis of the brain in Δ10 mice. (A) Pooled quantification of Mbp immunofluorescence intensity in the corpus callosum at four levels of section (alpha, beta, gamma, delta) reveals that Δ10/Δ10 mice have reduced Mbp overall as compared to CTRL and +/Δ10 mice. Individual points in the graph represent biological replicates. Statistics in (A) were determined using Brown-Forsythe and Welch One-Way ANOVA followed by multiple comparisons testing using the Dunnett T3 method with $\alpha=0.05$. * $p<0.05$, ** $p<0.01$, *** $p<0.001$, **** $p<0.0001$. **(B)** Analysis of Mbp immunofluorescence intensity in the corpus callosum at each of the section levels confirmed that Δ10/Δ10 mice have consistently reduced Mbp expression as compared to littermates along the rostro-caudal axis. Statistics in (B) were computed using two-way ANOVA (Level of Section versus Genotype) with matching among sections from the same animal. Level of section was not significant ($p=0.2065$), but genotype was significant ($p=0.0013$). Interaction between level of section and genotype was not significant ($p=8956$). Multiple comparisons between genotype groups were tested post-hoc using the Tukey method and $\alpha=0.05$.

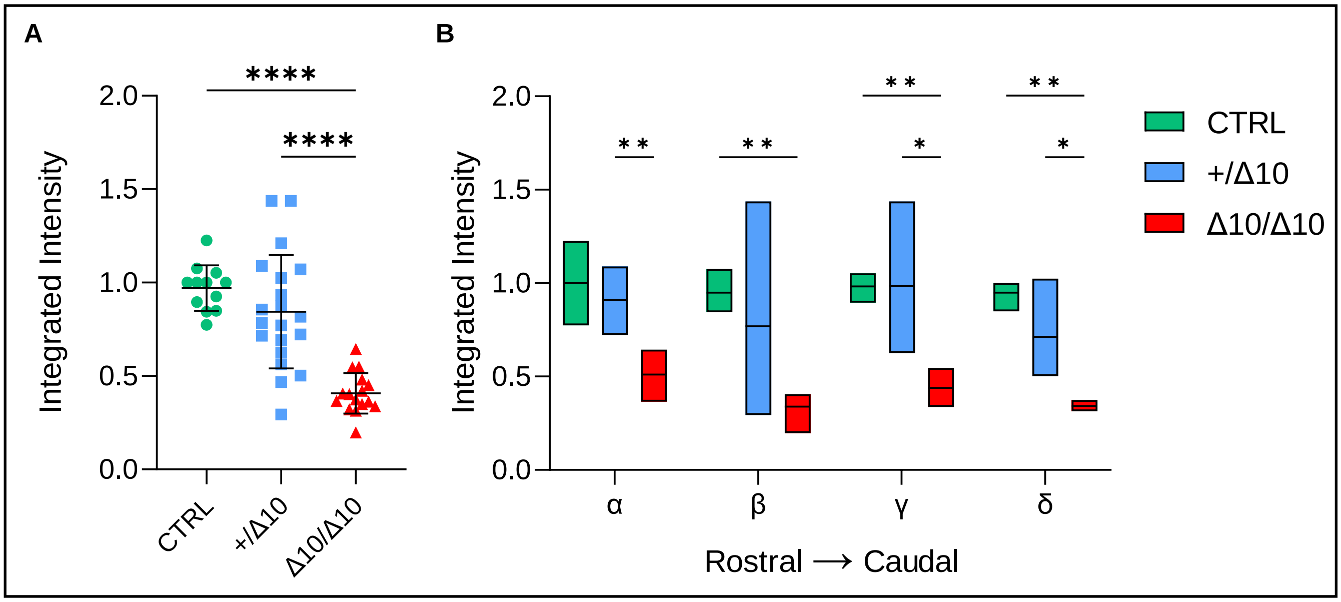
A
B
Integrated Intensity
2.0
1.5
1.0
0.5
0.0
****
****
CTRL
+/Δ10
Δ10/Δ10
**
**
**
*
**
*
α
β
γ
δ
Rostral ⟶ Caudal
CTRL
+/Δ10
Δ10/Δ10

Figure S3

Supplementary Figure 4: *Ex vivo* magnetic resonance imaging reveals hypomyelination in Δ10 mice. (A-D) Anatomical scans (left, grey) and myelin water fraction (MWF) maps (right, blue/grey gradient) were obtained from four individual mice from each of the following groups: **(A)** Δ10/Δ10, **(B)** +/Δ10, **(C)** CTRL, and **(D)** Tamoxifen-naïve Pdgfrα-CreERT; Polr3b$^{fl/fl}$ (untreated controls). **(E)** Reference atlas scans were used for spatial registration and manually delineating regions of interest (ROIs).[14] **(F)** +/Δ10 (blue) and Δ10/Δ10 (red) were compared at individual ROIs where average MWF values were reduced in Δ10/Δ10s relative to +/Δ10s. **(G)** Aggregate MWF values were obtained by summing the average MWF values from each region in each genotype to describe overall myelination status. Standard deviation of the aggregate value was obtained by error propagation, taking the square root of the sum of the variances from each ROI included in the aggregate sum.

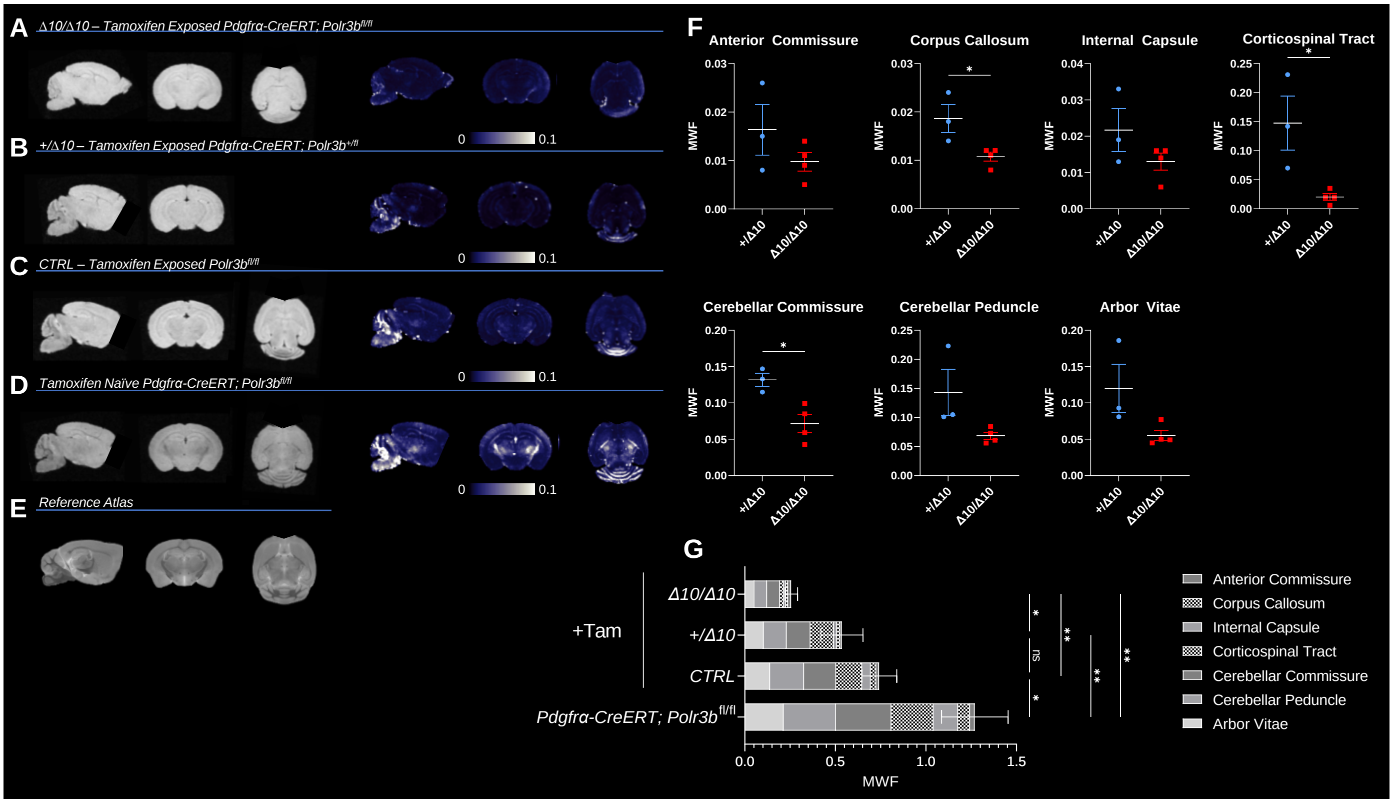
A Δ10/Δ10 – Tamoxifen Exposed Pdgfrα-CreERT; Polr3b^fl/fl
B +/Δ10 – Tamoxifen Exposed Pdgfrα-CreERT; Polr3b^+/fl
C CTRL – Tamoxifen Exposed Polr3b^fl/fl
D Tamoxifen Naïve Pdgfrα-CreERT; Polr3b^fl/fl
E Reference Atlas
0 0.1
F
Anterior Commissure
Corpus Callosum
Internal Capsule
Corticospinal Tract
Cerebellar Commissure
Cerebellar Peduncle
Arbor Vitae
MWF
+/Δ10
Δ10/Δ10
G
+Tam
Δ10/Δ10
+/Δ10
CTRL
Pdgfrα-CreERT; Polr3b^fl/fl
MWF
Anterior Commissure
Corpus Callosum
Internal Capsule
Corticospinal Tract
Cerebellar Commissure
Cerebellar Peduncle
Arbor Vitae

Figure S4

Supplementary Figure 5: Luxol Fast Blue (LFB) staining reveals reduced myelination in ∆10 mice relative to Cre negative controls. (A) Representative images of corpus callosum LFB stains. **(B)** Quantitation of the relative stained area of the corpus callosum revealed reduced myelination in +/∆10s and ∆10/∆10s relative to CTRLs. The average difference between +/∆10s and ∆10/∆10s was not statistically significant. **(C)** Representative images of cerebellar LFB stains. **(D)** Quantitation of the relative stained area of the cerebellum revealed reduced myelination in ∆10/∆10relative to CTRLs. The average difference between +/∆10s and ∆10/∆10s was not statistically significant.

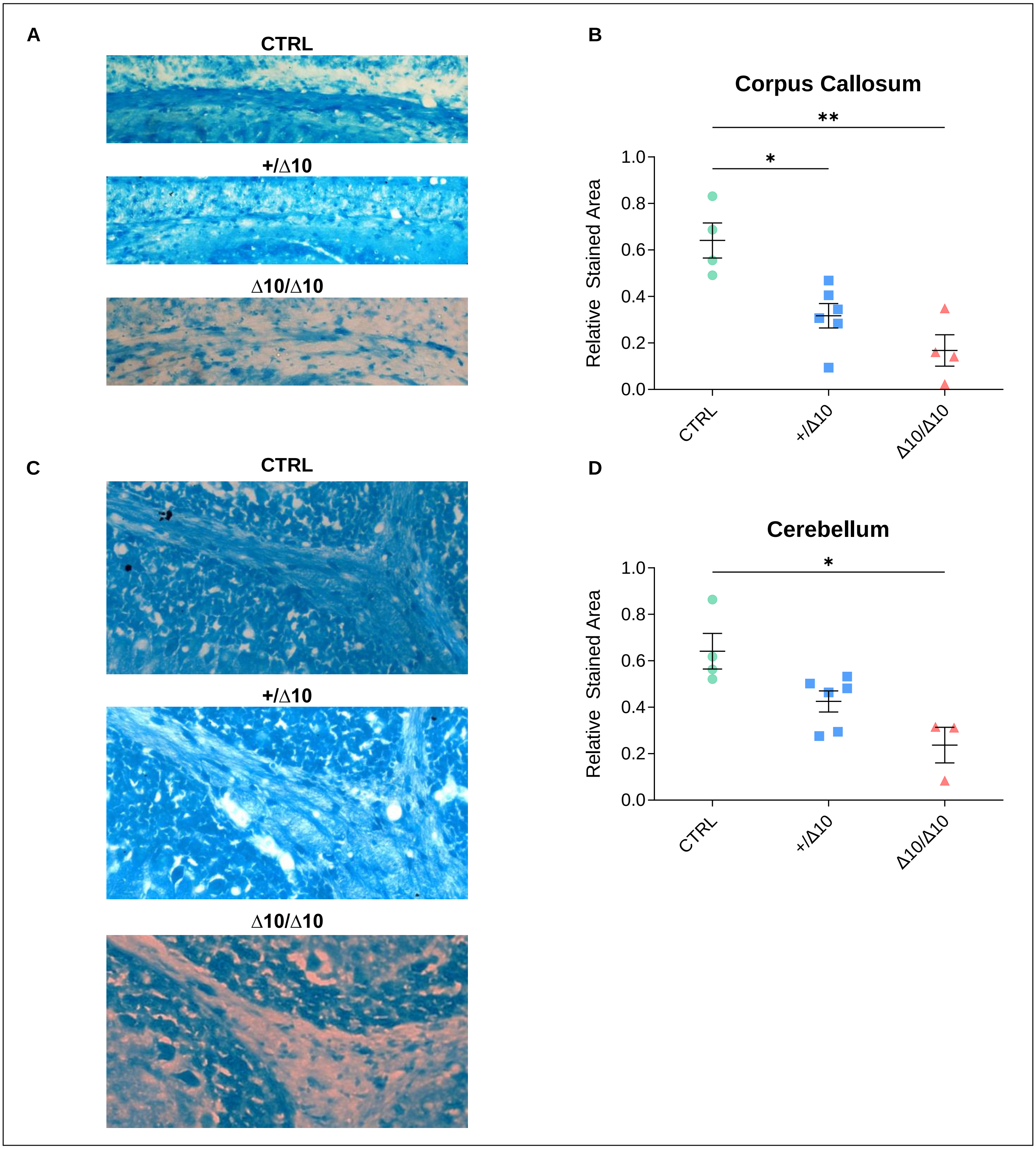
A
CTRL
+/Δ10
Δ10/Δ10
B
Corpus Callosum
**
*
Relative Stained Area
1.0
0.8
0.6
0.4
0.2
0.0
CTRL
+/Δ10
Δ10/Δ10
C
CTRL
+/Δ10
Δ10/Δ10
D
Cerebellum
*
Relative Stained Area
1.0
0.8
0.6
0.4
0.2
0.0
CTRL
+/Δ10
Δ10/Δ10

Figure S5

Supplementary Figure 6: Regional time-series analysis of Pdgfrα+ cell population dynamics in the mouse brain. Overall population dynamics were consistent with the changes observed at the whole-brain level in transgenic mice. **(A-H)** Single Pdgfrα+ cell counts and confidence intervals for the two-way ANOVA statistical analysis revealed statistically significant decreases in single-positive Pdgfrα+ cell numbers at P14 in the thalamus, hypothalamus, midbrain, and pons of Δ10/Δ10s relative to +/Δ10s. **(J-R)** Double YFP+ Pdgfrα+ cell counts and confidence intervals for the two-way ANOVA statistical analysis revealed statistically significant decreases in double-positive cell numbers at P14 in nearly all brain regions of Δ10/Δ10s relative to +/Δ10s. On time-series plots (at left on each panel), individual points represent the mean cell density of a given genotype group, error bars represent standard deviation. On multiple comparisons plots, individual points represent group mean differences, error bars represent the 95% confidence intervals.

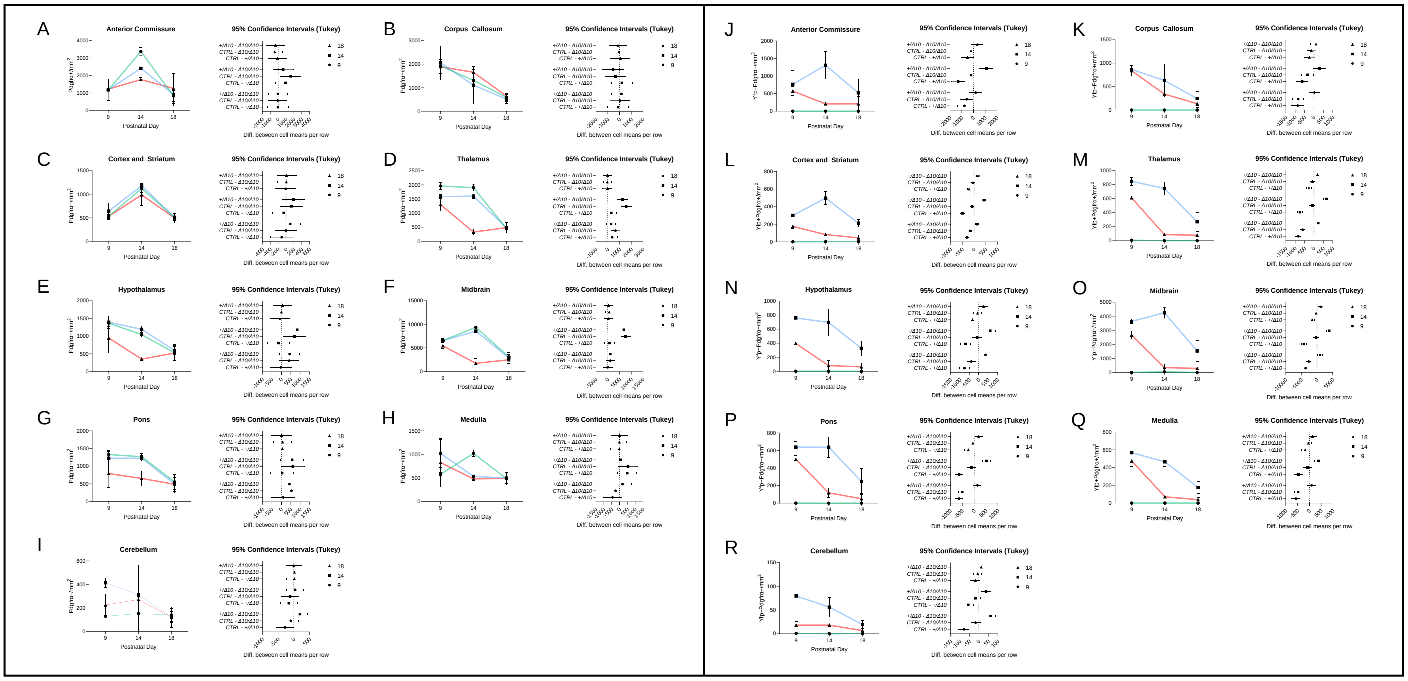
A
Anterior Commissure
95% Confidence Intervals (Tukey)
B
Corpus Callosum
95% Confidence Intervals (Tukey)
C
Cortex and Striatum
95% Confidence Intervals (Tukey)
D
Thalamus
95% Confidence Intervals (Tukey)
E
Hypothalamus
95% Confidence Intervals (Tukey)
F
Midbrain
95% Confidence Intervals (Tukey)
G
Pons
95% Confidence Intervals (Tukey)
H
Medulla
95% Confidence Intervals (Tukey)
I
Cerebellum
95% Confidence Intervals (Tukey)
J
Anterior Commissure
95% Confidence Intervals (Tukey)
K
Corpus Callosum
95% Confidence Intervals (Tukey)
L
Cortex and Striatum
95% Confidence Intervals (Tukey)
M
Thalamus
95% Confidence Intervals (Tukey)
N
Hypothalamus
95% Confidence Intervals (Tukey)
O
Midbrain
95% Confidence Intervals (Tukey)
P
Pons
95% Confidence Intervals (Tukey)
Q
Medulla
95% Confidence Intervals (Tukey)
R
Cerebellum
95% Confidence Intervals (Tukey)
Postnatal Day
Diff. between cell means per row

Figure S6

Supplementary Figure 7: Regional time-series analysis of CC1+ cell population dynamics in ∆10 brains. Overall population dynamics were consistent with the changes observed at the whole-brain level in transgenic mice. **(A-H)** Single CC1+ cell counts and confidence intervals for the two-way ANOVA statistical analysis revealed statistically significant decreases in single-positive CC1+ cell numbers in ∆10 homozygotes relative to heterozygotes. **(J-R)** Double YFP+ CC1+ cell counts and confidence intervals for the two-way ANOVA statistical analysis revealed statistically significant decreases in double-positive cell numbers at P14 and P18. On time-series plots (at left on each panel), individual points represent the mean cell density of a given genotype group, error bars represent standard deviation. On multiple comparisons plots, individual points represent group mean differences, error bars represent the 95% confidence intervals.

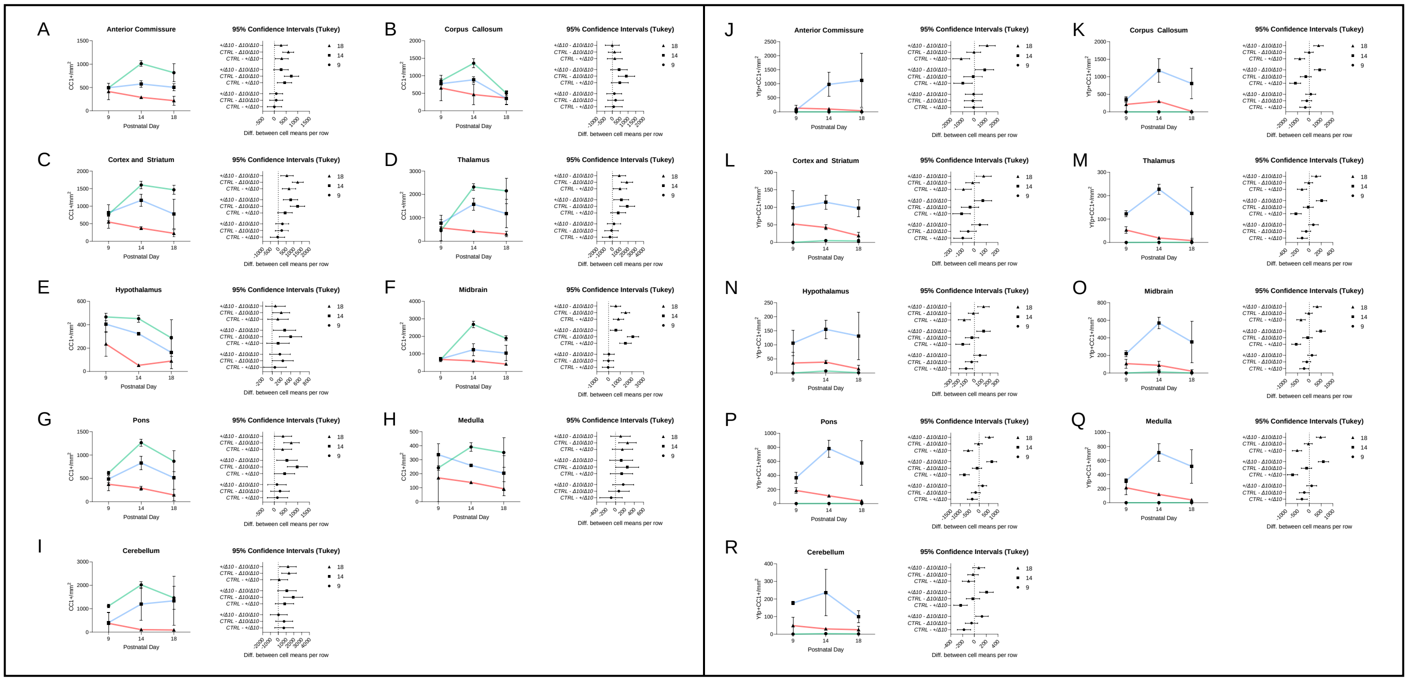

Figure S7

Supplementary Figure 8: **Cleaved Caspase 3 staining suggests that apoptosis is not the main mechanism of YFP+ cell depletion in ∆10 homozygous mice**. **(A-F)** ∆10 heterozygote and homozygote brain sections at P9, P14 and P18 were stained with an antibody raised against Cleaved Caspase 3 in order to detect apoptosis. Representative images from P9 brains are shown. We detected very low numbers of Cleaved Caspase 3 positive cells in all brain sections examined regardless of the genotype, suggesting that apoptosis is not the mechanism of YFP+ cell depletion in ∆10 homozygotes. Pictured: splenium (corpus callosum), genu (corpus callosum), striatum, midbrain, hindbrain, arbor vitae (cerebellum).

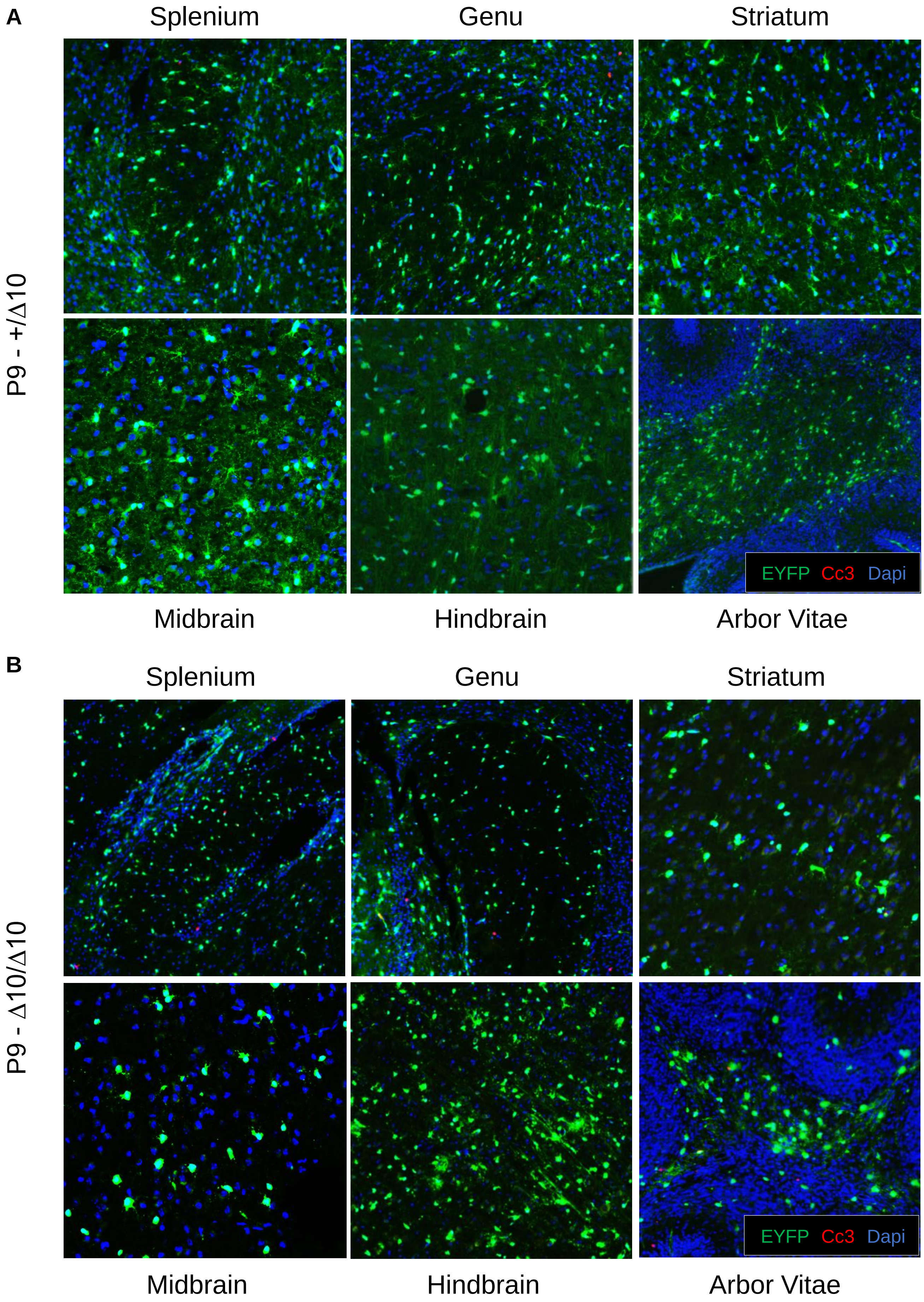
A
Splenium
Genu
Striatum
P9 - +/Δ10
Midbrain
Hindbrain
Arbor Vitae
EYFP Cc3 Dapi
B
Splenium
Genu
Striatum
P9 - Δ10/Δ10
Midbrain
Hindbrain
Arbor Vitae
EYFP Cc3 Dapi

Figure S8

Supplementary Figure 9: **Extended western blot quantification data. (A)** CTRL, +/Δ10, and Δ10/Δ10 brain lysates collected at P21 were subjected to SDS-PAGE and western blot (*see* Fig. 4H). (A) Pdgfrα expression was not significantly different between CTRL, +/Δ10, and Δ10/Δ10 mice. (B) Ng2 expression was significantly different between CTRL and Δ10/Δ10 mice. (C) Polr3a expression was not significantly different between CTRL, +/Δ10, and Δ10/Δ10 mice. (D) Polr3b expression was not significantly different between CTRL, +/Δ10, and Δ10/Δ10 mice. (E) Olig2 expression was significantly different between CTRL, +/Δ10, and Δ10/Δ10 mice (*see* Fig. 4J). (F) Mbp expression was significantly different between CTRL, +/Δ10, and Δ10/Δ10 mice. Individual points depict biological replicates (individual animals), mean and standard deviation are shown for each genotype group. Statistics were determined using Brown-Forsythe and Welch One-Way ANOVA followed by multiple comparisons testing using the Dunnett T3 method with $\alpha=0.05$. * $p<0.05$, ** $p<0.01$, *** $p<0.001$, **** $p<0.0001$.

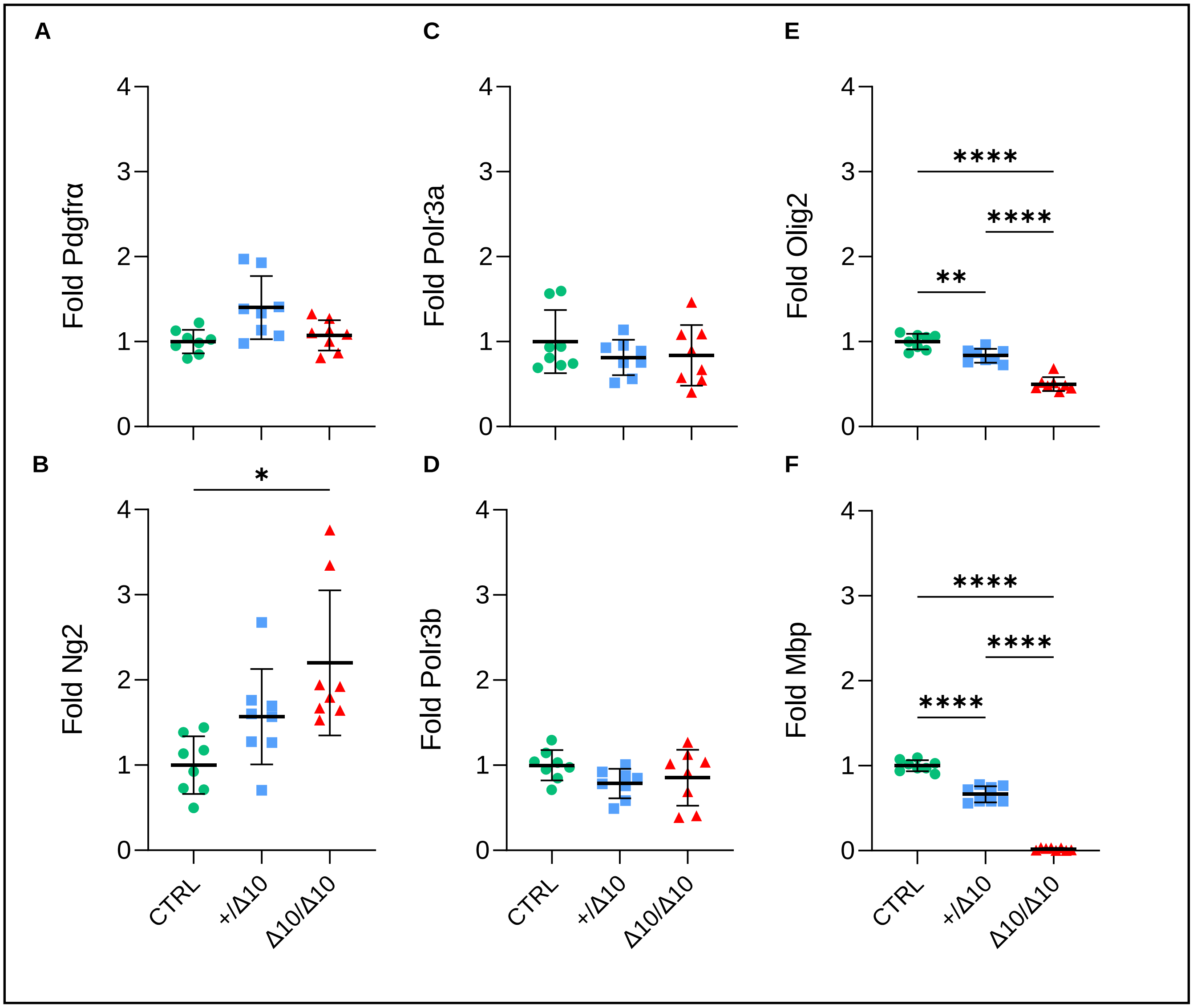
A
C
E
Fold Pdgfrα
Fold Polr3a
Fold Olig2
B
D
F
Fold Ng2
Fold Polr3b
Fold Mbp
0
1
2
3
4
*
**
****
CTRL
+/Δ10
Δ10/Δ10

Figure S9

Supplementary Figure 10: Extended proteomics western blot data. Three independent western blots of cytoplasmic and nuclear extracts from HEK293T cells transiently transfected with *p3XFLAG* (mock), wildtype *POLR3B-FLAG* (WT) or *POLR3BΔ10-FLAG* (Δ10) for 24 hours. An anti-FLAG antibody was used to detected FLAG-tagged POLR3B with an exposure time of **(A)** 5 seconds and **(B)** 30 seconds. Loading controls for the cytoplasmic (GAPDH) and nuclear (Lamin A, C) fractions are shown in Supplementary Figure 11 A) and B) respectively.

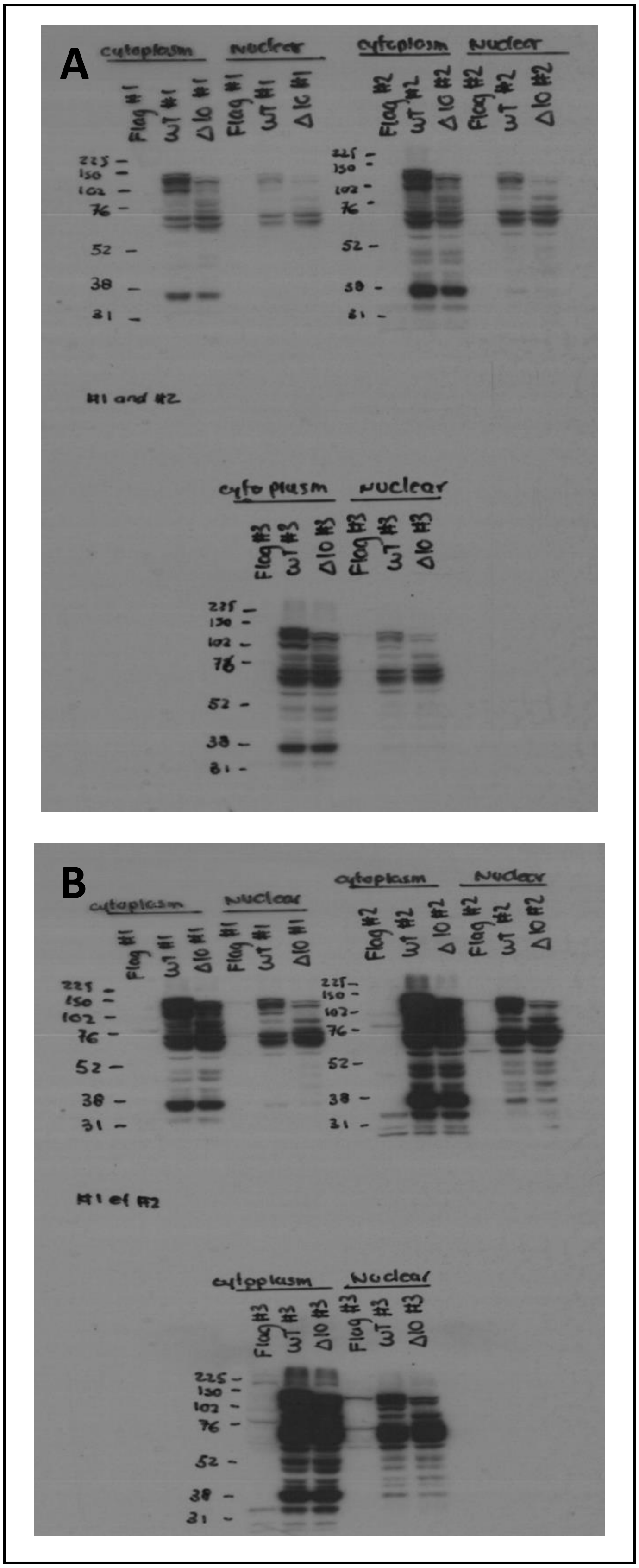
A
cytoplasm
nuclear
Flag #1
WT #1
Δ10 #1
Flag #1
WT #1
Δ10 #1
cytoplasm
nuclear
Flag #2
WT #2
Δ10 #2
Flag #2
WT #2
Δ10 #2
225
150
102
76
52
38
31
#1 and #2
cytoplasm
nuclear
Flag #3
WT #3
Δ10 #3
Flag #3
WT #3
Δ10 #3
B
cytoplasm
nuclear
Flag #1
WT #1
Δ10 #1
Flag #1
WT #1
Δ10 #1
cytoplasm
Nuclear
Flag #2
WT #2
Δ10 #2
Flag #2
WT #2
Δ10 #2
cytoplasm
Nuclear
Flag #3
WT #3
Δ10 #3
Flag #3
WT #3
Δ10 #3

Figure S10

Supplementary Figure 11: **Extended proteomics western blot data.** Three independent western blots of cytoplasmic and nuclear extracts from HEK293T cells transiently transfected with *p3XFLAG* (mock), wildtype *POLR3B-FLAG* (WT) or *POLR3BΔ10-FLAG* (Δ10) for 24h. Loading controls for the (A) cytoplasmic (GAPDH) and (B) nuclear (Lamin A, C) fractions are shown. Loading controls correspond to the same samples in Supplementary Figure 11. Unformatted blot images are provided in a separate data supplement.

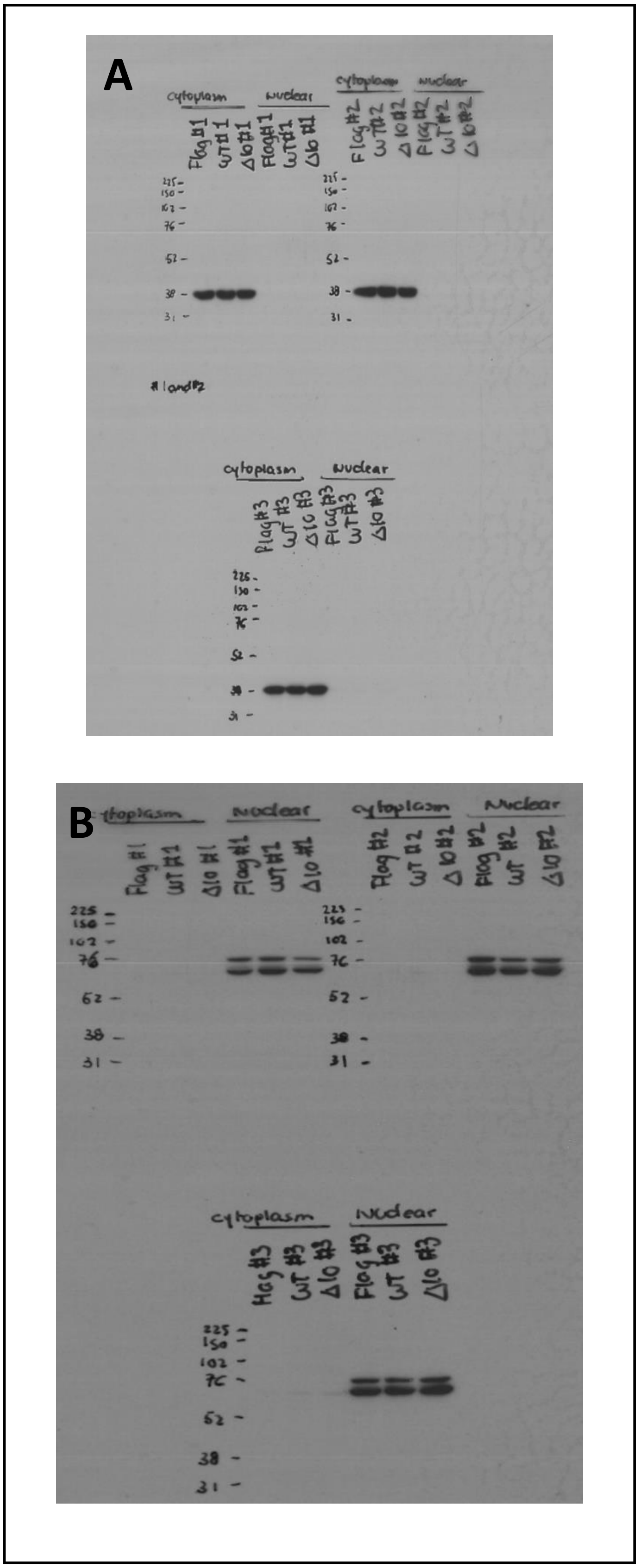
A
cytoplasm
nuclear
Flag#1
WT#1
Δ10#1
Flag#1
WT#1
Δ10#1
cytoplasm
nuclear
Flag#2
WT#2
Δ10#2
Flag#2
WT#2
Δ10#2
#1 and #2
cytoplasm
nuclear
Flag#3
WT#3
Δ10#3
Flag#3
WT#3
Δ10#3
B
cytoplasm
Nuclear
Flag#1
WT#1
Δ10#1
Flag#1
WT#1
Δ10#1
cytoplasm
Nuclear
Flag#2
WT#2
Δ10#2
Flag#2
WT#2
Δ10#2
225
150
102
76
52
38
31
cytoplasm
Nuclear
Flag#3
WT#3
Δ10#3
Flag#3
WT#3
Δ10#3

Figure S11

Supplementary Figures 12-23: **Extended animal model western blot data.** Unprocessed images from western blots quantified in Figure 4H-K (see Fig. S9 for extended quantification). For each experiment, pre-transfer and post-transfer total protein quantitation are shown on the top row, whereas the ladder and chemiluminescent images are shown on the bottom row. For each blot, the order of lanes is as follows from left-to-right: (1) Ladder; (2) CTRL #1; (3) CTRL #2; (4) CTRL #3; (5) CTRL #4; (6) +/Δ10 #1; (7) +/Δ10 #2; (8) +/Δ10 #3; (9) +/Δ10 #4; (10) Δ10/Δ10 #1; (11) Δ10/Δ10 #2; (12) Δ10/Δ10 #3; (13) Δ10/Δ10 #4; (14) $Mbp^{Shi/Shi}$ #1; (15) Ladder. **(Fig. S12)** Anti-Pdgfrα blot using samples from male mice. **(Fig. S13)** Anti-Ng2 blot using samples from male mice. **(Fig. S14)** Anti-Polr3a blot using samples from male mice. **(Fig. S15)** Anti-Polr3b blot using samples from male mice. **(Fig. S16)** Anti-Olig2 blot using samples from male mice. **(Fig. S17)** Anti-Mbp blot using samples from male mice. **(Fig. S18)** Anti-Pdgfrα blot using samples from female mice. **(Fig. S19)** Anti-Ng2 blot using samples from female mice. **(Fig. S20)** Anti-Polr3a blot using samples from female mice. **(Fig. S21)** Anti-Polr3b blot using samples from female mice. **(Fig. S22)** Anti-Olig2 blot using samples from female mice. **(Fig. S23)** Anti-Mbp blot using samples from female mice. Unformatted blot images are provided in a separate data supplement.

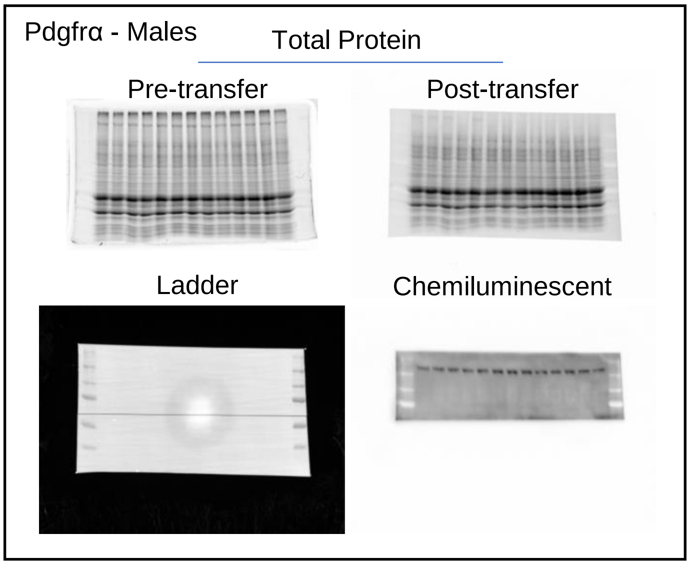

Pdgfrα - Males
Total Protein
Pre-transfer
Post-transfer
Ladder
Chemiluminescent

Figure S12

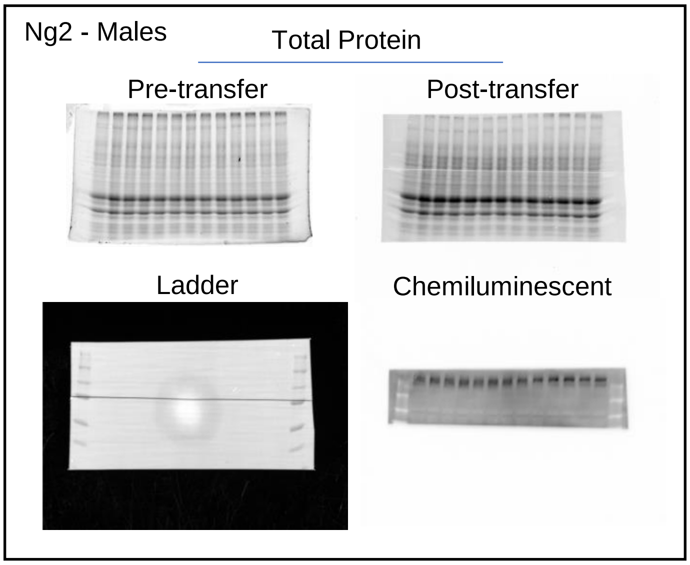
Ng2 - Males
Total Protein
Pre-transfer
Post-transfer
Ladder
Chemiluminescent

Figure S13

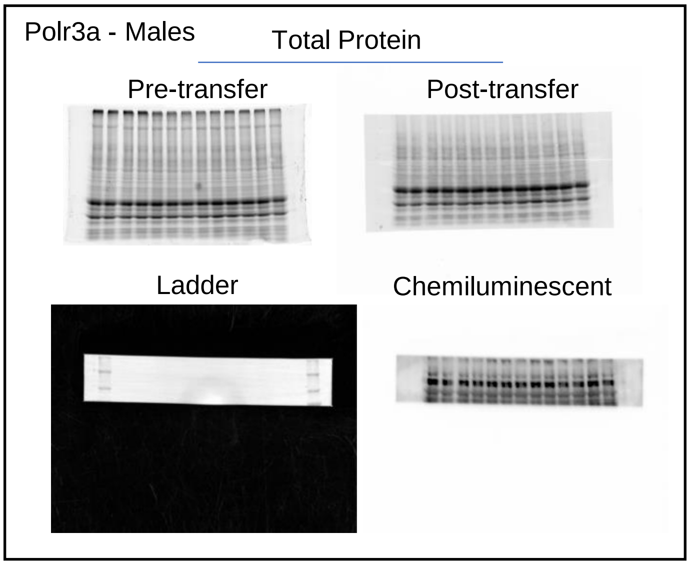
Polr3a - Males
Total Protein
Pre-transfer
Post-transfer
Ladder
Chemiluminescent

Figure S14

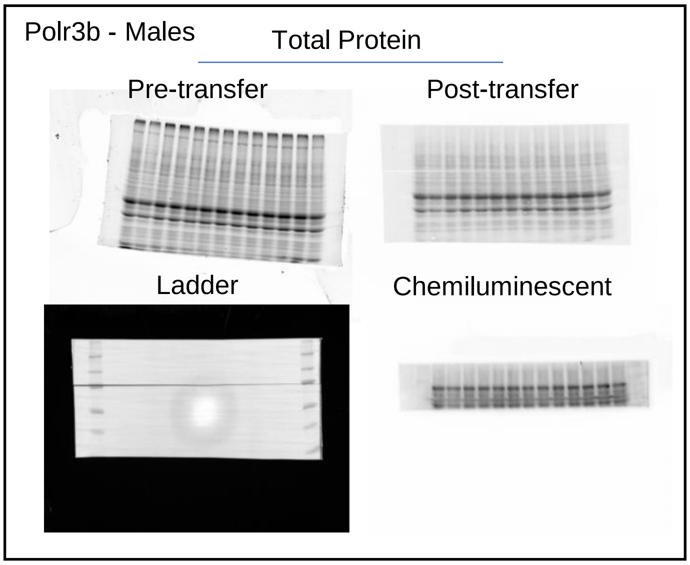
Polr3b - Males
Total Protein
Pre-transfer
Post-transfer
Ladder
Chemiluminescent

Figure S15

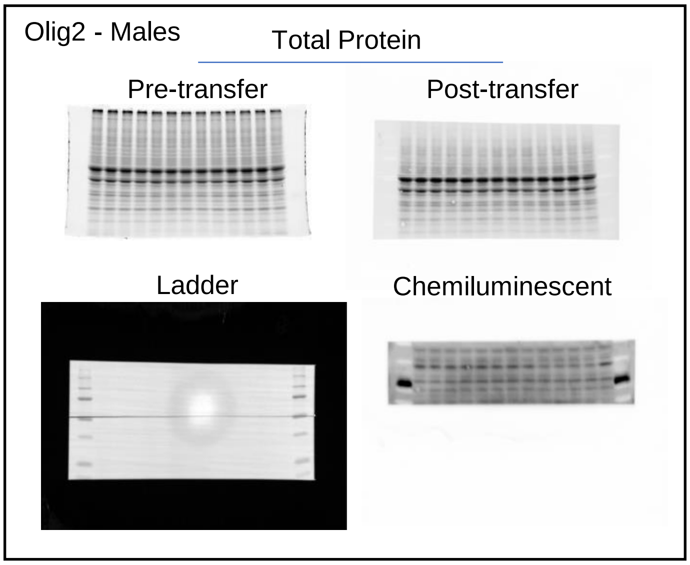
Olig2 - Males
Total Protein
Pre-transfer
Post-transfer
Ladder
Chemiluminescent

Figure S16

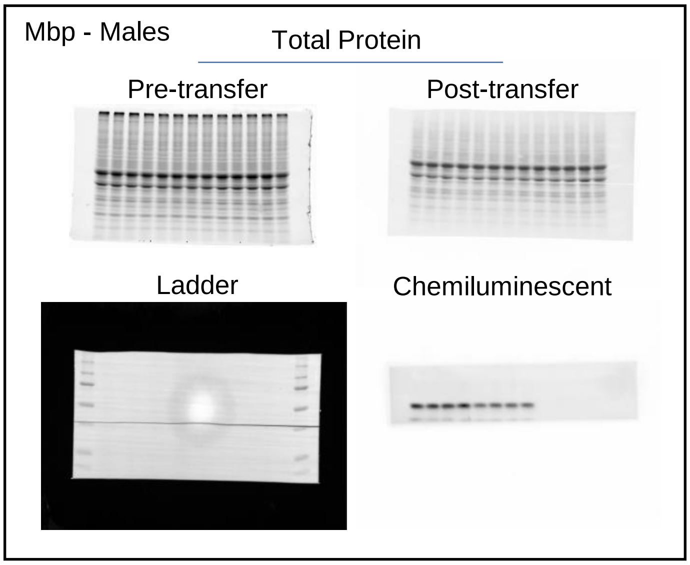
Mbp - Males
Total Protein
Pre-transfer
Post-transfer
Ladder
Chemiluminescent

Figure S17

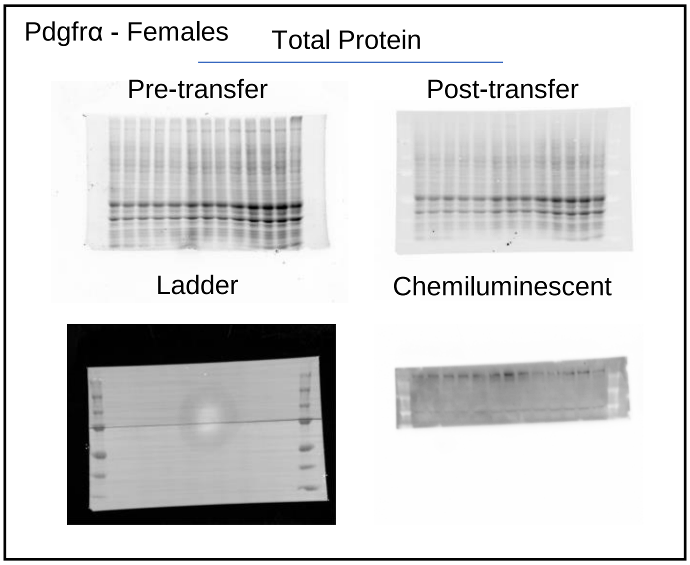
Pdgfrα - Females
Total Protein
Pre-transfer
Post-transfer
Ladder
Chemiluminescent

Figure S18

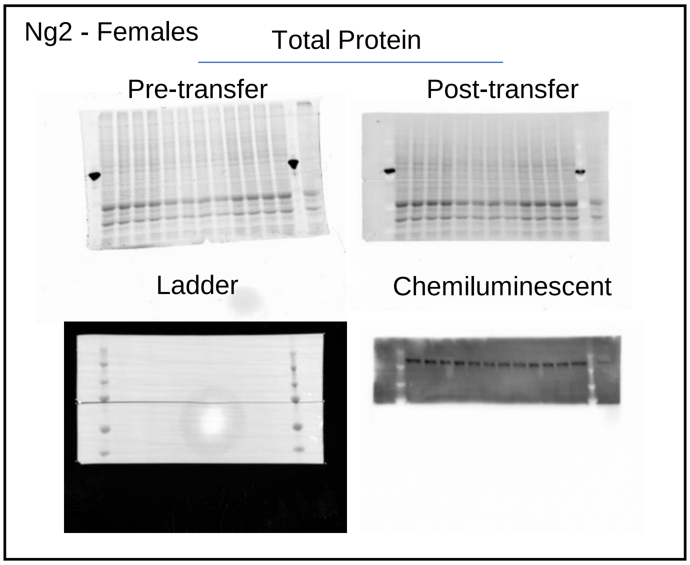
Ng2 - Females
Total Protein
Pre-transfer
Post-transfer
Ladder
Chemiluminescent

Figure S19

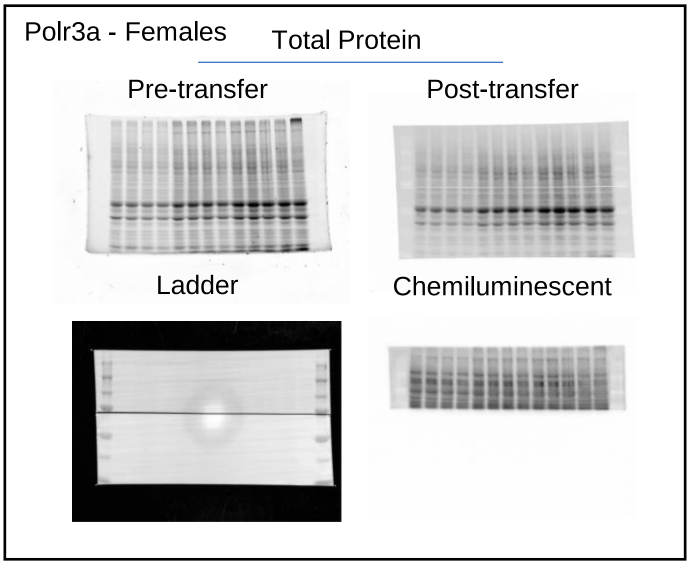
Polr3a - Females
Total Protein
Pre-transfer
Post-transfer
Ladder
Chemiluminescent

Figure S20

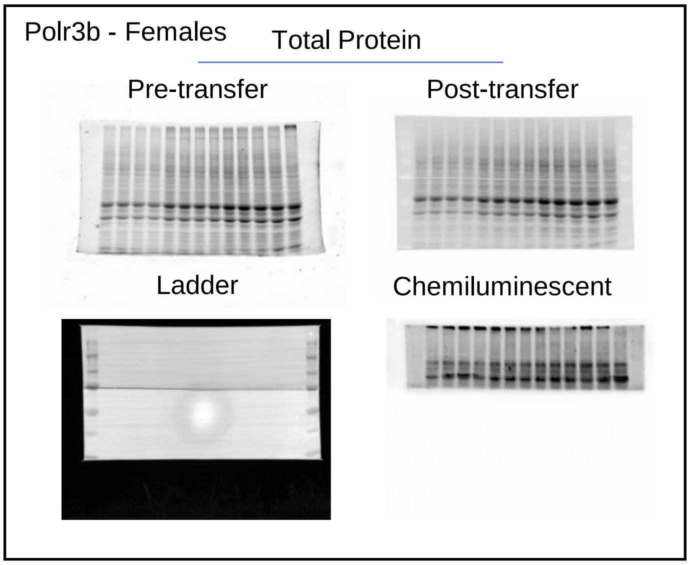
Polr3b - Females
Total Protein
Pre-transfer
Post-transfer
Ladder
Chemiluminescent

Figure S21

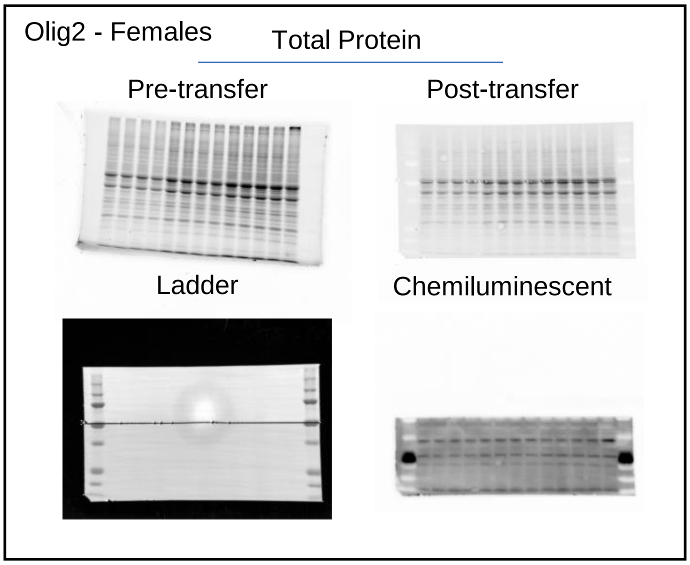
Olig2 - Females
Total Protein
Pre-transfer
Post-transfer
Ladder
Chemiluminescent

Figure S22

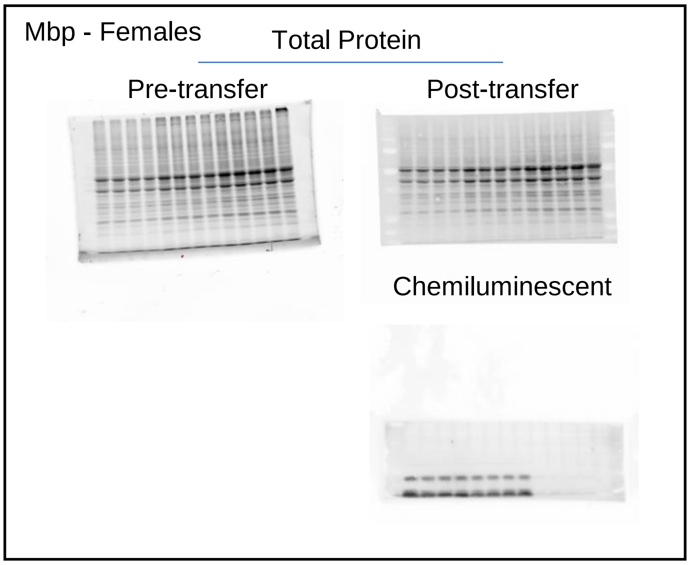
Mbp - Females
Total Protein
Pre-transfer
Post-transfer
Chemiluminescent

Figure S23

Supplementary Tables

Supplementary Table 1: Summary of antibodies and diluents used in western blotting experiments.

Antibody	Antibody Registry #	Diluent
Rb PDGFRa D1E1E (CST)	AB_2162345	1:1000 in 4% BSA/TBST (0.1% Tween)
Rb NG2 5320 (Millipore)	AB_11213678	1:1000 in 4% Milk/TBST (0.1% Tween)
Rb POLR3A 96328 (Abcam)	AB_10678851	1:1000 in 4% BSA/TBST (0.1% Tween)
M POLR3B 3G10 (Abnova)	AB_1204815	1:1000 in 4% BSA/TBST (0.1% Tween)
Rt MBP NB600 (Novus)	AB_2139899	1:500 in 4% BSA/TBST (0.1% Tween)
Gt OLIG2 2418 (R&D)	AB_2157554	1:1500 in 4% BSA/TBST (0.1% Tween)
Anti-Rabbit HRP (Novus)	AB_524675	1:5000 - 1:10000 in 4% Milk/TBST (0.1% Tween)
Anti-Mouse HRP (Novus)	AB_524800	1:15000 in 4% Milk/TBST (0.1% Tween)
Anti-Rat HRP (Novus)	AB_524662	1:5000 in 4% Milk/TBST (0.1% Tween)
Anti-Goat HRP (Novus)	AB_524752	1:10000 in 4% Milk/TBST (0.1% Tween)

Supplementary Table 2: MR imaging study animal information.

Litter	ID#	Experiment ID (Biogroup – N)	Scan Order	Biogroup	Genotype			
					Pdgfra-CreERT	Polr3bx10^{flx}	R26-Stop-EYFP	Tamoxifen Status
1	0113.1639.1562.4	1-1	1	Homozygous	+/cre	fl/fl	+/+	40 mg/kg P2-P5 q24h
1	0113.1639.1562.2	2-1	2	Heterozygous	+/cre	+/fl	+/+	40 mg/kg P2-P5 q24h
1	0113.1639.1562.3	3-1	3	Cre (-) Control	+/+	fl/fl	+/+	40 mg/kg P2-P5 q24h
4	0225.1710.1789.5	4-1	4	Tam (-) Control	+/cre	fl/fl	+/YFP	No Tamoxifen
2	0117.1584.1676/9.5	1-2	5	Homozygous	+/cre	fl/fl	+/+	40 mg/kg P2-P5 q24h
2	0117.1584.1676/9.7	2-2	6	Heterozygous	+/cre	+/fl	+/+	40 mg/kg P2-P5 q24h
2	0117.1584.1676/9.13	3-2	7	Cre (-) Control	+/+	+/fl	+/+	40 mg/kg P2-P5 q24h
4	0225.1710.1789.6	4-2	8	Tam (-) Control	+/cre	fl/fl	+/YFP	No Tamoxifen
2	0117.1584.1676/9.11	1-3	9	Homozygous	+/cre	fl/fl	+/+	40 mg/kg P2-P5 q24h
2	0117.1584.1676/9.17	2-3	10	Heterozygous	+/cre	+/fl	+/+	40 mg/kg P2-P5 q24h
2	0117.1584.1676/9.15	3-3	11	Cre (-) Control	+/+	+/fl	+/+	40 mg/kg P2-P5 q24h
4	0225.1710.1789.7	4-3	12	Tam (-) Control	+/cre	fl/fl	+/YFP	No Tamoxifen
3	0122.1709.1672.2	1-4	13	Homozygous	+/cre	fl/fl	+/+	40 mg/kg P2-P5 q24h
3	0122.1709.1672.6	2-4	14	Heterozygous	+/cre	+/fl	+/YFP	40 mg/kg P2-P5 q24h
3	0122.1709.1672.5	3-4	15	Cre (-) Control	+/+	+/fl	+/YFP	40 mg/kg P2-P5 q24h
5	0225.1639.1660/1.5	4-4	16	Tam (-) Control	+/cre	fl/fl	+/YFP	No Tamoxifen

All techniques used in the study were carried out on *ex vivo* mouse brains. Preparation of tissues was carried out as detailed above. Like coloured rows indicate mice obtained from the same litter. Tamoxifen-naïve mice were obtained from a separate, untreated litter in which all mice in the cage had never been exposed to tamoxifen.

Supplementary Table 3: Summary of animal numbers used for oligodendrocyte subpopulation (cell counting) analysis.

Antibody Panel	Time Point	No. Cre Negative Controls	No. ∆10 heterozygotes	No. ∆10 homozygotes	Figure
1	P9	2	3	3	6A-F
1	P14	2	2	3	6A-F
1	P18	3	6	3	6A-F
1	P18	NA	6	3	8D, F
2	P18	NA	6	3	8E
3	P9	NA	3	3	8A-C

Antibody panel 1: DAPI, YFP (Alexa 488), CC1 (Alexa 594) and Pdgfrα (Alexa 647); Antibody Panel 2: DAPI, YFP (Alexa 488) and Ng2 (Alexa 647); Antibody panel 3: DAPI, YFP (Alexa 488), Olig2 (Alexa 546) and Edu-647. Genotype groups are defined the same as elsewhere in the manuscript. The Cre negative control group included mice with both *Polr3bx10*$^{+/fl}$ and *Polr3bx10*$^{fl/fl}$ genotype status.

Supplementary Video Legends

Supplementary Video 1 (separate file).

Ataxia and tremor are evident in the Δ10/Δ10 mouse (smaller) relative to its +/Δ10 littermate (larger).

Supplementary Video 2 (separate file).

Characteristic wide foot angle is evident in a video of a Δ10/Δ10.

Supplementary Video 3 (separate file).

Δ10/Δ10 animals have spontaneous seizures. In this video, the animal (P12) displays characteristic seizure and small size.

Supplementary References

1. Kean MJ, Couzens AL, Gingras AC. Mass spectrometry approaches to study mammalian kinase and phosphatase associated proteins. *Methods*. Aug 2012;57(4):400-8. doi:10.1016/j.ymeth.2012.06.002
2. The UniProt Consortium. UniProt: the universal protein knowledgebase. *Nucleic acids research*. Jan 4 2017;45(D1):D158-D169. doi:10.1093/nar/gkw1099
3. The UniProt Consortium. UniProt: the universal protein knowledgebase. *Nucleic acids research*. 2018;46(5):2699-2699. doi:10.1093/nar/gky092
4. Tyanova S, Temu T, Sinitcyn P*, et al*. The Perseus computational platform for comprehensive analysis of (prote)omics data. *Nature methods*. Sep 2016;13(9):731-40. doi:10.1038/nmeth.3901
5. McKenzie IA, Ohayon D, Li H*, et al*. Motor skill learning requires active central myelination. *Science*. Oct 17 2014;346(6207):318-22. doi:10.1126/science.1254960
6. Gilda JE, Gomes AV. Stain-Free total protein staining is a superior loading control to β-actin for Western blots. *Analytical Biochemistry*. 2013/09/15/ 2013;440(2):186-188. doi:10.1016/j.ab.2013.05.027
7. Colella AD, Chegenii N, Tea MN, Gibbins IL, Williams KA, Chataway TK. Comparison of Stain-Free gels with traditional immunoblot loading control methodology. *Anal Biochem*. Nov 15 2012;430(2):108-10. doi:10.1016/j.ab.2012.08.015
8. Gürtler A, Kunz N, Gomolka M*, et al*. Stain-Free technology as a normalization tool in Western blot analysis. *Anal Biochem*. Feb 15 2013;433(2):105-11. doi:10.1016/j.ab.2012.10.010
9. Stirling DR, Swain-Bowden MJ, Lucas AM, Carpenter AE, Cimini BA, Goodman A. CellProfiler 4: improvements in speed, utility and usability. *BMC Bioinformatics*. Sep 10 2021;22(1):433. doi:10.1186/s12859-021-04344-9
10. Stirling DR, Carpenter AE, Cimini BA. CellProfiler Analyst 3.0: Accessible data exploration and machine learning for image analysis. *Bioinformatics (Oxford, England)*. Sep 3 2021;doi:10.1093/bioinformatics/btab634
11. McQuin C, Goodman A, Chernyshev V*, et al*. CellProfiler 3.0: Next-generation image processing for biology. *PLoS biology*. Jul 2018;16(7):e2005970. doi:10.1371/journal.pbio.2005970
12. Dobson ETA, Cimini B, Klemm AH, Wählby C, Carpenter AE, Eliceiri KW. ImageJ and CellProfiler: Complements in Open-Source Bioimage Analysis. *Curr Protoc*. May 2021;1(5):e89. doi:10.1002/cpz1.89
13. Dao D, Fraser AN, Hung J, Ljosa V, Singh S, Carpenter AE. CellProfiler Analyst: interactive data exploration, analysis and classification of large biological image sets. *Bioinformatics (Oxford, England)*. Oct 15 2016;32(20):3210-3212. doi:10.1093/bioinformatics/btw390

14. Dorr AE, Lerch JP, Spring S, Kabani N, Henkelman RM. High resolution three-dimensional brain atlas using an average magnetic resonance image of 40 adult C57Bl/6J mice. *NeuroImage*. 2008/08/01/ 2008;42(1):60-69. doi:https://doi.org/10.1016/j.neuroimage.2008.03.037
